# Unveiling centromeric retrotransposon dynamics through a high-quality rye genome assembly

**DOI:** 10.1038/s41467-026-76753-4

**Published:** 2026-08-15

**Authors:** Erwang Chen, Carlotta Marie Wehrkamp, Srijan Jhingan, Thomas Lux, Zihao Zhu, Jianyong Chen, Zuzana Tulpová, Axel Himmelbach, Jörg Fuchs, Jia-Wu Feng, M. Timothy Rabanus-Wallace, Hana Šimková, Manuel Spannagl, Martin Mascher, Thomas Wicker, Andreas Houben, Nils Stein

**Affiliations:** 1 Leibniz Institute of Plant Genetics and Crop Plant Research (IPK), Seeland, Germany; 2 Department of Plant and Microbial Biology, University of Zurich, Zurich, Switzerland; 3 PGSB–Plant Genome and Systems Biology, Helmholtz Center Munich–German Research Center for Environmental Health, Neuherberg, Germany; 4 Institute of Experimental Botany of the Czech Academy of Sciences, Olomouc, Czech Republic; 5 School of Agriculture, Forestry, and Ecosystem Science (SAFES), The University of Melbourne, Parkville, VIC Australia; 6 German Centre for Integrative Biodiversity Research (iDiv) Halle-Jena-Leipzig, Leipzig, Germany; 7 Crop Plant Genetics, Institute of Agricultural and Nutritional Sciences, Martin Luther University Halle-Wittenberg, Halle (Saale), Germany

**Keywords:** Plant genetics, Genome, Agricultural genetics, Mobile elements

## Abstract

Rye (*Secale cereale* L.) is an important cereal crop known for its high yield potential and tolerance to biotic and abiotic stresses. However, its large, repeat-rich, and heterozygous genome has posed challenges for assembly compared to related species such as wheat and barley. Here, we present a high-quality, chromosome-scale genome assembly of the inbred line Lo7, generated using PacBio HiFi, Oxford Nanopore, Hi-C, and BioNano technologies with the TRITEX pipeline. The resulting Lo7_V3 assembly spans 6.76 Gb with a contig N50 of 128 Mb, correcting previous misorientations and fully assembling all seven centromeres. Repetitive clusters containing rye-specific satellite sequences (pSc200 and pSc250) are contiguously assembled. Their chromosomal positions are validated using FISH. Centromeric retrotransposon analysis reveals *RLG_Abia* and *RLG_Abigail* as abundant, recently active elements, unlike in wheat. Collectively, the Lo7_V3 genome assembly provides an improved genomic resource for future genomic research in rye and related cereal species.

## Abstract (web summary)

The large, repeat-rich, and highly heterozygous rye (*Secale cereale* L.) genome has posed significant challenges for genome assembly. Here, the authors present an improved rye genome assembly and uncover unique retrotransposon organizations within its centromeres.

## Introduction

Rye is a diploid cereal crop within the tribe *Triticeae*, which poses unique challenges for genome assembly due to its large genome (haploid genome size, ∼7 Gb), high repeat content, and extensive structural heterogeneity^1^. The 1RS/1BL translocation, in which the short arm of rye chromosome 1R (1RS) replaces the short arm of wheat chromosome 1B, has been widely utilized in wheat breeding programs^2^. This translocation has contributed significantly to disease resistance, abiotic stress tolerance, and yield improvement. The introgression of rye chromatin, particularly from 1RS, has introduced beneficial alleles associated with resistance to rusts (*Sr31*, *Lr26* and *Yr9*)^3^, and powdery mildew (*Pm8*)^4^, making it one of the most successful examples of wide hybridization in modern wheat breeding. Despite its importance for improving stress tolerance and disease resistance in wheat, progress in rye genomics has been hindered by the lack of a high-quality reference genome. To date, highly contiguous genome assemblies for two rye inbred lines have been published: Lo7 and Weining^5,6^. While both assemblies represented significant breakthroughs for rye research and breeding, they suffered from gaps and structural inaccuracy, low repeat resolution, and missing centromere representation. Based on the advances in sequencing technology, it was our aim to overcome these shortcomings, ultimately advancing rye genomics.

Recent advancements in long-read single-molecule sequencing technologies such as PacBio HiFi and Oxford Nanopore, combined with techniques like high-throughput chromatin conformation capture (Hi-C) or optical map-based scaffolding, have facilitated the completion of complex genomes in humans and plants^7,8^. For instance, a complete Telomere-to-Telomere (T2T) genome assembly for *Arabidopsis thaliana* revealed comprehensive insights into centromeric regions, structural variations, and evolutionary dynamics^8^, while recent T2T (or near-complete) genome assemblies of crops like rice^9^, maize^10^, soybean^11^, oat^12^, cotton^13,14^, sorghum^15^, cucumber^16^, and wheat^17^ have significantly expanded our understanding of genome architecture and function. These achievements highlight the power of T2T assemblies in addressing previously unresolved genomic challenges, such as repetitive regions and structural variants, across diverse species. Given the recent success of complete assemblies in species such as wheat, an improved rye genome assembly also appears feasible and would provide stronger basis for studying rye genome organization and architecture.

The rye genome is exceptionally rich in repetitive sequences, with approximately 90% of its genome composed of various types of repeats—a proportion higher than that found in related *Triticeae* species such as barley and wheat^6,18^. For example, the nucleolar organizer region (NOR) on chromosome 1R contains large arrays of 45S rDNA tandem repeats, serving as the site for ribosomal RNA gene clusters and contributing significantly to the structural complexity^19^. Transposable elements (TEs), particularly long terminal repeat retrotransposons (LTR-RTs), like *Gypsy* and *Copia* superfamilies, dominate the rye genome and play a central role in genome size expansion and structural variation^5,6^. In addition to dispersed TEs, satellite sequences are widely distributed throughout the genome and are often organized in large blocks, contributing to heterochromatin formation. Likewise, rye-specific tandem repeats, pSc200, pSc250, and pSc119.2, are predominantly localized in the subtelomeric regions of all chromosomes^20,21^. With this, the abundance and diversity of repetitive sequences in the rye genome not only reflect its complex evolutionary history but also pose substantial challenges for genome assembly.

Previous studies have shown that centromeres in rye are enriched with centromere-specific retrotransposons and satellite repeats^22,23^. The centromeric regions of the rye genome are predominantly composed of TEs, with LTR retrotransposons being the major components. Among these, the *Gypsy* superfamily is particularly abundant and plays a central role in shaping the structure of centromeric chromatin. Although *Copia* elements are also present, they are less prevalent compared to *Gypsy* elements. The high density and accumulation of *Gypsy*-type retrotransposons in the centromeres suggest their importance in centromere function and evolution in rye. Two previous studies presented the analysis of near-complete centromere sequences in Einkorn wheat^24,25^, demonstrating that *Triticum monococcum* centromeres are primarily composed of two retrotransposon families, *RLG_Cereba* and *RLG_Quinta*, both of which belong to the *Gypsy* superfamily of LTR retrotransposons (CRM Clade)^26^. *RLG_Cereba* is the homolog of CRM (centromeric retrotransposons of maize)^27^ and CRR (centromeric retrotransposons of rice)^28^. It encodes a CR-domain fused to the integrase, which is proposed to guide the TE insertion into the functional centromere through recognition of centromeric chromatin^25,29,30^. Similarly, but independently, Tsukahara et al. recently suggested a targeted integration of the Copia element *Tal1* (*Transposon of Arabidopsis lyrata 1*, a copy of *ALE4*) into CENH3 chromatin in *Arabidopsis*^31^. *RLG_Quinta* is a non-autonomous element, lacking essential coding sequences such as reverse transcriptase and integrase, and thus relies on the enzymatic machinery provided by its autonomous partner, like *RLG_Cereba,* for its propagation (i.e., *RLG_Quinta* is a “parasite” of *RLG_Cereba*).

In this study, we present a chromosome-scale, near-complete genome assembly for the rye inbred variety Lo7, utilizing state-of-the-art sequencing and scaffolding technologies. Compared with previous versions, Lo7_V1^32^ and Lo7_V2^5^, this assembly (Lo7_V3) further improves the resolution of complex repetitive regions in subtelomeres and centromeres. We have improved the Lo7 genome by reducing assembly gaps, correcting orientation mistakes, and enhancing genome completeness.

## Results

### High-quality genome assembly of rye genotype Lo7

**Fig. 1:**
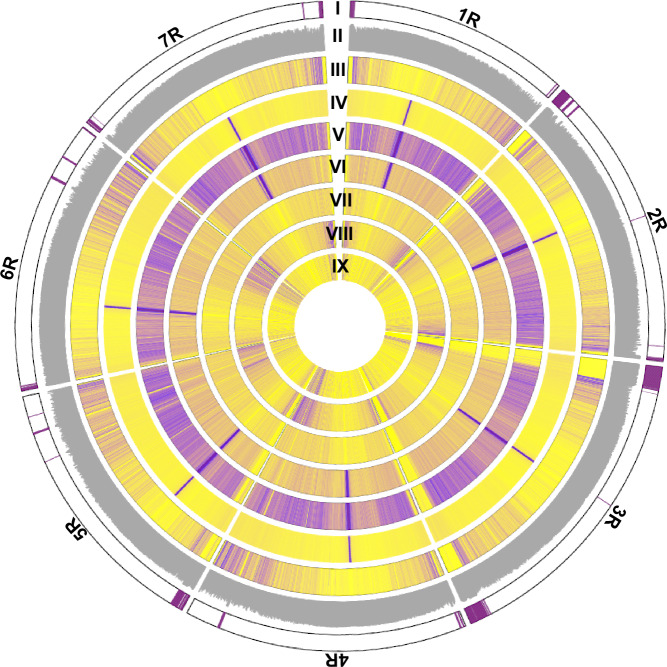
Assembly of the inbred line Lo7 genome. Circular display of the key characteristics of the Lo7_V3 genome assembly (plotted in Circos). The circles, from the outside to the inside, correspond to the following: (I) Densities [0–43] of tandem repeats (pSc200, pSc250, and pSc119.2). (II) 5mCpG methylation [0–110], averaged in 10 Mb windows. (III) Gene density [0–70], the density was computed using non-overlapping sliding windows of 10 Mb across the pseudomolecules. (IV) Centromeric peaks were validated using CENH3 ChIP-seq data. The values [0–0.4] represent the normalized signal intensity range where centromeric peaks were observed. (V– IX) Densities of transposable elements (TEs) in 10 Mb windows, including: (V) All TEs [0–153], (VI) LTR/*Gypsy* elements [0–77], (VII) LTR/*Copia* elements [0–77], (VIII) TIR elements [0–79], (IX) Helitron elements [0–77]. Color intensity in tracks III-IX ranges from yellow (low values), through blue (intermediate), to purple (high values), reflecting feature abundance or signal strength. Source data are provided as a Source Data file.

To generate a high-quality reference assembly for rye Lo7, we employed the TRITEX pipeline^33^, integrating data generated by multiple advanced sequencing technologies: PacBio HiFi reads (38-fold haploid coverage), ONT long reads (>25 kb, ninefold coverage), and Hi-C reads (20-fold coverage) for chromosome-level scaffolding (Supplementary Fig. 1a, and Supplementary Table 1). We estimated the heterozygosity of Lo7 using k-mer analysis, determining it to be 0.06% (Supplementary Fig. 1b). We performed flow cytometric genome size measurements using a recalculated estimate for the reference standard^5^, which resulted in a revised value of approximately 7 Gb for Lo7, indicating that the absolute genome size is smaller than that of previously assumed^34^ (Supplementary Table 2). The initial contigs were assembled by combining HiFi and ONT long reads using hifiasm^35^. These contigs were manually curated with Hi-C data to generate a draft assembly (Supplementary Fig. 2), which was further evaluated and refined using an optical genome map (Supplementary Fig. 3, and Supplementary Table 3). Finally, genome annotation was conducted using RNA-seq and Iso-seq data (Fig. 1). Additional features were systematically annotated, including chromosome lengths, repetitive sequences, centromere locations, and 5mCpG methylation (Fig. 1).

The final assembly spanned 6.76 Gb, with a contig N50 of 128 Mb and a GC content of 46.1% (Table 1). The lengths of the seven chromosomes ranged from 776 Mb to 1.1 Gb, which is 7.4% longer on average compared to each chromosome length in the earlier Lo7_V2 assembly (Supplementary Table 4). We annotated 75,850 protein-coding genes, including 43,754 high-confidence (HC) and 32,096 low-confidence (LC) genes (Table 1). LTR-RT annotation yielded an average LTR Assembly Index (LAI) of 23.47 and Merqury analysis showed a Quality Value (QV) score of 64.66, indicating high assembly quality (Supplementary Table 5). We performed the quantification of 45S rDNA by analyzing tandem repeats in single-molecule optical mapping data (Supplementary Table 3). We identified 5S and 45S rDNA arrays on chromosome 1R, confirming previous findings of the NOR on chromosome 1R (Supplementary Fig. 4). Moreover, by mapping telomere-specific short sequence motifs (TTTAGGG), we identified 6 of the expected 14 telomeres (Supplementary Fig. 4). Overall, we provided a Lo7_V3 genome assembly with high contiguity.

**Table 1:** Genome assembly comparisons between Lo7_V2^5^ and Lo7_V3

	Lo7_V2	Lo7_V3
Illumina (Gb)	947 (120x)	—
HiFi (Gb)	—	266.7 (38x)
ONT (Gb)	—	65.1 (>25 kb, 9x)
N50 (Mb)	15.2 (Scaffold)	128.0 (Contig)
Anchored (Gb)	6.21	6.68
Unanchored (Mb)	528.4	79.6
Completeness (%)	97.3	97.7
Total (Gb)	6.74	6.76
All_genes	57,222	75,850
HC_genes	34,441	43,754
LC_genes	22,781	32,096
HC_chrUn	1939	114
BUSCO_gene (%)	98.4	98.9
GC content (%)	46.0	46.1

Completeness of two assemblies was evaluated based on HiFi long reads. HC, high-confidence. LC, low-confidence. x indicates sequencing depth^52^.

### Lo7_V3 outperforms Lo7_V2 in assembly contiguity, orientation correction, and gene annotation quality

Lo7_V2 was assembled using high-coverage short reads (120-fold coverage), while Lo7_V3 incorporated both long-read HiFi and ONT sequencing technologies (Supplementary Fig. 5, and Supplementary Data 1). Although the overall genome length remained comparable, Lo7_V3 demonstrated substantial improvements in chromosomal anchoring, reducing unanchored regions. Assembly completeness was assessed through k-mer analysis of HiFi reads, showing a slight increase from 97.3% in Lo7_V2 to 97.7% in Lo7_V3 (Table 1). Gene annotation quality improved significantly, with more genes annotated and higher BUSCO scores for both HC and LC gene sets (Supplementary Table 6). The number of genes in unanchored regions decreased dramatically from 1939 in Lo7_V2 to just 114 in Lo7_V3 (Table 1). Following improvements in gene annotation, the BUSCO completeness score rose from 98.4% to 98.9% (Supplementary Table 6). Assembly contiguity was strongly enhanced, with the number of remaining gaps decreasing from 570 to 106 (Supplementary Table 4). Taken together, these results demonstrate that Lo7_V3 represents a substantial upgrade over Lo7_V2 in terms of contiguity, scaffolding accuracy, and annotation quality.

**Fig. 2:**
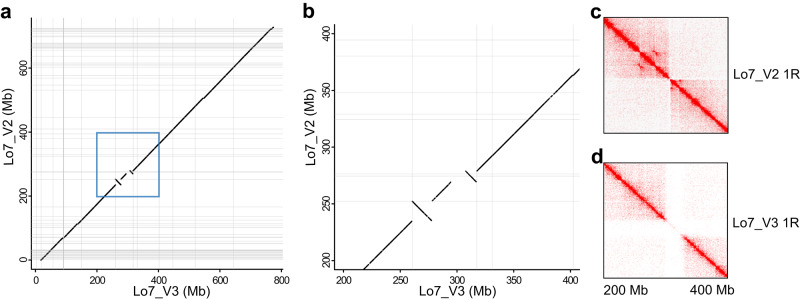
Orientation error corrections within chromosome 1 R. **a** Collinearity plot between Lo7_V2 (*y*-axis) and Lo7_V3 (*x*-axis). Grey lines indicate scaffold/contig junctions within chromosome 1R. The blue square indicates the region from 200 to 400 Mb. **b** Zoom-in (200–400 Mb) on 1R, showing two inversion-like orientation errors in Lo7_V2 in comparison within a single contig in Lo7_V3. Hi-C contact map of Lo7_V2 (**c**) and Lo7_V3 (**d**) on 1R (200–400 Mb). The region with low interactions represents the centromeric region within Lo7_V3 1R.

To evaluate orientation corrections, we generated collinearity plots comparing the scaffold-based Lo7_V2 and contig-based Lo7_V3 assemblies (Supplementary Fig. 6). Analysis revealed nine regions with orientation errors in Lo7_V2, all of which were resolved in Lo7_V3 (Supplementary Fig. 7). On chromosome 1R, Lo7_V2 contained two inversion-like orientation errors and three assembly gaps, particularly in the centromeric and pericentromeric regions (200–300 Mb) (Fig. 2a). These issues were corrected in Lo7_V3 within a single contig spanning the coordinate region between 260 Mb and 320 Mb of the respective chromosome sequence (Fig. 2b). Hi-C contact maps comparing both assemblies further provided validation of these structural improvements (Fig. 2c, d).

### Short tandem repeats form subtelomeric clusters

Consistent with previous findings in repeats annotation^6^, we revealed that 89.3% of the genome was annotated as repetitive sequences, including transposable elements (TEs) and other repeat fragments, which underscored the highly repetitive nature of the rye genome (Supplementary Fig. 8, and Supplementary Data 2). To verify the accuracy of the assembly, we designed chromosome barcode painting probes from the assembly and validated them using fluorescence in situ hybridization (FISH) (Supplementary Fig. 9a). These pooled oligoFISH probes were designed to paint specific segments on each chromosome. Single-copy sequences near the subtelomeric regions were selected and labeled with distinct fluorescent dyes (green and red), enabling precise identification and differentiation of each chromosome (Supplementary Fig. 9b).

**Fig. 3:**
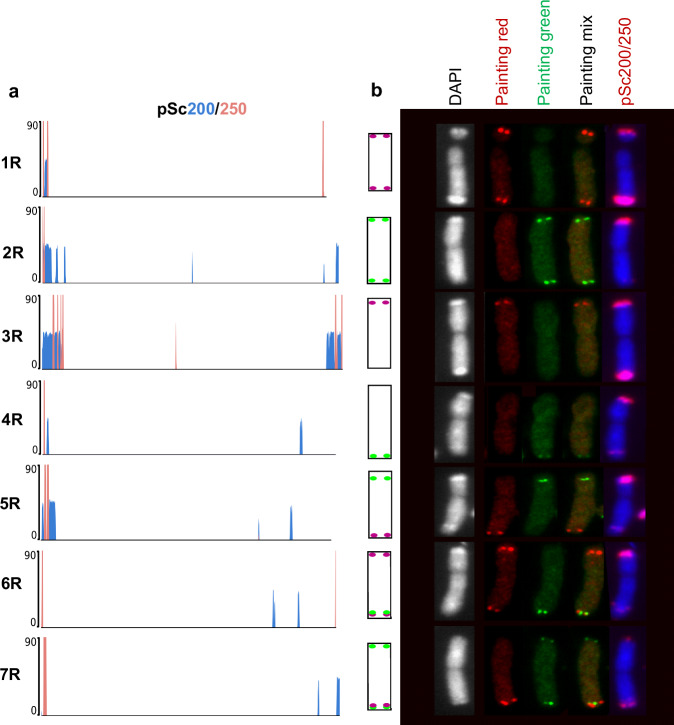
Genome assembly validations from in silico computation to FISH. **a** Identification of rye tandem repeats in the subtelomeric regions of each mitotic metaphase chromosome, including pSc200 (226.9 Mb, representing 3.40% of the genome size) and pSc250 (29.6 Mb, 0.44%). Frequency values range from 0 to 90. *x*-axis, chromosome position, *y*-axis, density of tandem repeats. **b** The FISH experiment revealed the signals of painting probes and pSc200/250 on each chromosome, with DAPI used as to stain chromatin. Schematic chromosomes show the in silico predicted probe positions as deduced in Supplementary Fig. 9a. The painting probes were labeled in green or red, allowing clear differentiation of each chromosome. pSc200 and pSc250 signals highlighted the subtelomeric regions on every chromosome. The alignment between FISH signals and the in silico predictions highlights the consistency between cytogenetic validation and computational genome modeling. Source data are provided as a Source Data file.

By integrating HiFi and ONT data, Lo7_V3 resolved complex regions containing rye satellite sequences, which reduced fragmentation within the unanchored regions compared to Lo7_V2 (Supplementary Fig. 10). We found that pSc119.2 (3.5 Mb, representing 0.05% of the genome size), pSc200 (226.9 Mb, 3.40%) and pSc250 (29.6 Mb, 0.44%) occupied the majority of these regions, particularly in the subtelomeric regions (Fig. 3a, and Supplementary Fig. 11). To verify the correct positioning of these repeats in the assembly, we also performed FISH experiments by using mixed probes, pSc200 and pSc250 as localization markers (Fig. 3b, and Supplementary Fig. 9b). By integrating the repeat and chromosome painting signals, we were able to determine the specific distribution of pSc200 and pSc250 on rye chromosomes. The observed FISH signals aligned well with the predicted distribution from the assembly, further confirming the consistency between the experimental results and computational simulations (Fig. 3b). These results indicate that Lo7_V3 significantly improved the anchoring of satellite sequences to the subtelomeric regions.

### Comparison of the Lo7 and Weining rye genomes

Recently, a new de novo version of the Weining rye genome, generated by comparable genome sequence data, became publicly available^34^ and provided, in principle, additional possibilities for benchmarking the quality of our Lo7_V3 assembly. Direct comparison of Lo7_V3 against the initial (Weining.V1)^6^ and the newly published Weining assemblies, however, revealed reduced quality of the Weining assemblies that would compromise direct benchmarking. Both assemblies showed redundant tracks of non-collapsed alternative haplotypes, as revealed by dotplot alignment of the assembled sequences (Supplementary Fig. 12), as well as assembly orientation errors, which could be detected by remapping of the publicly available Weining Hi-C data (Supplementary Fig. 13). Heterozygosity was evaluated as 0.54%, based on HiFi long reads (Supplementary Fig. 14). We therefore performed a de novo assembly of the publicly available datasets of Weining.V2 and obtained a highly contiguous phased assembly, representing two similarly sized but distinct haplotypes (H1 and H2) (Supplementary Fig. 15). Contiguity, size and genome composition statistics for Weining.V3 are highly similar and comparable to our Lo7_V3 assembly (Supplementary Figs. 16 and 17, Supplementary Tables 7–9, and Supplementary Data 3), thus providing additional independent support for the high quality of the Lo7_V3 assembly. Global alignment of Lo7_V3 against Weining.V3 H1 and H2 revealed overall a high collinearity of both genomes and clearly demonstrated that structural inconsistencies in the earlier Weining assemblies that were observed in comparison to Lo7_V3 were removed in the phased Weining assembly and were likely the result of unresolved haplotype differences in the collapsed haploid assemblies Weining.V1 and Weining.V2 (Supplementary Figs. 16–18).

### Structural analysis of seven complete centromeres

**Fig. 4:**
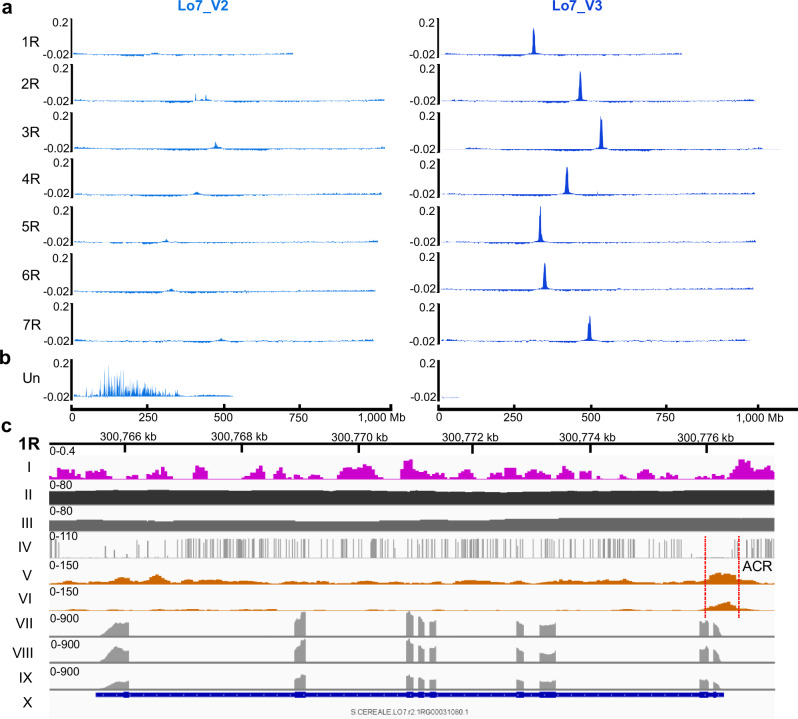
Identification of seven complete centromeres and in-depth structural analysis of a centromeric gene on chromosome 1R. **a**, **b** Mapping of CENH3 ChIP-seq data^22^ to the Lo7_V2 and Lo7_V3 sequence assemblies. Compared to the earlier Lo7_V2 assembly, the updated Lo7_V3 assembly (**a**) showed that CENH3 ChIP-seq signals were almost exclusively localized to the seven defined centromeric regions. The majority of CENH3 ChIP-seq reads were mapped to unanchored parts (Un) in Lo7_V2, indicating a highly fragmented centromeric landscape lacking chromosomal context (**b**). *x*-axis: chromosome position; *y*-axis: CENH3 ChIP-seq read density. **c** Detailed structural analysis of a gene encoding the eIF-2B subunit beta-like protein, located within the centromeric region of chromosome 1R.The analysis integrated multiple datasets, including CENH3 ChIP-seq (I), HiFi read mapping (II), ONT read mapping (III), 5mCpG methylation (IV), ATAC-seq1/2 (V-VI), and RNA-seq from three tissues, spike, root, and aerial organs (VII-IX)—along with high-confidence gene annotation (X). ACR, accessible chromatin region. All datasets were visualized using IGV.

Centromeres are notoriously difficult to assemble due to their highly repetitive nature^36^. Previous analyzes of rye centromeres have primarily relied on two published genome assemblies, both of which lack fully assembled centromeres for all seven chromosomes^22,37^. In Lo7_V2, centromere-associated regions were fragmented and mapped to unanchored regions (Fig. 4a, b). However, based on the CENH3 ChIP-seq data, the Lo7_V3 assembly achieved a significant breakthrough by assembling all seven centromeres, each accurately anchored to its respective chromosome (Fig. 4a, Supplementary Table 10, and Supplementary Fig. 19). We presented the predicted functional centromere lengths for chromosomes 1R to 7R to be in the range of approximately 9.6–11.6 Mb (Supplementary Table 11).

Gene content analysis identified eleven HC genes within five of the seven rye centromeres, while chromosome 4R and 6R centromeres were lacking any evidence of functional genes (Supplementary Data 4). The chromosome 1R centromere contained a gene encoding translation initiation factor eIF-2B subunit beta-like protein. To further validate the accuracy of genes annotated in the centromeric regions, we also integrated data from Transposase-Accessible Chromatin sequencing (ATAC-seq), 5mCpG methylation, HiFi and ONT read mapping, TE annotation and examined transcriptomic support for the annotation (Fig. 4b, and Supplementary Fig. 20). These results revealed that accessible chromatin regions are close to the transcription site (within an accessible chromatin region) with low DNA methylation. RNA-seq data further indicated that *eIF-2B subunit beta-like* gene is transcriptionally active across the examined tissues, including spike, root, and aerial organ, supporting its correct annotation (Fig. 4b, and Supplementary Data 4). Based on Whole-Genome Bisulfite Sequencing (WGBS), centromeric regions in Lo7 were further delineated, revealing seven CHG methylation dips within centromeric regions compared with the flanking sequences, potentially reflecting a conserved feature also reported in *Arabidopsis*^8^ (Supplementary Fig. 20b).

### Retrotransposon dynamics in rye centromeres

**Fig. 5:**
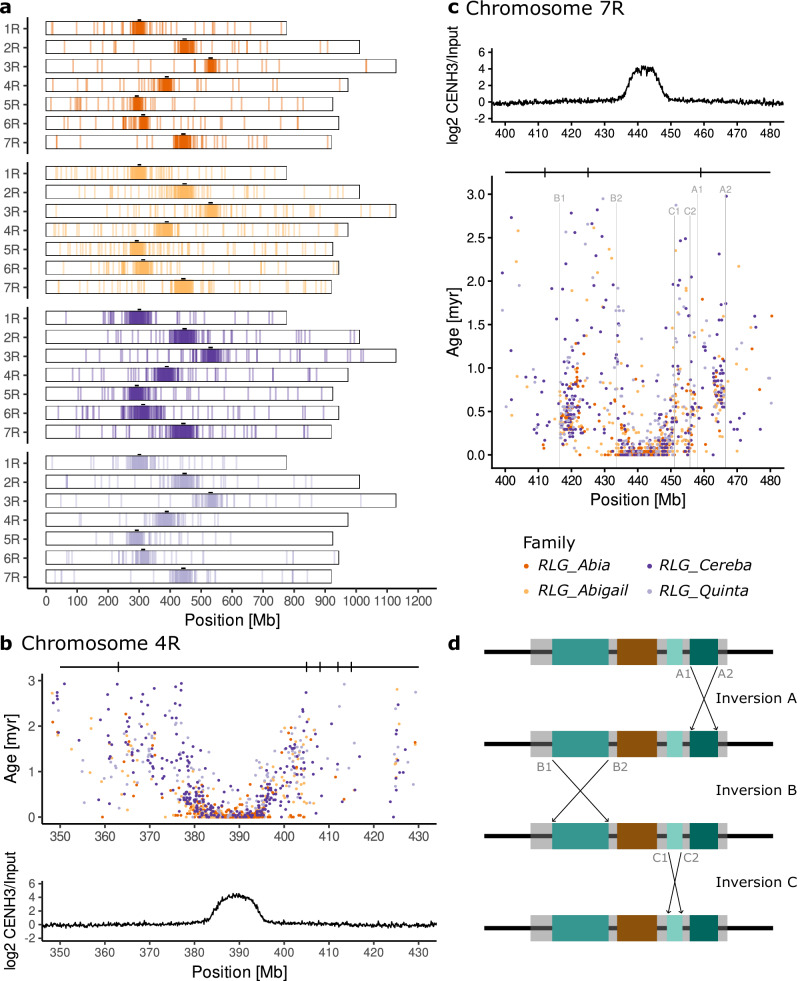
The retrotransposon families *RLG_Abia* (dark orange), *RLG_Abigail* (light orange), *RLG_Cereba* (dark purple) and *RLG_Quinta* (light purple) reveal putative inversions in the centromere region of chromosome 7R. **a** Positions of identified full-length copies across the genome. Black bars above the chromosome maps indicate centromere positions. **b** Distribution of the estimated ages (insertion times) of the full-length copies from these families across the pericentromeric region of chromosome 4R. Copies with an estimated age of 3 million years or younger are shown. The top track displays the assembly gaps found in the respective region per chromosome (top). The bottom track shows the corresponding CENH3 ChIP-seq data using a bin size of 100 kb. **c** CENH3 ChIP-seq data of the pericentromeric region of chromosome 7R (top, binsize 100 kb). Distribution of full-length copies’ estimated insertion ages across the displayed region. The gray dotted lines indicate the proposed breakpoints of inversions which are labeled with A-C corresponding to the inversions depicted in the model presented in **d**. Here, the functional centromere is indicated in brown while proposed inversions are depicted in turquoise.

Consistent with previous findings, we performed a comprehensive TE analysis on our Lo7_V3 genome assembly. Our results revealed that LTR retrotransposons (73.32%), particularly those belonging to the *Gypsy* superfamily (49.34%), are the predominant TEs in the genome (Supplementary Data 2). *Gypsy* elements exhibit a strong centromeric enrichment, suggesting their significant role in the structural organization and evolution of centromeric regions (Supplementary Fig. 21). In contrast, *Copia* elements (14.12%) were more broadly distributed across the chromosome arms (Supplementary Fig. 21). To reveal the evolution of centromere-specific retrotransposable elements in rye, we identified four families (*RLG*_*Cereba*, *RLG_Quinta*, *RLG*_*Abia and RLG_Abigail*) within the seven centromeres of Lo7 (Fig. 5a). We found *RLG_Cereba*, an element family well known to be present in the centromeres of the tribe *Triticeae*^5,24,38,39^, and its non-autonomous partner family *RLG*_*Quinta*^25^. In addition, we found *RLG_Abia*, which was previously found in wheat centromeres, but at very low abundance^18^, and its non-autonomous partner family, *RLG_Abigail*. Interestingly, a segment of *RLG_Abia* has been described in 2001 as a rye centromere-specific sequence and was used in FISH assays (pAWRC.1)^23,40^. The four TE families are very similar in sequence and sequence organization to their wheat homologs, with consensus sequences being between 77% and 94% identical across their entire lengths (Supplementary Fig. 22). Using a TE population analysis pipeline^41^, we identified 2098, 1298, 1602 and 2549 full-length elements of *RLG_Cereba*, *RLG_Quinta*, *RLG_Abia* and *RLG_Abigail*, respectively. The four TE families were highly enriched in centromeric and peri-centromeric regions (Fig. 5a). EDTA annotation of the identified functional centromere of all seven chromosomes revealed that approximately 93.1% to 97.8% of their sequences are accounted for by these four TE families (Supplementary Fig. 23).

Insertion ages of all retrotransposon copies were estimated based on divergence of their LTRs. We found the youngest copies of these families consistently to be located in the same regions where we identified the functional centromeres using CENH3 ChIP-seq analysis (Fig. 5, and Supplementary Figs. 24 and 25). This indicates that the centromeric retrotransposons actively target the functional centromere, similar to previous findings in wheat^24,25^. *RLG_Cereba* and *RLG_Abia* both encode a CR domain, with a conserved TRARAR/K motif, fused to their integrase (Supplementary Figs. 26 and 27). This motif was proposed to be essential for the centromere-specific integration of *RLG_Cereba* through direct binding of the CR domain of the integrase to CENH3^25^. As a consequence, the older copies are pushed away from the functional centromere over time. The example of the centromere of chromosome 4R (Fig. 5b) shows this gradual, passive movement outwards of older copies through insertions of new copies in the region of the functional centromere.

Ahmed et al. recently showed in a high-quality assembly for *Triticum monococcum* with completely assembled centromeres that the distribution of centromere-specific TE copies and their insertion ages can be used to identify centromere shifts and large centromeric inversions^24^. Interestingly, we found distribution patterns similar to those in *T. monococcum*, for example on chromosome 7R. These distribution patterns, specifically the distinct groups of TE insertions of similar age and at similar locations, will be referred to as “spatio-temporal TE clusters” hereafter. These patterns are different from the above-described passive outwards movement of older copies over time. Instead, they might suggest peri-/centromeric inversions. In the centromere of chromosome 7 R, we identified putative inversion breakpoints based on the physical locations and estimated insertion ages of TE copies (Fig. 5c). We therefore propose a three-step model to illustrate our hypothesis of this structural rearrangement in the peri-/centromeric region of 7R based on these spatio-temporal TE clusters. We propose that the observed structural differences might be the result of a series of inversions: The first putative inversion, here named Inversion A, might have occurred ~0.60 million years ago (mya) based on the estimated insertion age of the youngest elements in the spatio-temporal TE cluster at around 462.8–466.2 Mb, as reflected in locations and estimated insertion ages of TE is this region (Fig. 5c, and Supplementary Fig. 28a, b). A second putative inversion (Inversion B) might have taken place around 0.25 mya, bringing a segment of the previous centromere outwards to form the cluster at around 416.5–422 Mb. A third putative inversion of ~5 Mb (Inversion C) established the TE cluster at around 454.3 Mb to 455.8 Mb (Fig. 5d, and Supplementary Figs. 28–30).

We compared chromosome 7R of the phased genome assembly of Weining.V3 to Lo7 (Supplementary Fig. 28) and found inversion C not to be present in Weining.V3 H1. Thus, we could estimate the time frame of inversion C, as Weining.V3 H1 diverged from Lo7 ~70,000 years ago. Further, inversion C is present in Weining.V3 H2, which only diverged from Lo7 ~6300 years ago (Supplementary Figs. 28–30). The precise breakpoints in Lo7 were identified based on the homology to Weining.V3 H1 (Lo7 chr7R:451071203-455788938) and validated using ONT, PacBio and Illumina reads (Supplementary Figs. 28–30). The youngest TE cluster present from 454.3 Mb to 455.8 Mb in Lo7 is absent in Weining.V3 H1 (Supplementary Fig. 28), thus strengthening our hypothesis that the spatio-temporal TE clusters present in the (peri-) centromeric region of 7 R in Lo7 could be the result of inversions. Additionally, residual mapping of CENH3 ChIP-seq reads to the positions of these spatio-temporal TE clusters can be detected (Supplementary Fig. 20a). In the absence of haplotypes that do not contain the older putative inversions A and B, we inferred these two older inversion events in our model based on the location of the spatio-temporal TE clusters on chromosome 7R and the estimated insertion ages of these elements. However, we acknowledge that our hypothesis of structural rearrangements through multiple inversions must be further supported by additional comparisons to accessions not carrying these proposed inversions.

Finally, although less prominent, we also found breaks in the continuity of TE insertion sites and ages in other centromeres of Lo7, for example in chromosomes 2R, 3R and 6R (Supplementary Fig. 24), suggesting that these also underwent similar structural centromere dynamics in the past. However, the insertion site and age patterns are less clear and did not allow a reconstruction or dating of the events. A main difference to the centromeres of wheat is the high abundance of *RLG_Abia* and *RLG_Abigail* elements. In wheat, these two TE families appear to have been silent for a long time, since they were only found in relatively low numbers and their median insertion ages are between 1.2 and 2.5 million years (Supplementary Fig. 31). In contrast, the *RLG_Abia* and *RLG_Abigail* families appear to have been recently active in rye (and may still be), as their median insertion ages are ~0.4 million years (Supplementary Fig. 31). Furthermore, *RLG_Cereba*, *RLG_Quinta*, *RLG_Abia* and *RLG_Abigail* are all among the 10 highest expressed LTR retrotransposon families in rye while only *RLG_Cereba* and *RLG_Quinta* show high expression in wheat (Supplementary Fig. 31a, b). Additionally, we identified 63 *RLG_Abia* and 47 *RLG_Abigail* copies which are polymorphic in the predicted centromere region between Lo7 and Weining.V3 H2 (i.e., they have inserted after the two haplotypes diverged), while only very few new insertions were found outside the centromere (Supplementary Fig. 30). This indicates that centromeres, even between very closely related species, take different evolutionary trajectories and that different TE families may change their activity levels over relatively short evolutionary time periods.

### Sequence diversity of wheat/rye 1RS introgressions is low compared to the rye genepool

Recent wheat pan-genome studies have highlighted the crucial role of the rye 1BL/1RS translocation in modern wheat programs^2^. High-quality wheat genome assemblies have detailed syntenic analyzes of 1RS, facilitating comparisons between rye and multiple wheat accessions carrying this translocation. To investigate 1RS genetic diversity between rye and wheat, we analyzed ~300 Mb of 1RS/1BS sequences from multiple assemblies, including rye accessions Weining (V1^6^ and an improved version of V2^34^) and Lo7, as well as 1BL/1RS carrying wheat varieties Z8425B, HD6172, ZM16, ZM22, AMN, KF11, S4185, and Aikang58^2,34,42–44^. Syntenic analysis revealed limited 1RS diversity among wheat accessions (Supplementary Fig. 32). Collinearity analysis of 1RS suggested this homogeneity may result from the widespread use of early translocation lines in wheat breeding. In contrast, the two rye accessions showed substantially greater diversity in this region (Supplementary Table 12). Resistance genes such as *Pm8* and *Yr9* located on 1RS have been deployed in wheat breeding, providing strong disease resistance^4,45,46^. We further updated the annotation of resistance (R) genes containing NLR domains based on the whole-genome assembly of Lo7 (Lo7_V3), providing a clearer view of the genetic landscape of the 1RS chromosome arm (Supplementary Fig. 33). These observations underscore the potential value of introducing more diverse rye 1RS into wheat and triticale breeding programs to enhance genetic variation.

## Discussion

In this study, we present a highly improved chromosome-scale reference genome assembly for the rye inbred line Lo7, representing a major milestone in crop genomics. This work makes rye more accessible to genomic research and a valuable resource for crop improvement. The Lo7 genome assembly is near-complete, however not T2T, reflecting a significant advancement, especially given the challenges of rye’s high repeat content, structural variability, and large genome size. This achievement is especially relevant in the context of complex crop genomes, as evidenced by recent near-complete or T2T genome assemblies of wheat^16,17^. The integration of multiple sequencing technologies not only improves assembly quality but also paves the way for further genomic advances in rye^5,34^. Looking ahead, further advancements will focus on integrating more diverse genomic data sources, such as transcriptomics, to enhance genome annotations. Additionally, more comprehensive gene family analyzes, including those related to flowering and vernalization, will provide valuable insights into crop evolution, particularly in the context of wheat and other cereal crops.

Comparative analysis with Lo7_V2 underscores the critical role of high-fidelity long read data in revealing the true complexity of rye’s genomic architecture. In this study, about 5.2% of the genome was annotated as “repeat fragment”. We speculate that a substantial portion of these fragments may correspond to short tandem repeats (STRs), which were not fully reconstructed or classified by the repeat annotation tools due to their short length, high copy number, or structural variability. Specifically, the subtelomeric regions of 1RL and 6RS remain incomplete, and contig gaps are still present across the genome. These gaps, caused by the presence of repetitive sequences, have hindered the full assembly of subtelomeres or the majority of telomeres. To further assess the magnitude of potential missing content, we integrated an independent genome size estimate obtained from flow cytometry (7.06 Gb). In comparison, the assembled genome size of Lo7_V3 is 6.76 Gb, resulting in an estimated discrepancy of approximately 300 Mb, suggesting that a substantial proportion of the assembly-size discrepancy is attributable to unresolved or collapsed repeat-rich genomic regions. Additionally, current technologies such as optical genome mapping and Hi-C scaffolding remain insufficient for accurately resolving orientation errors in contigs located in subtelomeric regions, particularly those enriched with satellite sequences. These gaps could be addressed in the future by incorporating additional ultra-long ONT sequencing reads to improve genome continuity and completeness, facilitating the rye genome assembly to approach a real telomere-to-telomere (T2T) level by spanning highly repetitive satellite sequence regions and closing most of the remaining gaps. In this context, the highly repetitive supernumerary B chromosome of rye also offers a unique system to explore the accumulation and activity of transposable elements beyond the standard A chromosomes^47^.

We assembled the complete centromeric regions in rye and conducted an in-depth structural analysis. We identified centromere-located HC genes supported by expression data and estimated the size of functional (CENH3-enriched) centromere regions. In particular, we performed evolutionary analyzes within the *Gypsy* superfamily and identified families enriched in centromeric regions. Reassembly of recently published data for Weining_rye^34^, provided the opportunity to investigate some rye centromeres in greater detail. These centromeres are enriched in *Gypsy* retrotransposons, which correlate with CENH3 recruitment and unique DNA hypomethylation patterns, suggesting that a retrotransposon-mediated mechanism might be driving centromere evolution in cultivated and wild rye lineages. In contrast to wheat, the high numbers and recent insertion ages of elements of the *RLG_Abia* and *RLG_Abigail* families in Lo7 indicate a recent and potentially rye-specific amplification of these centromeric retrotransposons. Similar patterns have recently been reported in oat (*Avena sativa*)^48^ and other grass genomes, supporting the notion that centromeric retrotransposons, such as *RLG_Cereba* and *RLG_Abia* retrotransposons, have been coexisting and inserting in the centromeric niche over millions of years of grass genome evolution. Indeed, our comparisons of Lo7 with haplotypes H1 and H2 of rye cv. Weining revealed numerous polymorphic insertions of *RLG_Abia* (and its putative non-autonomous partner *RLG_Abigail*) which must have inserted in the past few thousand years, while we find no evidence of activity of the two families in wheat. Although the distinct distribution patterns of centromere-specific retrotransposons indicate a series of at least three inversions in the centromeric region of chromosome 7R within the last one million years, additional studies including further comparative analysis to other *S. cereale* genomes are needed to provide further support for our hypothesis. As a next step, we plan to conduct a rye pan-genome analysis by incorporating high-quality genome assemblies from diverse genotypes. This will enable us to further investigate the evolutionary dynamics of centromeric transposable elements and their contribution to genomic diversity.

Although we compared multiple 1RS of various rye and 1RS/1BL translocation lines, we found that the genetic diversity of the introgressed rye segment utilized in wheat remains narrow. This documents that the 1RS chromosomal fragments historically introduced into wheat breeding were derived from a very limited genetic pool, leaving much of rye’s genetic potential underexplored in wheat improvement^49^. Looking forward, the development of novel translocation lines carrying diverse rye chromosome segments may hold great promise for broadening the genetic base of wheat and triticale breeding and for delivering new insights into resistance improvement.

## Methods

### Plant materials and growth conditions

Plants were grown under controlled conditions in the greenhouse with a 16-h light/8-h dark photoperiod at 25 °C (day) and 18 °C (night). Humidity was maintained at 60%, and plants were irrigated with a nutrient solution weekly. Samples for DNA extraction were collected from young leaf tissues (2–3 weeks).

### Genome size estimation

Here we re-estimated the genome size of Lo7 using the following procedure. Nuclear suspensions were prepared by co-chopping ~0.5 cm² of fresh young leaf tissue together with *Pisum sativum* L. subsp. *sativum* convar. *sativum* var. *ponderosum* Alef. “Kifejtö Borsó” (Genebank accession number: PIS 630) as an internal reference standard in a Petri dish using the CyStain PI Absolute P reagent kit (Sysmex-Partec), according to the manufacturer’s instructions. The suspension was filtered through a 50 µm CellTrics mesh (Sysmex-Partec) and analyzed using a CytoFLEX flow cytometer (Beckman Coulter). Four individuals were analyzed, with four independent replicates per individual. The absolute nuclear DNA content (pg/2C) was calculated based on the values of the G1 peak means and the corresponding genome size (Mbp/1C), according to Dolezel et al.^50^. The DNA content of the reference genome was assumed to be 7.99 pg/2C^51^. The raw datasets are available in Supplementary Data 5.

### HMW DNA extraction, library preparation, PacBio HiFi and Oxford Nanopore sequencing

High molecular weight (HMW) DNA was extracted from a single Lo7 plant using the MACHEREY-NAGEL NucleoBond HMW DNA kit, following the manufacturer’s protocol. The extracted HMW DNA was quality-controlled for concentration using the Qubit 2.0® dsDNA HS assay (Invitrogen, Waltham, USA) and for fragment size distribution using the Agilent Femto Pulse System (Agilent Technologies, Santa Clara, USA).

For HiFi sequencing, the HMW DNA was fragmented into ~20 kb fragments using a Megaruptor 3 device (Diagenode) at speed setting 30. HiFi SMRTbell libraries were then prepared following the Pacific Biosciences SMRTbell Express Template Prep Kit 3.0 protocol. The final libraries were size-selected within a narrow 17–20 kb range using the SageELF system with a 0.75% Agarose Gel Cassette (Sage Science), following the manufacturer’s guidelines. HiFi circular consensus sequencing (CCS) reads were generated using the PacBio Revio platform (Pacific Biosciences) in accordance with the standard operating procedure.

For ONT reads, HMW DNA fragments were size-selected using the Short Read Eliminator (SRE) kit (Pacific Biosciences, Menlo Park, USA) with a 25 kb cut-off. Libraries were generated using the SQK-LSK114 Ligation Sequencing Kit V14 (Oxford Nanopore Technologies, Oxford, UK) following the manufacturer’s protocol with 2–3 µg size-selected HMW DNA as input. Sequencing was done on R10.4.1 PromethION (FLO-PRO114M) flow cells (Oxford Nanopore Technologies, Oxford, UK) with a run time of 72–96 h and raw data in the.pod5 format was acquired using MinKNOW (v.23.11 and v.24.02). Basecalling was performed with Dorado (v.0.9.1) using the dna_r10.4.1_e8.2_400bps_sup@v4.2.0 super-accurate basecalling model with the “--min-qscore 20” parameter to yield basecalled reads in the.bam format. Reads were then converted to the.fastq.gz format using Samtools^52^ and filtered for a minimum length of 25 kb using SeqKit^53^.

### Hi-C sequencing

For Hi-C sequencing, we followed a protocol similar to that used in a previous barley Hi-C study with slight modifications to optimize for rye^54^. Briefly, plant tissues were cross-linked using formaldehyde, and chromatin was fragmented by restriction enzyme (DpnII) digestion. The resulting DNA fragments were ligated with a biotinylated bridge linker, which allowed for the capture of ligation products that represent physically interacting chromatin regions. These biotinylated ligation products were then enriched through streptavidin beads and processed for paired-end sequencing. The Hi-C raw data were processed and used to generate a high-resolution contact map, revealing chromatin interactions and facilitating the construction of chromosome-scale scaffolds.

### Genome assembly and evaluation

PacBio HiFi reads were assembled using hifiasm (v.0.19.9) (parameters: -o -t –ul)^35^. Pseudomolecule construction was done with the TRITEX long-read assembly pipeline (https://tritexassembly.bitbucket.io/)^33^. Chimeric contigs and orientation errors were identified through manual inspection of Hi-C contact matrices. Genome completeness and consensus accuracy were evaluated using Merqury (v.1.3)^55^. Levels of duplication and heterozygosity were assessed with Merqury and GenomeScope 2.0^56^. Gene annotation was performed using the BUSCO (v.5.0.0) tool^57^, which was used to evaluate the completeness of gene models by assessing the presence of conserved core genes in the genome. Finally, contigs containing rye chloroplast sequences (NC_021761.1) and mitochondrial sequences (NC_086686.1) were removed from the unanchored regions, resulting in the final pseudomolecules. Additionally, we assessed the sequencing coverage and related metrics for both HiFi and ONT data, including average coverage, coefficient of variation (CV), and coverage uniformity. Sequencing reads were aligned to the reference genome using minimap2 (v.2.30)^58^, and per-base coverage was computed using SAMtools (v.1.22.1)^52^.

### Phased reassembly of the Weining rye genome

HiFi, ONT and Hi-C datasets were downloaded from the previous publication^34^. We integrated the following assembly pipeline to get the Weining.V3 haplotype 1 and Haplotype 2 contig groups. First, after performing quality control on both HiFi, Hi-C and ONT reads, the HiFi long reads were used to estimate the heterozygosity rate using GenomeScope 2.0^56^. The genome assembly followed a pipeline originally developed for potato, incorporating minor modifications^59^. HiFi, ONT and Hi-C reads were processed with hifiasm (v.0.24.0) (parameters: -o -t –ul –h1 –h2)^35^ to generate contig groups such as p_utg, ctg_Hap1, and ctg_Hap2. Using the TRITEX assembly pipeline (https://tritexassembly.bitbucket.io/)^33^, the two contig groups generated by the software were separately assembled. During this process, chimeric contigs were broken, ultimately yielding two preliminary draft pseudomolecules, Weining_Hap1 and Weining_Hap2. At the same time, we obtained information on contigs that were incorrectly partitioned in hifiasm for both assemblies. To correct the contig partitioning errors, contigs from the two above assemblies were combined and mapped again with Hi-C reads. Using YaHS^60^, we generated.hic file. Finally, the mis-partitioned contigs were corrected with the help of Juicebox (https://github.com/aidenlab/Juicebox/wiki)^61^ and with the assistance of HapHiC^62^, we obtained the haplotype-resolved assemblies along with the Hi-C contact maps. We also provide genome assembly statistics and TE annotations, as summarized in Supplementary Tables 8 and 9, and Supplementary Data 3.

### Optical genome mapping

Optical mapping of the rye genome was performed using the method of Simkova et al.^63^. In brief, a total of 1.8 million cell nuclei, purified from root tips of rye Lo7 seedlings by flow cytometry, were embedded in agarose miniplugs and treated by proteinase K. The resulting 548 ng of ultraHMW DNA were labeled at DLE-1 recognition sites (CTTAAG motive) and stained following the Bionano Prep Direct Label and Stain-G2 protocol (Bionano, San Diego, USA). The labelled molecules were analyzed on the Saphyr platform of Bionano. A total of 1500 Gbp single-molecule data greater than 150 kb, corresponding to 213× coverage of the rye Lo7 genome, was used to generate de novo optical genome map (OGM) by Bionano Solve (v.3.6.1_11162020), applying “optArguments_nonhaplotype_noES_noCut_DLE1_saphyr.xml” parameters (complete statistics in Supplementary Table 3). To validate the sequence assembly of the Lo7 genome, we aligned the OGM to the sequence in Access (v.1.7.1, Bionano). Identified mismatches between the OGM and the pseudomolecules are provided in Supplementary Data 6. OGM raw (single-molecule) data were used to quantify tandemly organized units of 45S rDNA, which comprise a single DLE-1 site, building a recognizable pattern. Molecules >150 kb were analyzed by the RefAligner function simpleRepeatStandalone included in the Bionano Solve package, applying repeat stretch tolerance of 0.1. Arrays containing at least six units of 8.8–9.3 kb, corresponding to the size of 45S rRNA genes, were considered (Supplementary Table 3).

### 5mCpG methylation

HiFi reads were aligned to the pseudomolecules using ccsmeth (v.0.5.0)^64^ with default parameters (align_hifi). Methylation calling was performed using pb-CpG-tools based on the resulting BAM files. All bigWig files were visualized in the Integrative Genomics Viewer (IGV_v.2.11.1)^65^.

### Gene model prediction

We performed de novo structural gene prediction, confidence classification, and functional annotation^66^. The strategy applied in this study only differs in the use of Helixer^67^, run with standard parameters, as an additional ab initio input source for Evidence Modeller (weight set to 10). High-confidence proteins had over 80% coverage and were complete, either with significant hits in UniMag or UniPoa but not PTREP. Low-confidence proteins were incomplete with hits in UniMag or UniPoa but not PTREP, or were complete without hits in any database. All RNA-seq and Iso-seq datasets can be accessed via Supplementary Note 1.6 of Rabanus-Wallace et al.^5^. The gene model liftover between Lo7_V2 and Lo7_V3 was provided in Supplementary Data 7 of the present study.

### Transposable element annotation using EDTA

To annotate transposable elements (TEs) in the genome, we used the Extensive De Novo TE Annotator EDTA (v.2.2.2)^68^ pipeline with a subset of 232 rye and wheat TE consensus sequences compiled from the curated library TREP (https://trep-db.uzh.ch/, subset available at https://github.com/Wicker-Lab/Rye_centromere_analysis), a comprehensive tool for identifying and classifying TEs. For the analysis of the centromeric regions, we ran EDTA (v.2.2.1)^68^ separately on the defined functional centromeres with a curated database (https://github.com/Wicker-Lab/Rye_centromere_analysis). First, we preprocessed the genome assembly by soft-masking low-complexity regions and simple repeats. Next, EDTA was run with default parameters to detect and classify TEs into different categories: long terminal repeat (LTR) retrotransposons, DNA transposons (TIR and Helitron), and other repeat elements. The proportion of the genome occupied by TEs was then calculated, and TEs were classified into super-families.

### Detection of TE insertion polymorphisms and divergence time estimates

To compare large chromosomal segments from Lo7 and Weining haplotypes H1 and H2 and identify TE presence/absence polymorphisms (PAPs), we used the original Perl scripts blast_compare_chromosome, TE_PAP_from_log_chr_comp and visual_chromosome_compare_segment, which identify collinear segments and identify TE PAPs. The scripts were also used to estimate divergence times between Lo7 and Weining haplotypes H1 and H2, respectively.

### Analysis of centromeric TEs

The identification and analysis of full-length transposable elements relied on the same pipeline reported in Wicker et al.^41^. Briefly, we first extracted ~100 LTRs per family (using consensus sequences from a subset of the TREP database https://trep-db.uzh.ch/, https://github.com/wicker314/TEpop), which we aligned using ClustalW with standard settings. If we identified distinct groups of LTR variants (likely representing TE subfamilies) in the alignment, these groups were separately re-aligned to produce consensus sequences for individual subfamilies. These consensus sequences were used in BLASTn searches against the genome. An identified element was classified as full-length if a pair of LTRs was found in the same orientation within a range of ±1000 bp of the length of the TE family consensus sequence and with no more than 5 bp missing at the LTR ends. Elements on the extrema of the size distribution were discarded to remove elements with large insertions or deletions. The resulting full-length elements were used for further analysis. For estimation of insertion ages, the full-length elements were aligned with the initial TE family consensus using the program Water (EMBOSS package obtained from ubuntu.com) with a gap extension penalty of 0.1 and a gap opening penalty of 50. The resulting pairwise comparisons were combined into a variant call format (vcf) file (https://github.com/wicker314/TEpop)^41^. We filtered for sequence variants with an allele frequency of more than 5%. Next, we used R for further analysis, data preparation and visualization as well as Inkscape for figure compiling. This workflow was used to identify full-length copies of rye (*S. cereale*) wheat (*T. aestivum*), Einkorn wheat (*T. monococcum*) and barley (*H. vulgare*).

### Construction of consensus sequences from centromeric retrotransposons

New consensus sequences for centromeric retrotransposons were constructed by randomly picking 20 TE copies with predicted insertion age zero (or as young as possible, if not available). Consensus sequences were constructed from alignments with Clustalw (obtained from Ubuntu repositories, ubuntu.com) at default settings (gap opening penalty of 10 and gap extension penalty of 0.1). Consensus sequences were used as a query in BLASTx searches against REXdb (Viridiplantae_v.4.0)^26^ to classify them into the corresponding clade. This confirmed that they all belonged to the CRM clade.

### Transcriptome analysis of centromeric retrotransposons

RNA-seq reads were mapped with the STAR software^69^ on a collection of 232 TE consensus sequences from rye and wheat deposited in the TREP database (https://trep-db.uzh.ch/), allowing for multi-mapping reads using the option --outFilterMultimapNmax 50 --outSAMmultNmax 1. Counts for mapped reads were produced with featureCounts^70^. For the analysis, only reads mapping in the correct transcriptional orientation were used. RNA-seq libraries for rye were obtained from the National Center for Biotechnology Information (NCBI) from bioprojects PRJEB35461 for rye (*S. cereale* Lo7) and PRJNA427159 for wheat (*T. aestivum* vc. Chinese Spring). Read counts were normalized for TE length and library size.

### Synteny analysis

For the analysis of synteny between genome sequences, we used plotsr^71^ to visualize and quantify synteny between different genome sequences. First, we performed pairwise sequence alignment using minimap2 (v.2.30)^58^ to align the genomic sequences of interest, such as rye Lo7_V3 and other related species. All variations were called using SyRI (v.1.5.4)^72^. The resulting.out files were then processed with plotsr (v.0.5.2)^71^ to generate syntenic plots, allowing us to examine the collinearity and structural conservation between genomes. The syntenic blocks were identified, and the degree of synteny was quantified. For the collinearity plot, we made the plot using R (v.4.2.2).

### Analyses of satellite sequences

To visualize the distribution of tandem repeats pSc200 (GenBank: Z54189.1), pSc250 (GenBank: Z50040.1) and pSC119.2 (GenBank: KF719093.1) on the assembly, the seven pseudomolecules were fragmented into 100-bp sequences and aligned to pSc200, pSc250 and pSC119.2 using Bowtie2 (v.2.5.0, default)^73^. The aligned reads were saved in separate files, and pSc200/250/119.2-positive reads were subsequently mapped back to the assembly using Bowtie2. The resulting BAM file was converted into a bigWig file with bamCoverage from deepTools (v.3.5.1)^74^ using a bin size of 10 kb. For 5S rDNA (GenBank: AY841027.1) and 45S rDNA (GenBank: KF482106.1), the relevant analysis was performed using the same methods as above. We also performed tandem repeat detection using TRF (v.4.0.9)^75^ and summarized the prominent monomers (pSat380, pSat571, and pSat118) corresponding to pSc200, pSc250, and pSc119.2 within the chromosome 1RS and unanchored regions (Supplementary Data 8 and 9).

### Fluorescence in situ hybridization (FISH)

Predicted probe annealing patterns were obtained using homology searches with 30,982 FISH probe sequences as queries and each rye genome assembly as a subject in turn (Lo7_V2 and Lo7_V3). The homology search and subsequent data handling and visualization were conducted directly from R using the BioDT package; The workflow is available at github.com/mtrw/Lo7v2_inSilicoFISH. To recreate the analysis presented here, scripts should be run in the order 1_run_blast.R, 2_count_gBins.R and 3a_plot_bins.R. In brief, BLAST (v.2.14.0) is called with preset parameters for short queries (argument ‘-task blastn-short’). Lo7_V3 assembly was divided into 1 Mb bins at 500 kb intervals. The sum of all bitscores of all probe sequence alignments overlapping each bin is calculated as an approximate indicator of the relative amount of probe binding that might be expected at that genomic region. Plots are constructed using R’s base plotting functions.

For the preparation of 5–10 kb long chromosome segment-specific barcoding FISH probes, non-overlapping, single-copy target-specific oligonucleotides were selected and synthesized as myTAGs® Labeled Libraries (Daicel Arbor Bioscience, Ann Arbor, MI, USA). The pooled oligos were labelled with either Atto 594 (red) or Alexa 488 (green). A dropping method^76^ was used to prepare mitotic metaphase spreads. FISH was performed as described earlier^77^ with minor alterations: 20 µL hybridization mixture per slide contained 50% deionized formamide, 25% 20× SSC, 1 mM Tris–HCL pH 8.0, 1 μL (400 ng) Atto 488 or Atto 594 labelled oligo chromosome painting probes, 1 μL (25 ng) Cy5-labelled oligo probes pSc200 and pSc250^78^, 10 μg/mL salmon sperm DNA, and 0.5 M EDTA. Hybridization mixture was denatured together with the chromosomal DNA on a hot plate at 80 °C for 2 min. Hybridization at 37 °C was performed for 20 h in a moist chamber. Subsequently, slides were washed in 2× SSC at room temperature for 1 min to remove coverslips, for 20 min at 58 °C and dehydrated in an ethanol series (70, 90 and 96%). Finally, the slides were air-dried and counterstained with 1 μg/mL 1 4′,6-diamidino-2-phenylindole (DAPI) in Vectashield (Vector Laboratories). Images were acquired with an epifluorescence microscope BX61 (Olympus) using a cooled charge-coupled device (CCD) camera (Orca ER, Hamamatsu). Pictures were processed and merged using Adobe Photoshop (Adobe Systems Incorporated, USA).

### Identification of centromeric regions

Centromeric regions were identified by integrating previously published CENH3 ChIP-seq data^22^. The CENH3 ChIP-seq data were first aligned to the rye genome using Bowtie2^73^. The resulting peaks were visualized on the genome using IGV^65^, enabling a clear representation of centromeric regions across chromosomes. To further refine the functional centromeric regions, we used deepTools (v.3.5.1)^74^ to generate a 10 kb resolution coverage plot, providing an overview of centromeric regions’ accessibility and distribution. Peaks corresponding to CENH3 binding were identified using MACS3 (v.3.0.0)^79^, which allows for the precise detection of enriched regions (only keep bins ≥300 kb length).

### ATAC sequencing

ATAC-seq was performed using either fresh (native) or crosslinked leaf tissues from 3-week-old seedlings. For the native sample, fresh leaves were finely chopped with a razor blade in nuclei isolation buffer (0.25 M sucrose, 10 mM Tris-HCl pH 8.0, 10 mM MgCl_2_, 1% Triton X-100, 5 mM β-mercaptoethanol) supplemented with 1x Halt™ Protease Inhibitor Cocktail (Thermo Scientific). The resulting slurry was filtered through a 50-µm cell strainer, and the nuclei were washed twice with the same buffer before being resuspended. For the native sample, an aliquot was analyzed by flow cytometry for quality control and quantification. Based on the quantification, approximately 75,000 nuclei were aliquoted, and the nuclei pellet was collected by centrifugation. For the crosslinked sample, fresh leaves were fixed under vacuum in 1% formaldehyde (Sigma-Aldrich 252549) for 20 min. Fixation was stopped by incubating the sample in 0.125 M glycine for 5 min. The tissues were frozen before nuclei isolation, following the same protocol as the native sample. After isolation, 75,000 nuclei were sorted by flow cytometry and incubated at 60 °C for 5 min. For both crosslinked (ATAC-seq1) and native (ATAC-seq2) samples, the nuclei pellet was resuspended in a transposition reaction mix containing Tagment DNA Enzyme (TDE1, Illumina, 20034197) and incubated at 37 °C for 30 min. Crosslinked samples (ATAC-seq1) were subsequently incubated 65 °C overnight in SDS buffer (50 mM Tris-HCl, pH 8.0, 1% SDS, 10 mM EDTA). Transposition products were purified using the MinElute PCR Purification Kit (QIAGEN, 28004). Libraries were then amplified with the NEBNext® High-Fidelity 2× PCR Master Mix (NEB, M0541), and further purified using VAHTSTM DNA Clean Beads (Vazyme, N411). The final libraries were sequenced in paired-end mode (2 × 151 cycles) on the Illumina NovaSeq 6000 (Illumina Inc., San Diego, CA, USA). Sequencing reads were adapter-trimmed using fastp (v.0.20.0)^80^ and aligned to the reference genome with BWA-MEM (v.0.7.17)^81^. SAMtools (v.1.16.1)^52^ was used to filter out duplicate and multi-mapping reads (-q 30), followed by peak calling (-q 0.01) with MACS3 (v.3.0.0)^79^. For visualization, BAM files containing uniquely mapped reads were converted to bigWig format using deepTools bamCoverage (v.3.5.1)^74^.

### Whole-genome bisulfite sequencing (WGBS)

DNA was extracted with DNeasy Plant Pro Kit (Qiagen), bisulfite-treated with the EZ DNA Methylation-Gold Kit (Zymo Research), and prepared for sequencing with the xGen Methyl-Seq Lib Prep Kit (IDT DNA), according to standard protocols from the manufacturers. Unmethylated Lambda DNA (Promega) was added as a spike-in to verify bisulfite conversion efficiency. Libraries were sequenced (paired-end, 2 × 151 cycles, Illumina NovaSeq X Plus, IPK Gatersleben) following protocols provided by the manufacturer (Illumina).

### Reporting summary

Further information on research design is available in the Nature Portfolio Reporting Summary linked to this article.

## Supplementary information

Supplementary Information

Peer Review file

Description of Additional Supplementary Files

Supplementary Data 1

Supplementary Data 2

Supplementary Data 3

Supplementary Data 4

Supplementary Data 5

Supplementary Data 6

Supplementary Data 7

Supplementary Data 8

Supplementary Data 9

Reporting Summary

## Source data

Source Data

## Data Availability

Genome assembly sequences, HiFi, ONT, Hi-C, ATAC and WGBS raw sequencing data have been deposited in the European Nucleotide Archive (ENA) under BioProject PRJEB91463^82^. The raw ONT sequencing data have been deposited in the European Nucleotide Archive (ENA) under BioProject PRJEB108142^82^. Gene annotation files and other raw datasets generated from the analyses have been deposited in Zenodo [10.5281/zenodo.15801361]^83^. Optical genome mapping cmap of Lo7 was deposited in Zenodo [10.5281/zenodo.17608037]^84^. Weining.V3 genome assembly sequences were deposited in Zenodo [10.5281/zenodo.19005175]^85^. The analyzed datasets and input files used for full-length transposable element analyses in Lo7 and Weining.V3 have been deposited in Zenodo [10.5281/zenodo.21357888]^86^.The Lo7_V3 gene annotation datasets are available via the Plant Genomics and Phenomics Research Data Repository [10.5447/ipk/2026/5]^87^. Source data are provided with this paper.

## Code availability

The scripts used to analyze full-length transposable elements are available at Github [https://github.com/Wicker-Lab/Rye_centromere_analysis]. Scripts used for WGBS and ATAC data analyses are available at Github [https://github.com/zihaozhu92/pepBAR]. The workflow for FISH probe design is available at Github [https://github.com/mtrw/Lo7v2_inSilicoFISH].

## References

[1] Martis, M. M. et al. Reticulate evolution of the rye genome. *Plant Cell* **25**, 3685–3698 (2013). 24104565 10.1105/tpc.113.114553 PMC3877785

[2] Jiao, C. et al. Pan-genome bridges wheat structural variations with habitat and breeding. *Nature* **637**, 384–393 (2025). 39604736 10.1038/s41586-024-08277-0

[3] Mago, R. et al. High-resolution mapping and mutation analysis separate the rust resistance genes *Sr31*, *Lr26* and *Yr9* on the short arm of rye chromosome 1. *Theor. Appl. Genet.* **112**, 41–50 (2005). 16283230 10.1007/s00122-005-0098-9

[4] Hurni, S. et al. Rye *Pm8* and wheat *Pm3* are orthologous genes and show evolutionary conservation of resistance function against powdery mildew. *Plant J.* **76**, 957–969 (2013). 24124925 10.1111/tpj.12345

[5] Rabanus-Wallace, M. T. et al. Chromosome-scale genome assembly provides insights into rye biology, evolution and agronomic potential. *Nat. Genet.* **53**, 564–573 (2021). 33737754 10.1038/s41588-021-00807-0 PMC8035072

[6] Li, G. et al. A high-quality genome assembly highlights rye genomic characteristics and agronomically important genes. *Nat. Genet.* **53**, 574–584 (2021). 33737755 10.1038/s41588-021-00808-z PMC8035075

[7] Liao, W. W. et al. A draft human pangenome reference. *Nature* **617**, 312–324 (2023). 37165242 10.1038/s41586-023-05896-x PMC10172123

[8] Naish, M. et al. The genetic and epigenetic landscape of the Arabidopsis centromeres. *Science* **374**, eabi7489 (2021). 34762468 10.1126/science.abi7489 PMC10164409

[9] Shang, L. et al. A complete assembly of the rice Nipponbare reference genome. *Mol. Plant* **16**, 1232–1236 (2023). 37553831 10.1016/j.molp.2023.08.003

[10] Chen, J. et al. A complete telomere-to-telomere assembly of the maize genome. *Nat. Genet.* **55**, 1221–1231 (2023). 37322109 10.1038/s41588-023-01419-6 PMC10335936

[11] Zhang, C. et al. The T2T genome assembly of soybean cultivar ZH13 and its epigenetic landscapes. *Mol. Plant* **16**, 1715–1718 (2023). 37803825 10.1016/j.molp.2023.10.003

[12] Li, W. et al. A gap-free complete genome assembly of oat and OatOmics, a multi-omics database. *Mol. Plant* **18**, 179–182 (2025). 39780493 10.1016/j.molp.2025.01.006

[13] Yan, H. et al. Post-polyploidization centromere evolution in cotton. *Nat. Genet.* **57**, 1021–1030 (2025). 40033059 10.1038/s41588-025-02115-3

[14] Huang, G. et al. A telomere-to-telomere cotton genome assembly reveals centromere evolution and a Mutator transposon-linked module regulating embryo development. *Nat. Genet.* **56**, 1953–1963 (2024). 39147922 10.1038/s41588-024-01877-6

[15] Chen, C. et al. A comprehensive omics resource and genetic tools for functional genomics research and genetic improvement of sorghum. *Mol. Plant* **18**, 703–719 (2025). 40055894 10.1016/j.molp.2025.03.005

[16] Tian, Y. et al. The near-complete genome assembly of pickling cucumber and its mutation library illuminate cucumber functional genomics and genetic improvement. *Mol. Plant* **18**, 551–554 (2025). 40045574 10.1016/j.molp.2025.03.001

[17] Liu, S. et al. A telomere-to-telomere genome assembly coupled with multi-omic data provides insights into the evolution of hexaploid bread wheat. *Nat. Genet.* **57**, 1008–1020 (2025). 40195562 10.1038/s41588-025-02137-x PMC11985340

[18] Wicker, T. et al. Impact of transposable elements on genome structure and evolution in bread wheat. *Genome Biol.* **19**, 103 (2018). 30115100 10.1186/s13059-018-1479-0 PMC6097303

[19] Gustafson, J. P., Dera, A. R. & Petrovic, S. Expression of modified rye ribosomal RNA genes in wheat. *Proc. Natl. Acad. Sci. USA* **85**, 3943–3945 (1988). 16593938 10.1073/pnas.85.11.3943 PMC280336

[20] Guo, J. et al. Frequent variations in tandem repeats pSc200 and pSc119.2 cause rapid chromosome evolution of open-pollinated rye. *Mol. Breed.* **39**, 133 (2019).

[21] Alkhimova, A. G., Heslop-Harrison, J. S., Shchapova, A. I. & Vershinin, A. V. Rye chromosome variability in wheat-rye addition and substitution lines. *Chromosome Res.* **7**, 205–212 (1999). 10421380 10.1023/a:1009299300018

[22] Liu, C. et al. Young retrotransposons and non-B DNA structures promote the establishment of dominant rye centromere in the 1RS.1BL fused centromere. *New Phytol.* **241**, 607–622 (2024). 37897058 10.1111/nph.19359

[23] Francki, M. G. Identification of *Bilby*, a diverged centromeric Ty1-*copia* retrotransposon family from cereal rye (*Secale cereale* L.). *Genome* **44**, 266–274 (2001). 11341737 10.1139/g00-112

[24] Ahmed, H. I. et al. Einkorn genomics sheds light on history of the oldest domesticated wheat. *Nature* **620**, 830–838 (2023). 37532937 10.1038/s41586-023-06389-7 PMC10447253

[25] Heuberger, M. et al. Evolution of Einkorn wheat centromeres is driven by the mutualistic interplay of two LTR retrotransposons. *Mob. DNA* **15**, 16 (2024). 39103880 10.1186/s13100-024-00326-9 PMC11302176

[26] Neumann, P., Novak, P., Hostakova, N. & Macas, J. Systematic survey of plant LTR-retrotransposons elucidates phylogenetic relationships of their polyprotein domains and provides a reference for element classification. *Mob. DNA* **10**, 1 (2019). 30622655 10.1186/s13100-018-0144-1 PMC6317226

[27] Neumann, P., Yan, H. & Jiang, J. The centromeric retrotransposons of rice are transcribed and differentially processed by RNA interference. *Genetics* **176**, 749–761 (2007). 17409063 10.1534/genetics.107.071902 PMC1894605

[28] Sharma, A. & Presting, G. G. Centromeric retrotransposon lineages predate the maize/rice divergence and differ in abundance and activity. *Mol. Genet. Genom.* **279**, 133–147 (2008). 10.1007/s00438-007-0302-5 18000683

[29] Gao, X., Hou, Y., Ebina, H., Levin, H. L. & Voytas, D. F. Chromodomains direct integration of retrotransposons to heterochromatin. *Genome Res.* **18**, 359–369 (2008). 18256242 10.1101/gr.7146408 PMC2259100

[30] Neumann, P. et al. Plant centromeric retrotransposons: a structural and cytogenetic perspective. *Mob. DNA* **2**, 4 (2011). 21371312 10.1186/1759-8753-2-4 PMC3059260

[31] Tsukahara, S. et al. Centrophilic retrotransposon integration via CENH3 chromatin in Arabidopsis. *Nature* **637**, 744–748 (2025). 39743586 10.1038/s41586-024-08319-7 PMC11735389

[32] Bauer, E. et al. Towards a whole-genome sequence for rye (*Secale cereale* L.). *Plant J.* **89**, 853–869 (2017). 27888547 10.1111/tpj.13436

[33] Marone, M. P., Singh, H. C., Pozniak, C. J. & Mascher, M. A technical guide to TRITEX, a computational pipeline for chromosome-scale sequence assembly of plant genomes. *Plant Methods* **18**, 128 (2022). 36461065 10.1186/s13007-022-00964-1 PMC9719158

[34] Yi, C. et al. High-resolution genome assembly reveals retrotransposon-mediated centromere dynamics in rye. *Genome Biol.* **26**, 308 (2025). 41013584 10.1186/s13059-025-03792-3 PMC12465393

[35] Cheng, H., Asri, M., Lucas, J., Koren, S. & Li, H. Scalable telomere-to-telomere assembly for diploid and polyploid genomes with double graph. *Nat. Methods* **21**, 967–970 (2024). 38730258 10.1038/s41592-024-02269-8 PMC11214949

[36] Bousios, A., Kakutani, T. & Henderson, I. R. Centrophilic retrotransposons of plant genomes. *Annu. Rev. Plant Biol.* **76**, 579–604 (2025). 39952673 10.1146/annurev-arplant-083123-082220

[37] Liu, C. et al. Unveiling the distinctive traits of functional rye centromeres: minisatellites, retrotransposons, and R-loop formation. *Sci. China Life Sci.* **67**, 1989–2002 (2024). 38805064 10.1007/s11427-023-2524-0

[38] Mascher, M. et al. A chromosome conformation capture ordered sequence of the barley genome. *Nature* **544**, 427–433 (2017). 28447635 10.1038/nature22043

[39] Wicker, T. et al. The repetitive landscape of the 5100 Mbp barley genome. *Mob. DNA* **8**, 22 (2017). 29270235 10.1186/s13100-017-0102-3 PMC5738225

[40] Francki, M. G., Berzonsky, W. A., Ohm, H. W. & Anderson, J. M. Physical location of a HSP70 gene homologue on the centromere of chromosome 1B of wheat (*Triticum aestivum* L.). *Theor. Appl. Genet.* **104**, 184–191 (2002). 12582685 10.1007/s00122-001-0781-4

[41] Wicker, T. et al. Transposable element populations shed light on the evolutionary history of wheat and the complex co-evolution of autonomous and non-autonomous retrotransposons. *Adv. Genet.* **3**, 2100022 (2022). 36619351 10.1002/ggn2.202100022 PMC9744471

[42] Li, G. et al. Genomic analysis of Zhou8425B, a key founder parent, reveals its genetic contributions to elite agronomic traits in wheat breeding. *Plant Commun.* **6**, 101222 (2025). 39690740 10.1016/j.xplc.2024.101222 PMC11956103

[43] Wang, Z. et al. Near-complete assembly and comprehensive annotation of the wheat Chinese Spring genome. *Mol. Plant* **18**, 892–907 (2025). 39949061 10.1016/j.molp.2025.02.002

[44] Jia, J. et al. Genome resources for the elite bread wheat cultivar Aikang 58 and mining of elite homeologous haplotypes for accelerating wheat improvement. *Mol. Plant* **16**, 1893–1910 (2023). 37897037 10.1016/j.molp.2023.10.015

[45] Wang, C. et al. The *Yr9* gene encoding a CC-NBS-LRR protein in the 1RS-1BL translocation confers wheat stripe rust resistance. *Sci. China Life Sci.* **68**, 2804–2806 (2025). 40285910 10.1007/s11427-025-2929-6

[46] Yu, Y. et al. Wheat stripe rust resistance gene *Yr9*, derived from rye, is a CC-NBS-LRR gene in a highly conserved NLR cluster. *Sci. China Life Sci.* **68**, 2807–2809 (2025). 40285909 10.1007/s11427-024-2932-5

[47] Chen, J. et al. The genetic mechanism of B chromosome drive in rye illuminated by chromosome-scale assembly. *Nat. Commun.* **15**, 9686 (2024). 39516474 10.1038/s41467-024-53799-w PMC11549084

[48] Wehrkamp, C. M., Heuberger, M. & Wicker, T. Transposable element dynamics drive rapid evolution of centromere architecture in the *Avena* genus. *Genome Biol.* **27**, 200 (2026). 42304491 10.1186/s13059-026-04127-6 PMC13270711

[49] Schlegel, R. & Korzun, V. About the origin of 1RS.1BL wheat-rye chromosome translocations from Germany. *Plant Breed.* **116**, 537–540 (1997).

[50] Dolezel, J., Bartos, J., Voglmayr, H. & Greilhuber, J. Letter to the editor. *Cytom. A* **51**, 127–128 (2003). 10.1002/cyto.a.10013 12541287

[51] Soni, A. & Henry, R. J. Re-calibration of flow cytometry standards for plant genome size estimation. *Front. Plant. Sci.* **16**, 1548766 (2025). 41158695 10.3389/fpls.2025.1548766 PMC12555019

[52] Danecek, P. et al. Twelve years of SAMtools and BCFtools. *Gigascience* **10**, giab008 (2021). 33590861 10.1093/gigascience/giab008 PMC7931819

[53] Shen, W., Sipos, B. & Zhao, L. SeqKit2: A Swiss army knife for sequence and alignment processing. *Imeta* **3**, e191 (2024). 38898985 10.1002/imt2.191 PMC11183193

[54] Jayakodi, M. et al. The barley pan-genome reveals the hidden legacy of mutation breeding. *Nature* **588**, 284–289 (2020). 33239781 10.1038/s41586-020-2947-8 PMC7759462

[55] Rhie, A., Walenz, B. P., Koren, S. & Phillippy, A. M. Merqury: reference-free quality, completeness, and phasing assessment for genome assemblies. *Genome Biol.* **21**, 245 (2020). 32928274 10.1186/s13059-020-02134-9 PMC7488777

[56] Ranallo-Benavidez, T. R., Jaron, K. S. & Schatz, M. C. GenomeScope 2.0 and Smudgeplot for reference-free profiling of polyploid genomes. *Nat. Commun.* **11**, 1432 (2020). 32188846 10.1038/s41467-020-14998-3 PMC7080791

[57] Manni, M., Berkeley, M. R., Seppey, M. & Zdobnov, E. M. BUSCO: Assessing genomic data quality and beyond. *Curr. Protoc.* **1**, e323 (2021). 34936221 10.1002/cpz1.323

[58] Li, H. Minimap2: pairwise alignment for nucleotide sequences. *Bioinformatics* **34**, 3094–3100 (2018). 29750242 10.1093/bioinformatics/bty191 PMC6137996

[59] Cheng, L. et al. Leveraging a phased pangenome for haplotype design of hybrid potato. *Nature* **640**, 408–417 (2025). 39843749 10.1038/s41586-024-08476-9 PMC11981936

[60] Zhou, C., McCarthy, S. A. & Durbin, R. YaHS: yet another Hi-C scaffolding tool. *Bioinformatics* **39**, btac808 (2023). 36525368 10.1093/bioinformatics/btac808 PMC9848053

[61] Durand, N. C. et al. Juicebox provides a visualization system for Hi-C contact maps with unlimited zoom. *Cell Syst.* **3**, 99–101 (2016). 27467250 10.1016/j.cels.2015.07.012 PMC5596920

[62] Zeng, X. et al. Chromosome-level scaffolding of haplotype-resolved assemblies using Hi-C data without reference genomes. *Nat. Plants* **10**, 1184–1200 (2024). 39103456 10.1038/s41477-024-01755-3

[63] Simkova, H., Tulpova, Z. & Capal, P. Flow sorting-assisted optical mapping. *Methods Mol. Biol.* **2672**, 465–483 (2023). 37335494 10.1007/978-1-0716-3226-0_28

[64] Tse, O. Y. O. et al. Genome-wide detection of cytosine methylation by single molecule real-time sequencing. *Proc. Natl. Acad. Sci. USA* **118**, e2019768118 10.1073/pnas.2019768118 (2021). 10.1073/pnas.2019768118 PMC7865158 33495335

[65] Robinson, J. T. et al. Integrative genomics viewer. *Nat. Biotechnol.* **29**, 24–26 (2011). 21221095 10.1038/nbt.1754 PMC3346182

[66] Mascher, M. et al. Long-read sequence assembly: a technical evaluation in barley. *Plant Cell* **33**, 1888–1906 (2021). 33710295 10.1093/plcell/koab077 PMC8290290

[67] Stiehler, F. et al. Helixer: cross-species gene annotation of large eukaryotic genomes using deep learning. *Bioinformatics* **36**, 5291–5298 (2021). 33325516 10.1093/bioinformatics/btaa1044 PMC8016489

[68] Ou, S. et al. Benchmarking transposable element annotation methods for creation of a streamlined, comprehensive pipeline. *Genome Biol.* **20**, 275 (2019). 31843001 10.1186/s13059-019-1905-y PMC6913007

[69] Dobin, A. & Gingeras, T. R. Mapping RNA-seq reads with STAR. *Curr. Protoc. Bioinform.* **51**, 11.14.11–11.14.19 (2015). 10.1002/0471250953.bi1114s51 PMC4631051 26334920

[70] Liao, Y., Smyth, G. K. & Shi, W. featureCounts: an efficient general purpose program for assigning sequence reads to genomic features. *Bioinformatics* **30**, 923–930 (2014). 24227677 10.1093/bioinformatics/btt656

[71] Goel, M. & Schneeberger, K. plotsr: visualizing structural similarities and rearrangements between multiple genomes. *Bioinformatics* **38**, 2922–2926 (2022). 35561173 10.1093/bioinformatics/btac196 PMC9113368

[72] Goel, M., Sun, H., Jiao, W. B. & Schneeberger, K. SyRI: finding genomic rearrangements and local sequence differences from whole-genome assemblies. *Genome Biol.* **20**, 277 (2019). 31842948 10.1186/s13059-019-1911-0 PMC6913012

[73] Langmead, B. & Salzberg, S. L. Fast gapped-read alignment with Bowtie 2. *Nat. Methods* **9**, 357–359 (2012). 22388286 10.1038/nmeth.1923 PMC3322381

[74] Ramirez, F. et al. deepTools2: a next generation web server for deep-sequencing data analysis. *Nucleic Acids Res.* **44**, W160–W165 (2016). 27079975 10.1093/nar/gkw257 PMC4987876

[75] Benson, G. Tandem repeats finder: a program to analyze DNA sequences. *Nucleic Acids Res.* **27**, 573–580 (1999). 9862982 10.1093/nar/27.2.573 PMC148217

[76] Aliyeva-Schnorr, L., Ma, L. & Houben, A. A Fast Air-dry dropping chromosome preparation method suitable for FISH in plants. *J. Vis. Exp*. e53470 10.3791/53470 (2015). 10.3791/53470 PMC4694022 26709593

[77] Aliyeva-Schnorr, L. et al. Cytogenetic mapping with centromeric bacterial artificial chromosomes contigs shows that this recombination-poor region comprises more than half of barley chromosome 3H. *Plant J.* **84**, 385–394 (2015). 26332657 10.1111/tpj.13006

[78] Fu, S. et al. Oligonucleotide probes for ND-FISH analysis to identify rye and wheat chromosomes. *Sci. Rep.* **5**, 10552 (2015). 25994088 10.1038/srep10552 PMC4440213

[79] Zhang, Y. et al. Model-based analysis of ChIP-Seq (MACS). *Genome Biol.* **9**, R137 (2008). 18798982 10.1186/gb-2008-9-9-r137 PMC2592715

[80] Chen, S., Zhou, Y., Chen, Y. & Gu, J. fastp: an ultra-fast all-in-one FASTQ preprocessor. *Bioinformatics* **34**, i884–i890 (2018). 30423086 10.1093/bioinformatics/bty560 PMC6129281

[81] Vasimuddin, M., Misra, S., Li, H. & Aluru, S. Efficient Architecture-Aware Acceleration of BWA-MEM for Multicore Systems. In *2019 IEEE International Parallel and Distributed Processing Symposium (IPDPS).* pp. 314–324 (IEEE, Rio de Janeiro, Brazil, 2019).

[82] Burgin, J. et al. The European Nucleotide Archive in 2022. *Nucleic Acids Res.* **51**, D121–D125 (2023). 36399492 10.1093/nar/gkac1051 PMC9825583

[83] Chen, E. Unveiling centromeric retrotransposon dynamics through a near-complete rye genome assembly. Zenodo 10.5281/zenodo.15801361 (2025).

[84] Chen, E. et al. Optical genome mapping cmap of rye Lo7. Zenodo. 10.5281/zenodo.17608037.85 (2025).

[85] Chen, E. & Nils, S. Rye Weining.V3 H1&H2. Zenodo. 10.5281/zenodo.19005175 (2026).

[86] Wehrkamp, C. M. & Wicker, T. Data for publication: Unveiling centromeric retrotransposon dynamics through a high quality rye genome assembly. Zenodo. 10.5281/zenodo.21357888 (2026).10.1038/s41467-026-76753-442603802

[87] Arend, D. et al. PGP repository: a plant phenomics and genomics data publication infrastructure. *Database* **2016**, baw033 (2016). 27087305 10.1093/database/baw033 PMC4834206

