# Supplementary material for: Unveiling centromeric retrotransposon dynamics through a high-quality rye genome assembly
Source: Nat Commun. 2026 Aug 15;17:8413. doi: 10.1038/s41467-026-76753-4 (PMC13477480; doi:10.1038/s41467-026-76753-4)
Supplement: Supplementary file 1 [file 41467_2026_76753_MOESM13_ESM.pdf]

nature portfolio

Corresponding author(s): Nils Stein

Last updated by author(s): Jul 17, 2026

Reporting Summary

Nature Portfolio wishes to improve the reproducibility of the work that we publish. This form provides structure for consistency and transparency in reporting. For further information on Nature Portfolio policies, see our Editorial Policies and the Editorial Policy Checklist.

Statistics

For all statistical analyses, confirm that the following items are present in the figure legend, table legend, main text, or Methods section.

n/a	Confirmed	
☐	☒	The exact sample size (*n*) for each experimental group/condition, given as a discrete number and unit of measurement
☐	☒	A statement on whether measurements were taken from distinct samples or whether the same sample was measured repeatedly
☒	☐	The statistical test(s) used AND whether they are one- or two-sided<br>*Only common tests should be described solely by name; describe more complex techniques in the Methods section.*
☒	☐	A description of all covariates tested
☒	☐	A description of any assumptions or corrections, such as tests of normality and adjustment for multiple comparisons
☐	☒	A full description of the statistical parameters including central tendency (e.g. means) or other basic estimates (e.g. regression coefficient) AND variation (e.g. standard deviation) or associated estimates of uncertainty (e.g. confidence intervals)
☒	☐	For null hypothesis testing, the test statistic (e.g. *F*, *t*, *r*) with confidence intervals, effect sizes, degrees of freedom and *P* value noted<br>*Give P values as exact values whenever suitable.*
☒	☐	For Bayesian analysis, information on the choice of priors and Markov chain Monte Carlo settings
☒	☐	For hierarchical and complex designs, identification of the appropriate level for tests and full reporting of outcomes
☒	☐	Estimates of effect sizes (e.g. Cohen's *d*, Pearson's *r*), indicating how they were calculated

Our web collection on statistics for biologists contains articles on many of the points above.

Software and code

Policy information about availability of computer code

Data collection

HiFi sequencing data were generated using our in-house long-read sequencing platform, PacBio Revio (Pacific Biosciences). ONT reads were produced with the in-house R10.4.1 PromethION platform (FLO-PRO114M). ATAC-seq data were generated using the short-read Illumina NovaSeq 6000 platform. CENH3 ChIP-seq data for rye Lo7 were directly downloaded from the GSA (Genome Sequence Archive; https://bigd.big.ac.cn/gsa/) under accession numbers CRA011915 and CRA011912 (data are publicly accessible).Hi-C sequencing data were generated following a protocol similar to that used in previous barley Hi-C studies and were sequenced on the Illumina NovaSeq 6000 platform.Bionano optical mapping data were generated using the Bionano Genomics Saphyr platform.Gene model prediction in this study differs from previous approaches only by the additional use of Helixer, run with standard parameters, as an ab initio input source for Evidence Modeler (weight set to 10).Access to all RNA-seq and Iso-Seq datasets is described in a previous publication (Rabanus-Wallace, M. T. et al. Chromosome-scale genome assembly provides insights into rye biology, evolution and agronomic potential. Nat Genet 53, 564–573, doi:10.1038/s41588-021-00807-0 (2021)).For FISH experiments, chromosome segment-specific barcoding probes (5–10 kb) were designed to target non-overlapping, single-copy regions. These oligonucleotides were synthesized as myTAGs® Labeled Libraries (Daicel Arbor Biosciences, Ann Arbor, MI, USA), and labeled with either Atto 594 (red) or Alexa 488 (green). Microscopy images were captured using an Olympus BX61 fluorescence microscope equipped with a cooled CCD camera. Images were processed and merged using Adobe Photoshop (Adobe Systems Incorporated, USA, http://www.adobe.com).

Data analysis

Genome assembly was performed using hifiasm (v.0.19.9) with default parameters (-o, -t48, --ul) or hifiasm (v.0.24.0) (parameters: -o -t –ul --h1 –h2) to integrate both HiFi and ONT long-read data. HiFi circular consensus sequencing (CCS) reads were generated using the PacBio Revio platform (Pacific Biosciences, https://github.com/pacificbiosciences) following the standard operating procedure. Raw ONT data in .pod5 format were obtained using MinKNOW (v.23.11 and v.24.02). Basecalling was performed with Dorado (v.0.9.1) using the dna_r10.4.1_e8.2_400bps_sup@v.4.2.0 super-accurate model and the --min-qscore 20 parameter, resulting in basecalled reads in .bam format. De novo assembly of the optical genome map (OGM) was conducted using Bionano Solve software (v.3.6.1_11162020) with

parameters: optArguments_nonhaplotype_noES_noCut_DLE1_saphyr.xml.Contig scaffolding was performed using the TRITEX long-read assembly pipeline (https://tritexassembly.bitbucket.io/).Genome completeness and consensus accuracy were evaluated using Merqury (v.1.3).Duplication and heterozygosity levels were assessed using Merqury(v.1.3) and GenomeScope 2.0. Gene annotation quality was evaluated using BUSCO (v.5.0.0).HiFi reads were aligned to pseudomolecules using ccsmeth, and methylation calling was performed using pb-CpG-tools based on the resulting BAM files.Transposable element (TE) annotation was conducted using EDTA (v.2.2.2), incorporating the curated TREP library (https://trep-db.uzh.ch/). Synteny visualization and quantification were carried out using plotsr(v.0.5.2). The resulting .bw files generated from the above datasets (pSc200, pSc250, and CENH3 ChIP-seq for Lo7_V2 and Lo7_V3) were used for visualization with pyGenomeTracks (Galaxy Version 3.8+galaxy2). RNA-seq reads were aligned using STAR (v.2.7.9a), and the resulting sorted BAM files were visualized using IGV.NLR gene (R gene) analysis was conducted using NLR-Annotator (https://github.com/steuernb/NLR-Annotator), quantifying gene counts in non-overlapping 1 Mb genomic bins across each chromosome.
Other softwares were used in this paper, including Bowtie2(v.2.3.5.1), Integrative Genomics Viewer (IGV), deepTools(v.3.5.1), blast(v.2.14.0), MACS3(v.3.0.0), SAMtools (v.1.16.1), minimap2(v.2.24), seqkit(v.0.9.1), fastp (v.0.20.0), seqtk(v.1.3-r116), jdk(v.21.0.6), jellyfish(v.2.2.6), SyRI (v.1.6), bedtools(v.2.31.1), bwa(v.0.7.17), cutadapt(v.3.3), EMBOSS(v.6.6.0), LTR_retriever(v.3.0.2), RepeatMasker(v.4.1.5), tidk(v.0.2.63), trf(v.4.0.9).
All data visualizations were generated under R(v.4.4.1 or v.3.5.1) and python(v.3.12.0).
The scripts used to analyze full-length transposable elements are available at https://github.com/Wicker-Lab/Rye_centromere_analysis. Scripts used for WGBS and ATAC data analyses are available at https://github.com/zihaozhu92/pepBAR. The workflow for FISH probe design is available at github.com/mtrw/Lo7v2_inSilicoFISH.

For manuscripts utilizing custom algorithms or software that are central to the research but not yet described in published literature, software must be made available to editors and reviewers. We strongly encourage code deposition in a community repository (e.g. GitHub). See the Nature Portfolio guidelines for submitting code & software for further information.

Data

Policy information about availability of data

All manuscripts must include a data availability statement. This statement should provide the following information, where applicable:

- Accession codes, unique identifiers, or web links for publicly available datasets
- A description of any restrictions on data availability
- For clinical datasets or third party data, please ensure that the statement adheres to our policy

Genome assembly sequences, HiFi_CCS, ONT, Hi-C, ATAC and WGBS raw sequencing data have been deposited in the European Nucleotide Archive (ENA) under BioProjectproject ID PRJEB91463 [https://www.ebi.ac.uk/ena/browser/text-search?query=PRJEB91463]. The raw ONT sequencing data have been deposited in the European Nucleotide Archive (ENA) under BioProject PRJEB108142 [https://www.ebi.ac.uk/ena/browser/text-search?query=PRJEB108142%20].
The datasets as follows were deposited in Zenodo, including the Lo7_V3 genome assembly, assembly AGP file, genome annotation (both high-confidence and low-confidence gene models), 5mCpG methylation .bw data, CENH3 ChIP-seq data (Lo7_V2 and Lo7_V3) .bw data, transposable element (TE) annotations, ATAC-seq .bw data, short tandem repeats (pSc200 and pSc250) annotation and nucleotide-binding leucine-rich repeat (NLR) gene annotations. Chen, E. (2025). Unveiling centromeric retrotransposon dynamics through a near-complete rye genome assembly. Zenodo. https://doi.org/10.5281/zenodo.15801361. Optical genome mapping cmap of Lo7 was deposited in Zenodo, https://doi.org/10.5281/zenodo.17608037. For Weining.V3 genome assembly sequences were deposited in Zenodo, https://doi.org/10.5281/zenodo.19005175. The analyzed datasets and input files used for full-length transposable element analyses in Lo7 and Weining.V3 have been deposited in Zenodo [https://doi.org/10.5281/zenodo.21357888].

Research involving human participants, their data, or biological material

Policy information about studies with human participants or human data. See also policy information about sex, gender (identity/presentation), and sexual orientation and race, ethnicity and racism.

Reporting on sex and gender	n/a
Reporting on race, ethnicity, or other socially relevant groupings	n/a
Population characteristics	n/a
Recruitment	n/a
Ethics oversight	n/a

Note that full information on the approval of the study protocol must also be provided in the manuscript.

Field-specific reporting

Please select the one below that is the best fit for your research. If you are not sure, read the appropriate sections before making your selection.

☒ Life sciences ☐ Behavioural & social sciences ☐ Ecological, evolutionary & environmental sciences

For a reference copy of the document with all sections, see nature.com/documents/nr-reporting-summary-flat.pdf

Life sciences study design

All studies must disclose on these points even when the disclosure is negative.

Sample size	For HiFi, Hi-C, and ONT sequencing, we randomly selected a single plant for high-molecular-weight DNA extraction and library preparation. And the sample size was determined based on our experience from previous studies and the requirements of the respective protocols.
Data exclusions	No data was excluded from the analysis in the article.
Replication	The number of replication are indicated from the section Methods and Supplementary Figures and Tables.
Randomization	To avoid elevated heterozygosity resulting from pooling multiple plants, we randomly selected a single rye plant for sequencing and library preparation.
Blinding	Blinding was not applied because the study involved genomic data generation and computational analyses rather than subjective experimental assessments.

Reporting for specific materials, systems and methods

We require information from authors about some types of materials, experimental systems and methods used in many studies. Here, indicate whether each material, system or method listed is relevant to your study. If you are not sure if a list item applies to your research, read the appropriate section before selecting a response.

Materials & experimental systems

n/a	Involved in the study	
☒	☐	Antibodies
☒	☐	Eukaryotic cell lines
☒	☐	Palaeontology and archaeology
☒	☐	Animals and other organisms
☒	☐	Clinical data
☒	☐	Dual use research of concern
☐	☒	Plants

Methods

n/a	Involved in the study	
☒	☐	ChIP-seq
☐	☒	Flow cytometry
☒	☐	MRI-based neuroimaging

Dual use research of concern

Policy information about dual use research of concern

Hazards

Could the accidental, deliberate or reckless misuse of agents or technologies generated in the work, or the application of information presented in the manuscript, pose a threat to:

No	Yes	
☒	☐	Public health
☒	☐	National security
☒	☐	Crops and/or livestock
☒	☐	Ecosystems
☒	☐	Any other significant area

Experiments of concern

Does the work involve any of these experiments of concern:

No	Yes	
☒	☐	Demonstrate how to render a vaccine ineffective
☒	☐	Confer resistance to therapeutically useful antibiotics or antiviral agents
☒	☐	Enhance the virulence of a pathogen or render a nonpathogen virulent
☒	☐	Increase transmissibility of a pathogen
☒	☐	Alter the host range of a pathogen
☒	☐	Enable evasion of diagnostic/detection modalities
☒	☐	Enable the weaponization of a biological agent or toxin
☒	☐	Any other potentially harmful combination of experiments and agents

Plants

Seed stocks	The rye inbred variety Lo7 seeds were provided by Dr. Andres Gordillo from KWS.
Novel plant genotypes	n/a
Authentication	n/a

Flow Cytometry

Plots

Confirm that:

☐ The axis labels state the marker and fluorochrome used (e.g. CD4-FITC).

☐ The axis scales are clearly visible. Include numbers along axes only for bottom left plot of group (a 'group' is an analysis of identical markers).

☐ All plots are contour plots with outliers or pseudocolor plots.

☒ A numerical value for number of cells or percentage (with statistics) is provided.

Methodology

Sample preparation	We re-estimated the genome size of Lo7 using the following procedure. Nuclear suspensions were prepared by co-chopping ~0.5 cm^2 of fresh young leaf tissue together with Pisum sativum L. subsp. sativum convar. sativum var. ponderosum Alef. 'Kifejtö Borsó' (Genebank accession number: PIS 630) as an internal reference standard in a Petri dish using the CyStain PI Absolute P reagent kit (Sysmex-Partec), according to the manufacturer's instructions.
Instrument	CytoFLEX flow cytometer (Beckman Coulter)
Software	n/a
Cell population abundance	n/a
Gating strategy	n/a

☐ Tick this box to confirm that a figure exemplifying the gating strategy is provided in the Supplementary Information.
