# Supplementary material for: Unveiling centromeric retrotransposon dynamics through a high-quality rye genome assembly
Source: Nat Commun. 2026 Aug 15;17:8413. doi: 10.1038/s41467-026-76753-4 (PMC13477480; doi:10.1038/s41467-026-76753-4)
Supplement: Supplementary file 2 [file 41467_2026_76753_MOESM1_ESM.pdf]

Unveiling centromeric retrotransposon dynamics through a high-quality rye genome assembly

Chen *et al.*

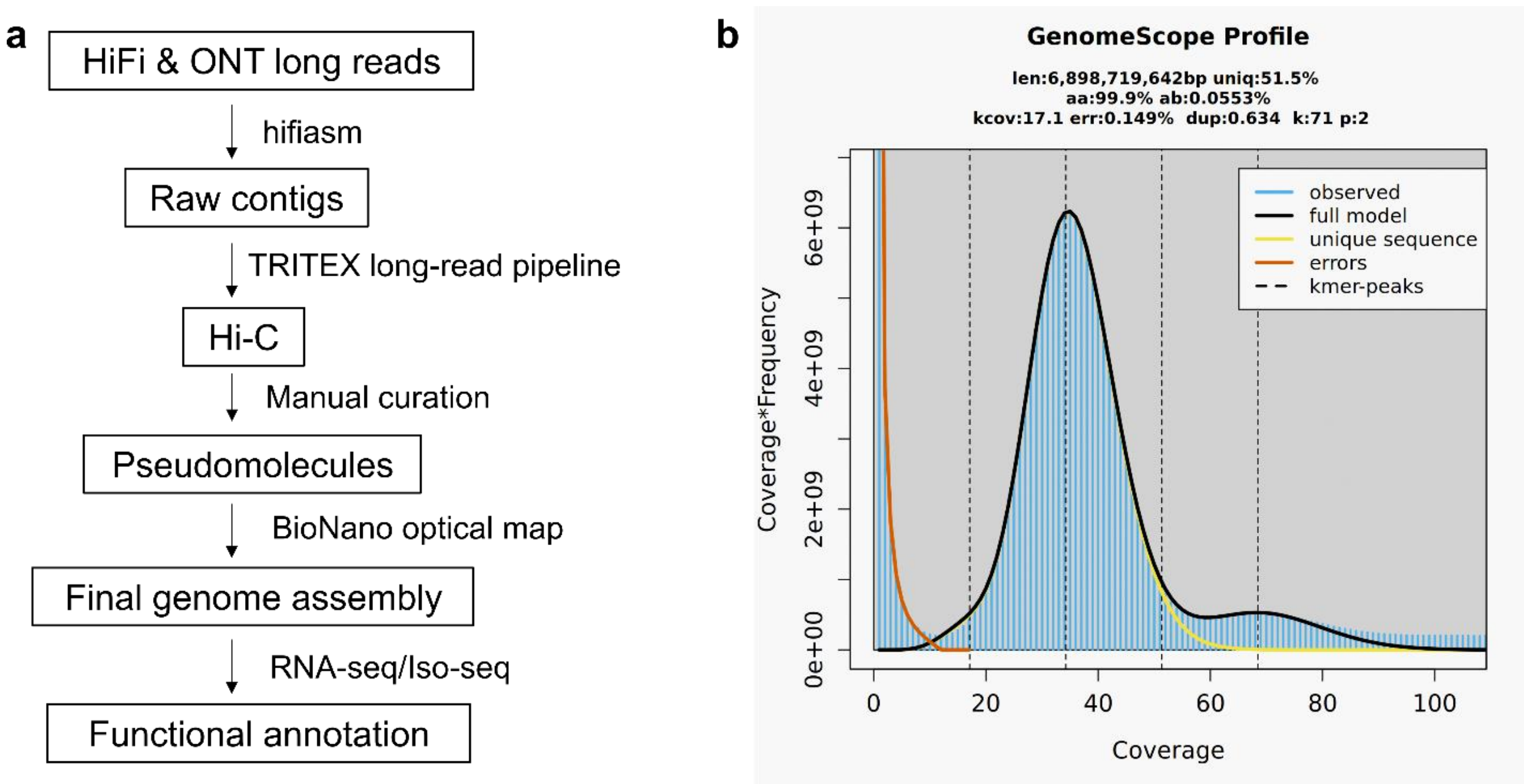

Supplementary Fig. 1. Genome assembly pipeline and heterozygosity estimation of Lo7. a Genome assembly and functional annotation pipeline of Lo7_V3. **b** Heterozygosity estimation of Lo7. Heterozygosity was determined by GenomeScope 2.0[1], based on k-mer (71) analysis of HiFi reads.

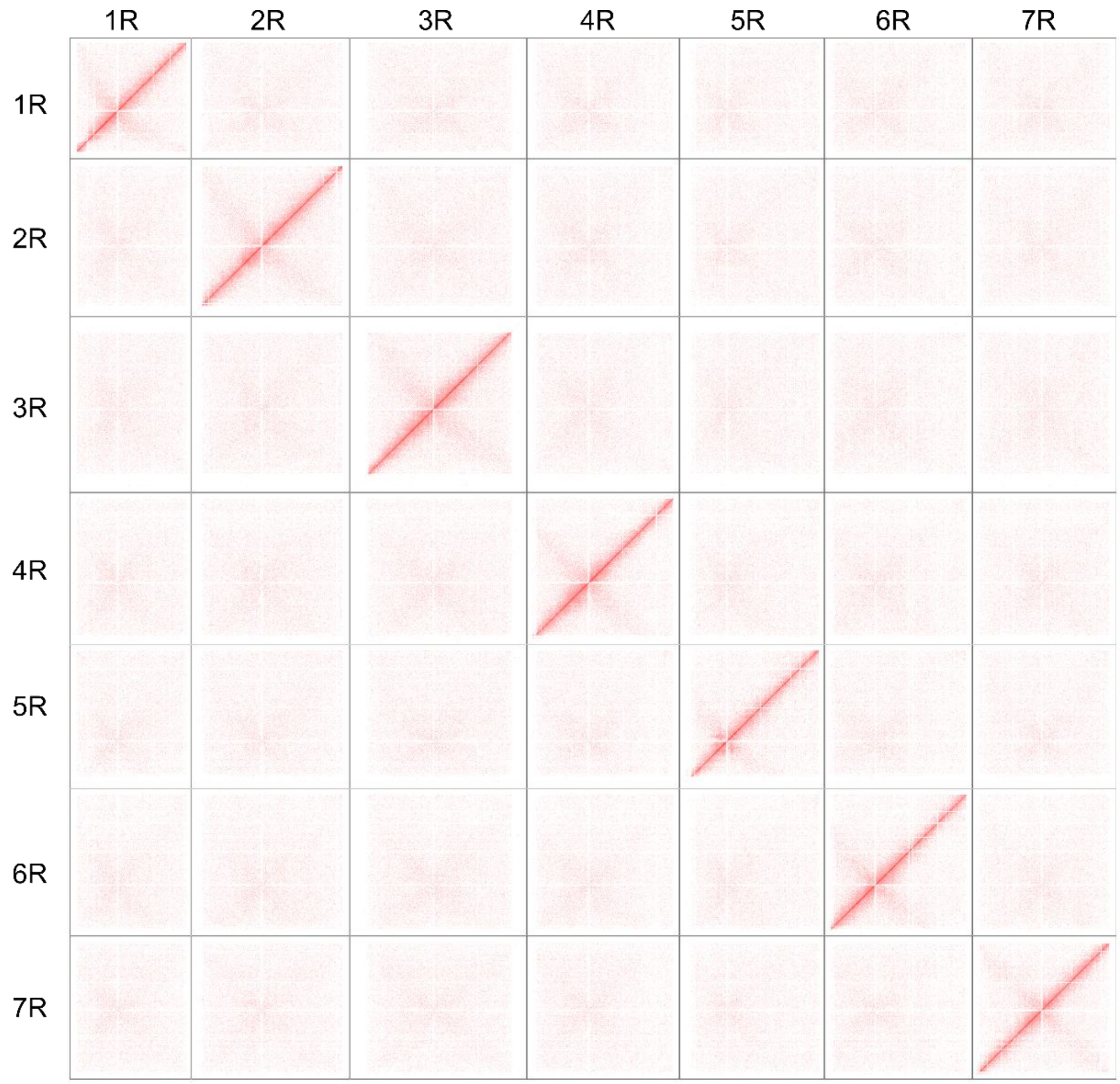

Supplementary Fig. 2. Hi-C contact map of Lo7_V3. Hi-C contact matrix visualized at 200 kb resolution shows chromatin interaction frequency across the assembled rye chromosomes. The intensity of each pixel reflects the contact frequency between two genomic regions. Centromeric and pericentromeric regions exhibit reduced contact frequencies.

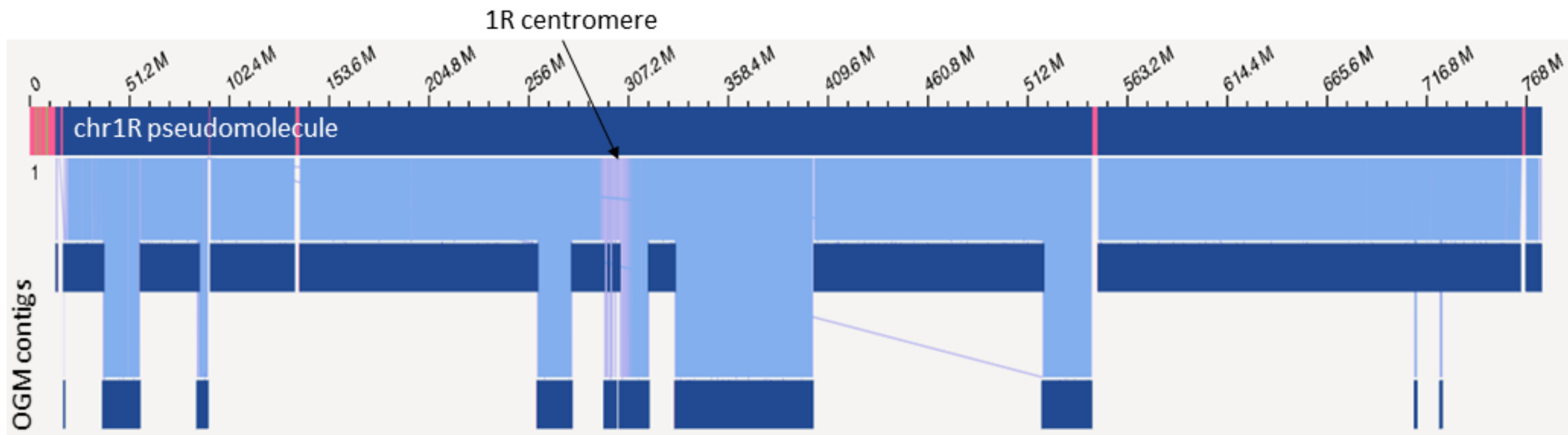

Supplementary Fig. 3. Validation of pseudomolecule assembly using optical genome map. Optical genome map (OGM) contigs were aligned to chromosome 1R pseudomolecule, confirming structural consistency, including in the centromeric region.

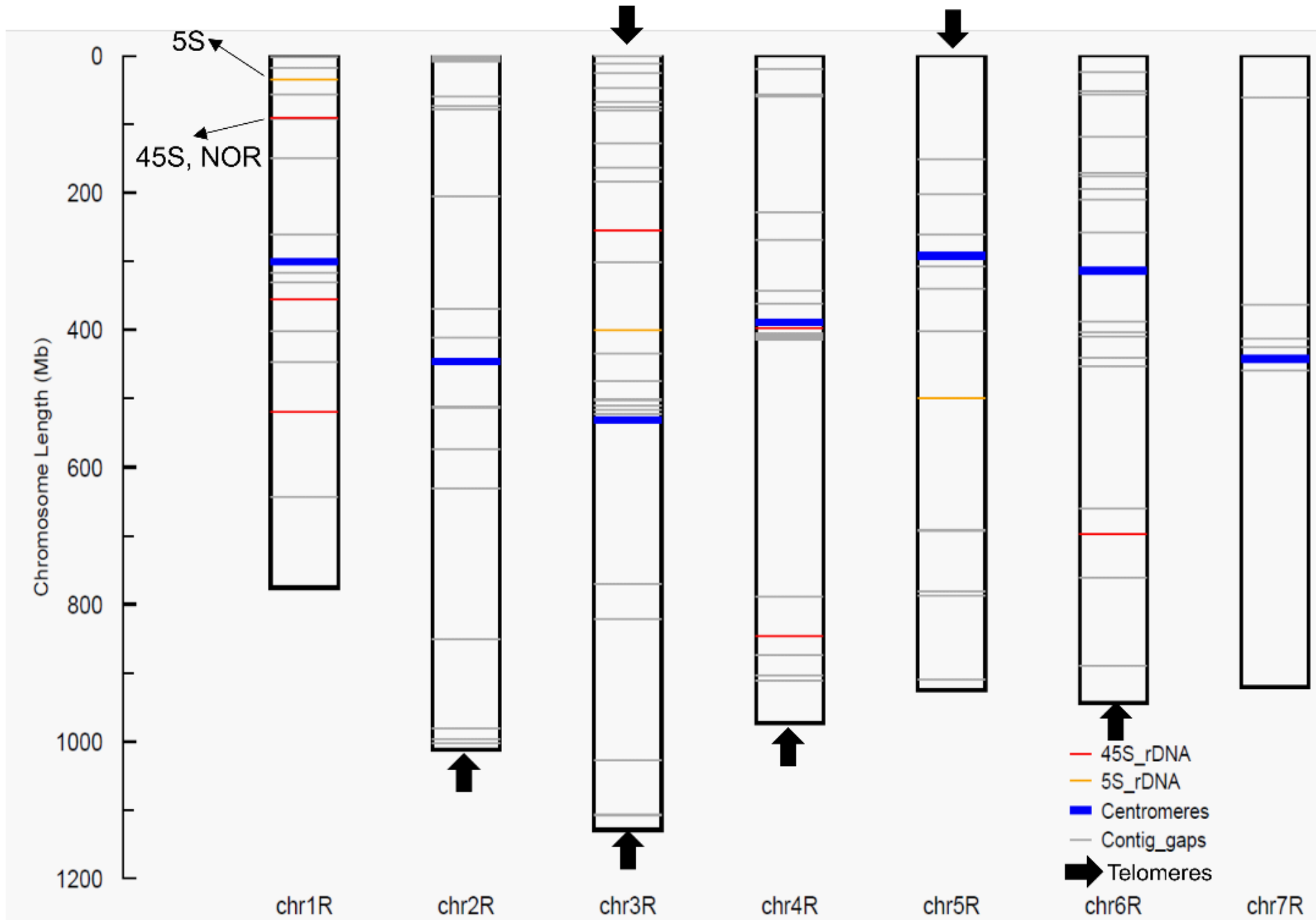

Supplementary Fig. 4. Assessment of genome assembly completeness. Visualization of 5S and 45S rDNA, centromeres, telomeres, and contig gaps across the seven chromosomes of Lo7_V3.

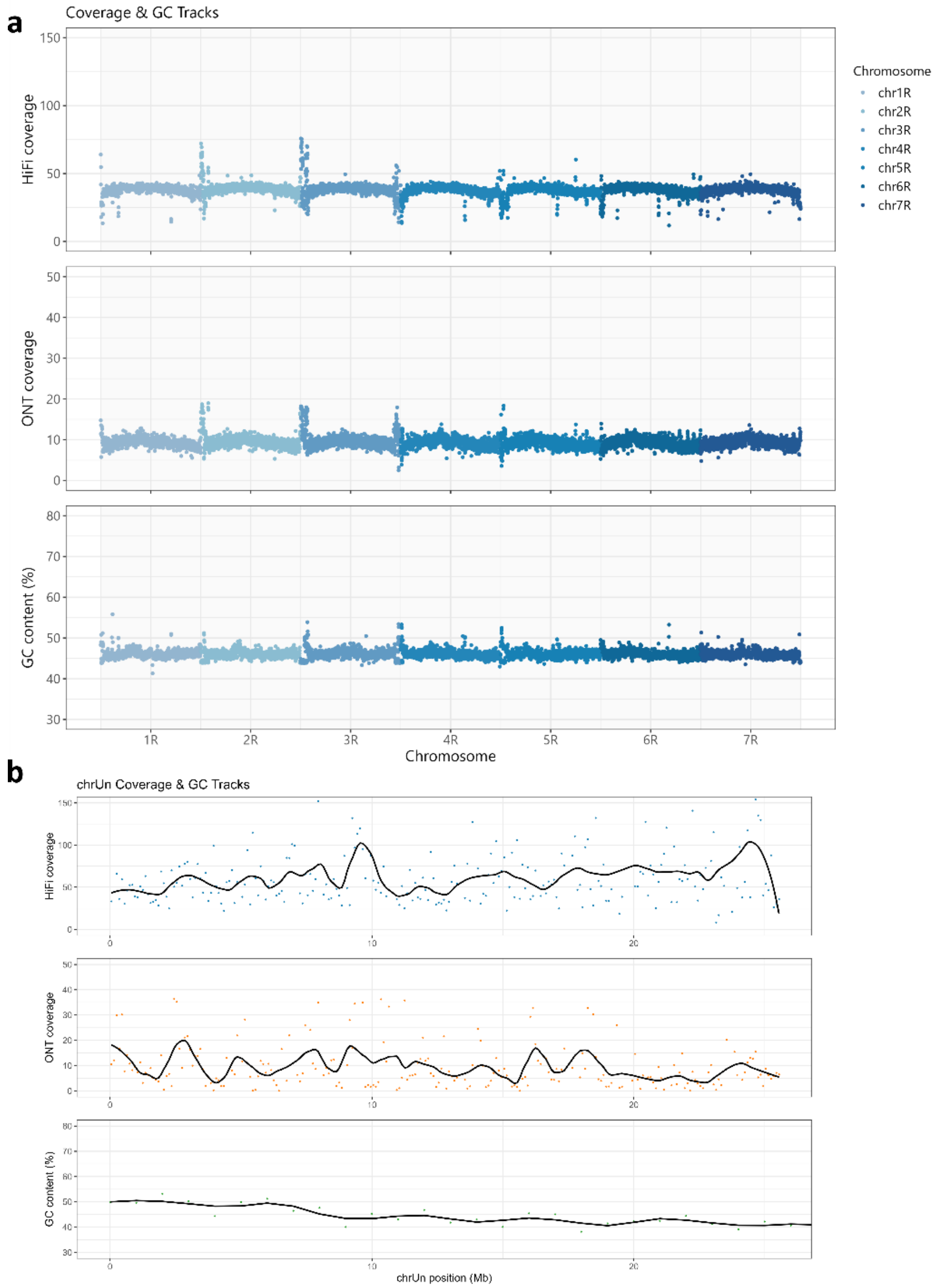

Supplementary Fig. 5. Genome-wide distribution of HiFi and ONT coverage alongside GC content. For chromosomes 1R–7R (**a**), HiFi coverage, ONT coverage, and GC content were calculated in 1 Mb windows. For chromosome Un (**b**), HiFi and ONT coverages were calculated in 100 kb windows, whereas GC content was calculated in 1 Mb windows. The *x*-axis shows chromosomal position, and the *y*-axis shows HiFi coverage, ONT coverage, and GC content. The plot highlights variation in sequencing depth and GC content across the genome and identifies regions with potential GC-associated coverage biases (Supplementary Table 5). Source data are provided as a Source Data file.

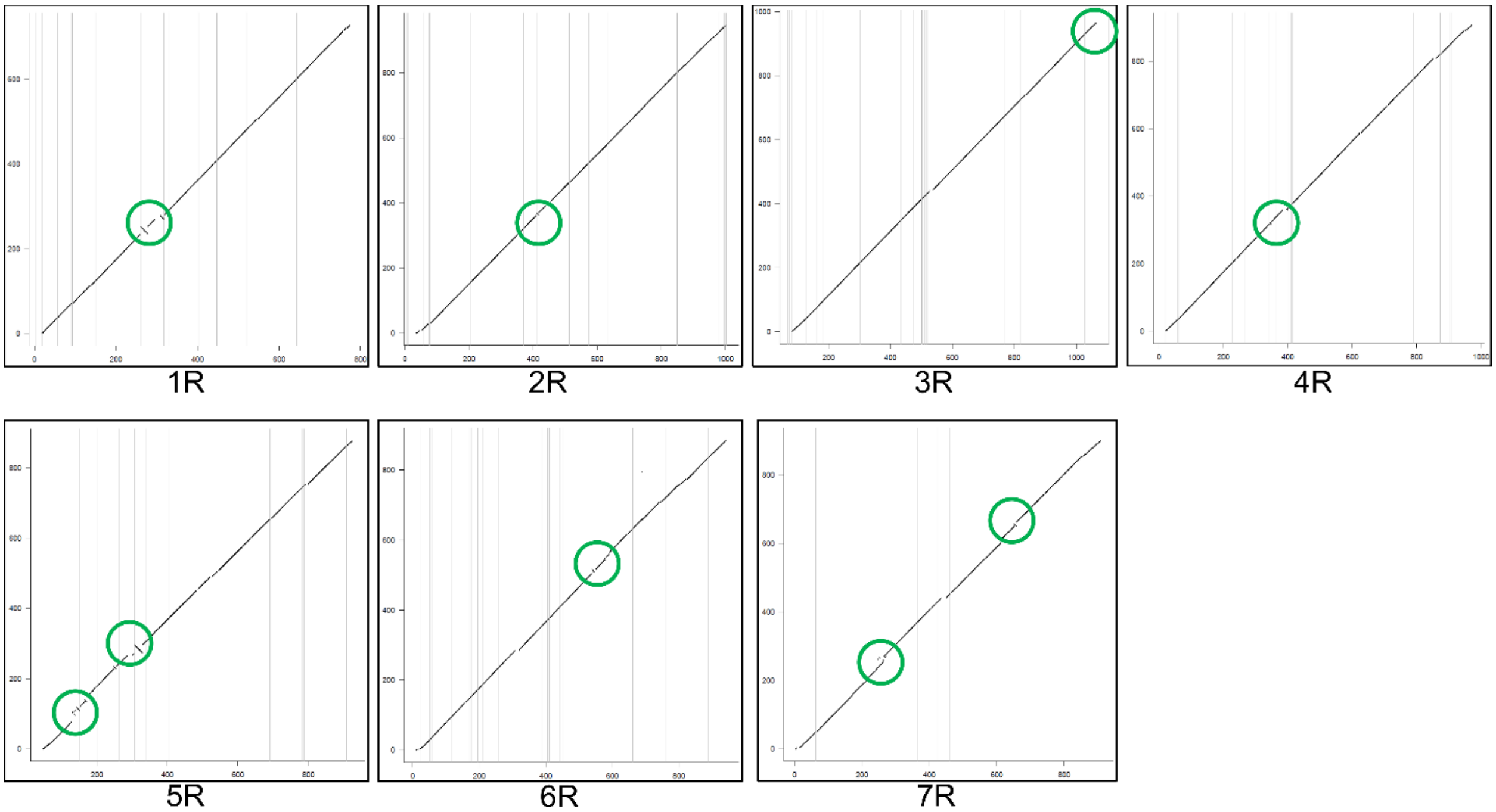

Supplementary Fig. 6. Collinearity plot between Lo7_V2 and Lo7_V3. Each green circle indicates a region of orientation error identified in Lo7_V2. *x*-axis, Lo7_V3; *y*-axis, Lo7_V2.

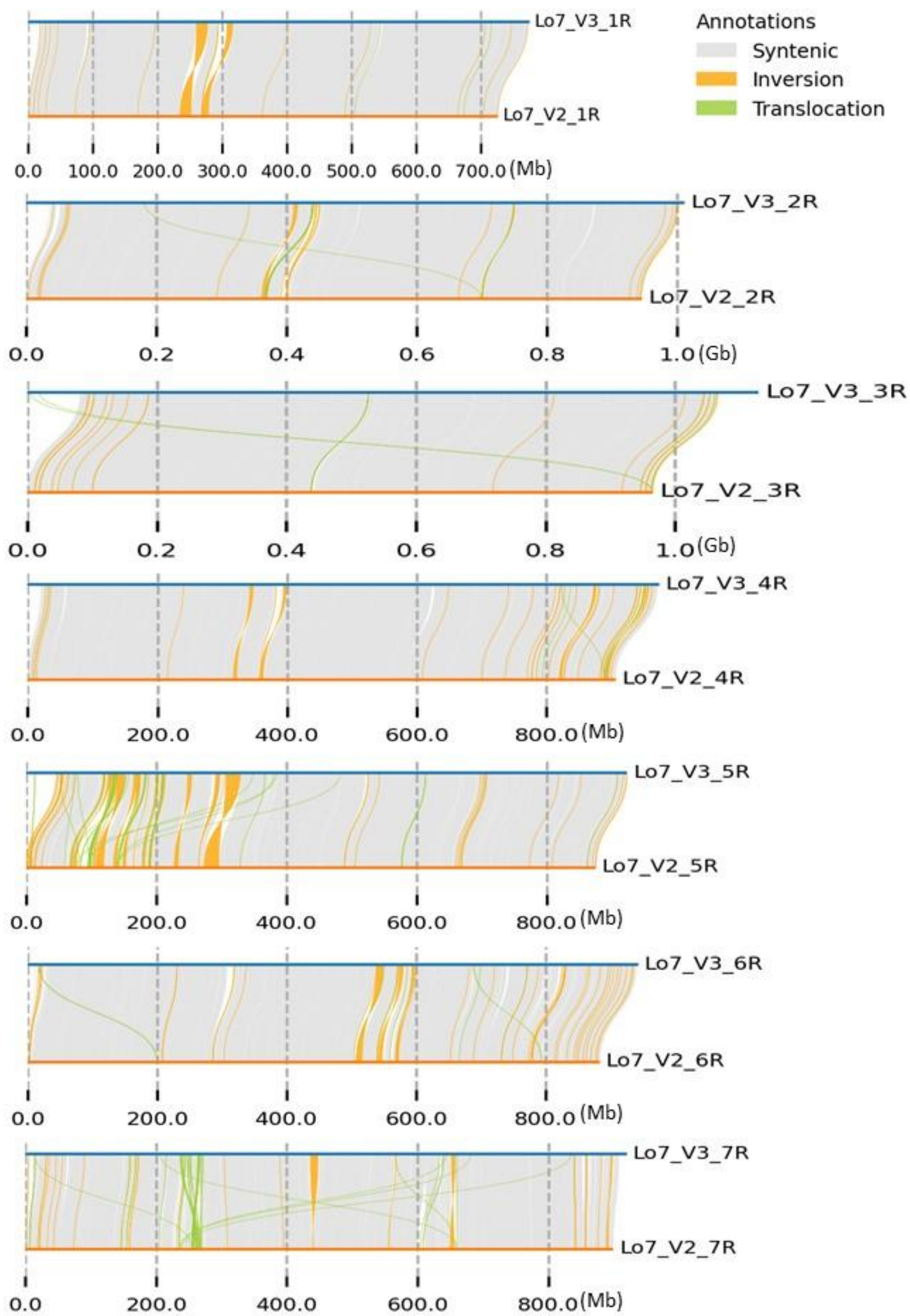

Supplementary Fig. 7. Comparative genomic synteny between Lo7_V2 and Lo7_V3 revealed by SyRI[2]. Diagonal alignments of 1-7R indicate conserved collinear regions, while interruptions or shifts reveal structural variations such as inversions and translocations. The plot highlights the overall high collinearity between Lo7_V2 and Lo7_V3, with improvements in scaffolding and corrected misassemblies in Lo7_V3.

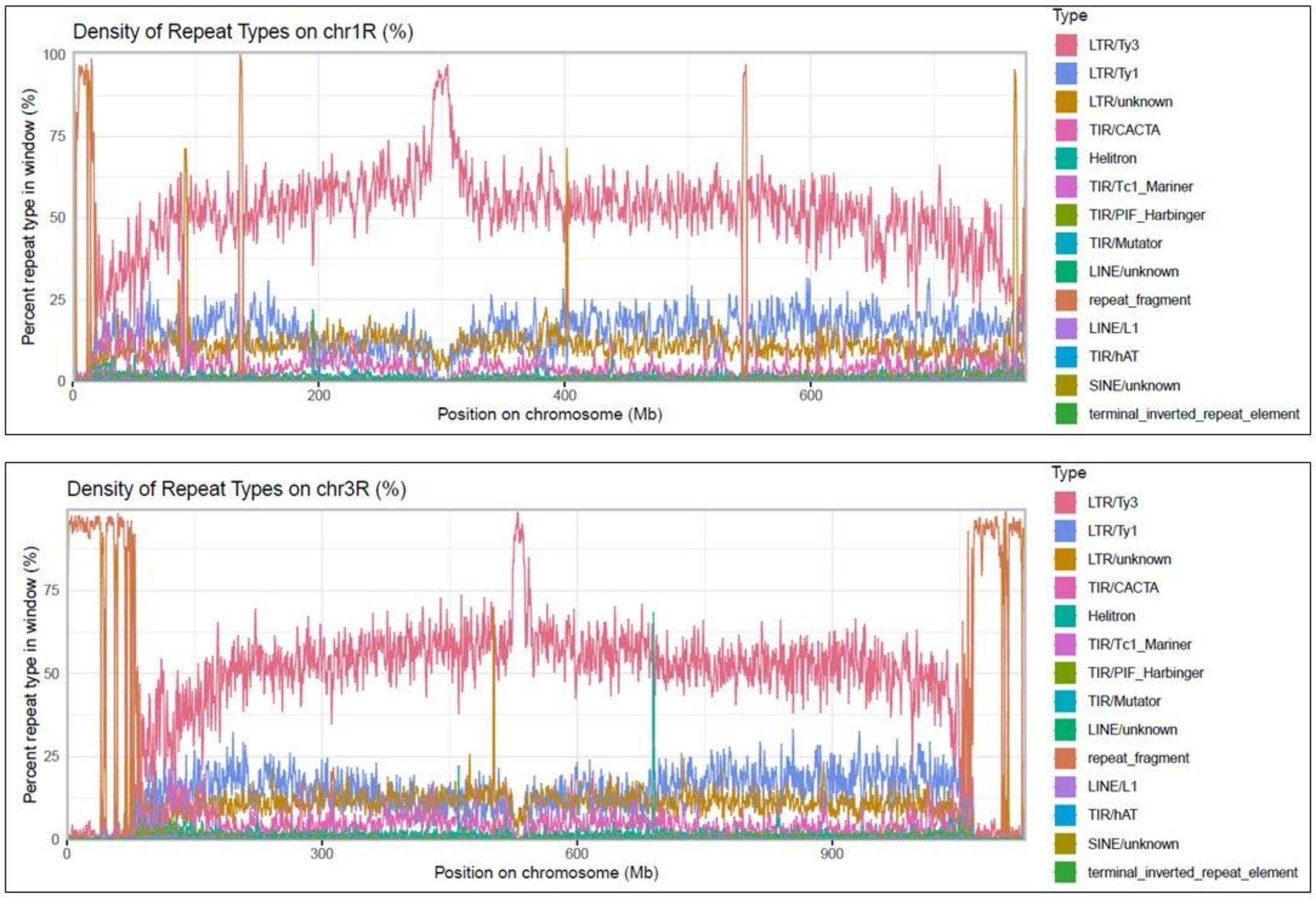

Supplementary Fig. 8. Repeat content across the genome was identified and classified using EDTA. The density of major repeat types, including LTR retrotransposons (*Gypsy* and *Copia*), DNA transposons, and unclassified elements, was calculated in fixed genomic windows to visualize their distribution patterns along chromosomes 1R and 3R. DNA transposons were further classified into terminal inverted repeat (TIR) elements, Helitron (rolling-circle transposons), and non-LTR retrotransposons such as LINEs (long interspersed nuclear elements).

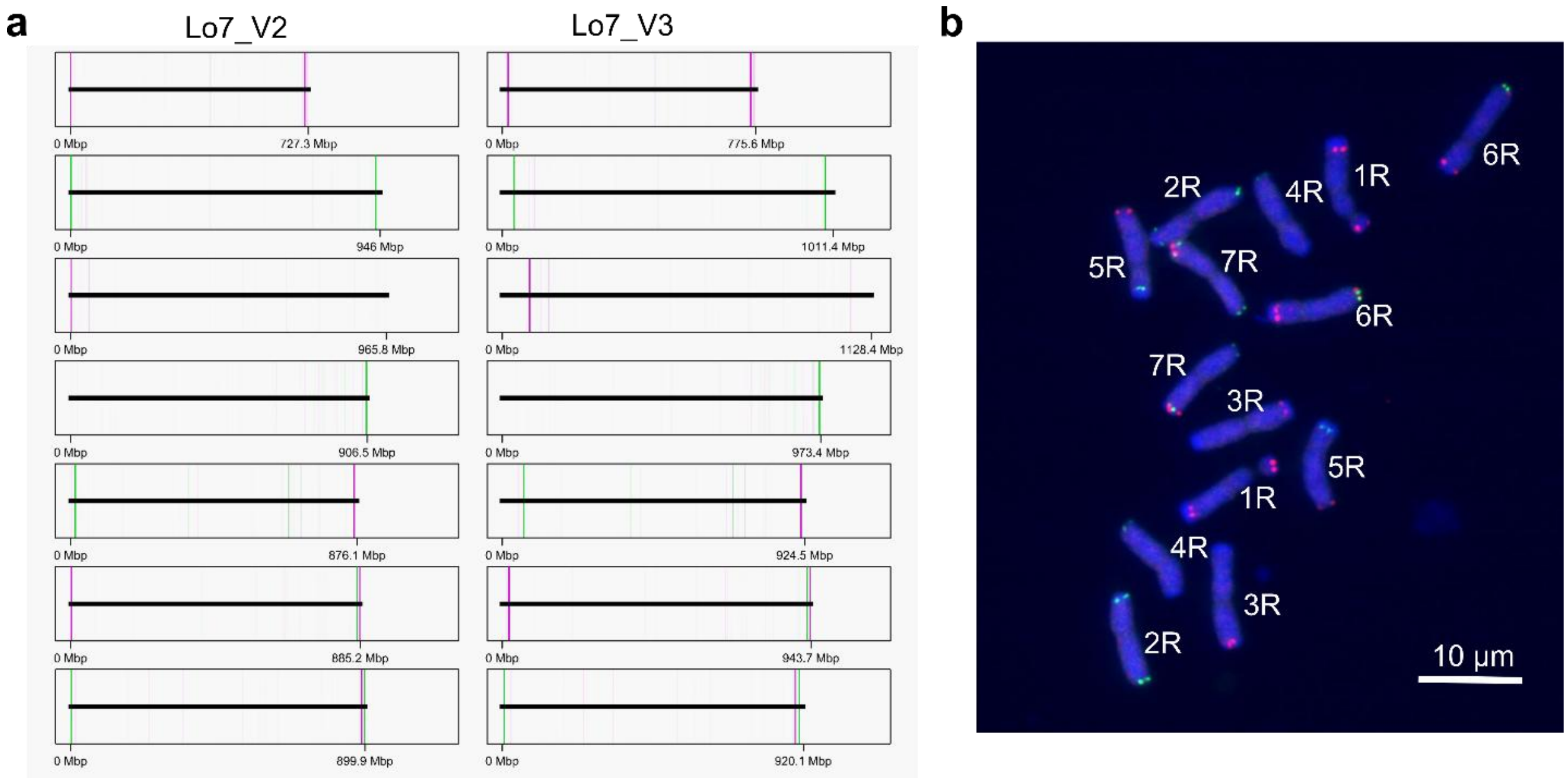

Supplementary Fig. 9. Painting probes were designed from Lo7_V2 and Lo7_V3 assemblies. **a** Probes were developed and visualized based on the two Lo7 genome assemblies. **b** Fluorescence in situ hybridization (FISH) experiments were performed using the painting probes derived from *in silico* computation. Scale bar: 10 µm.

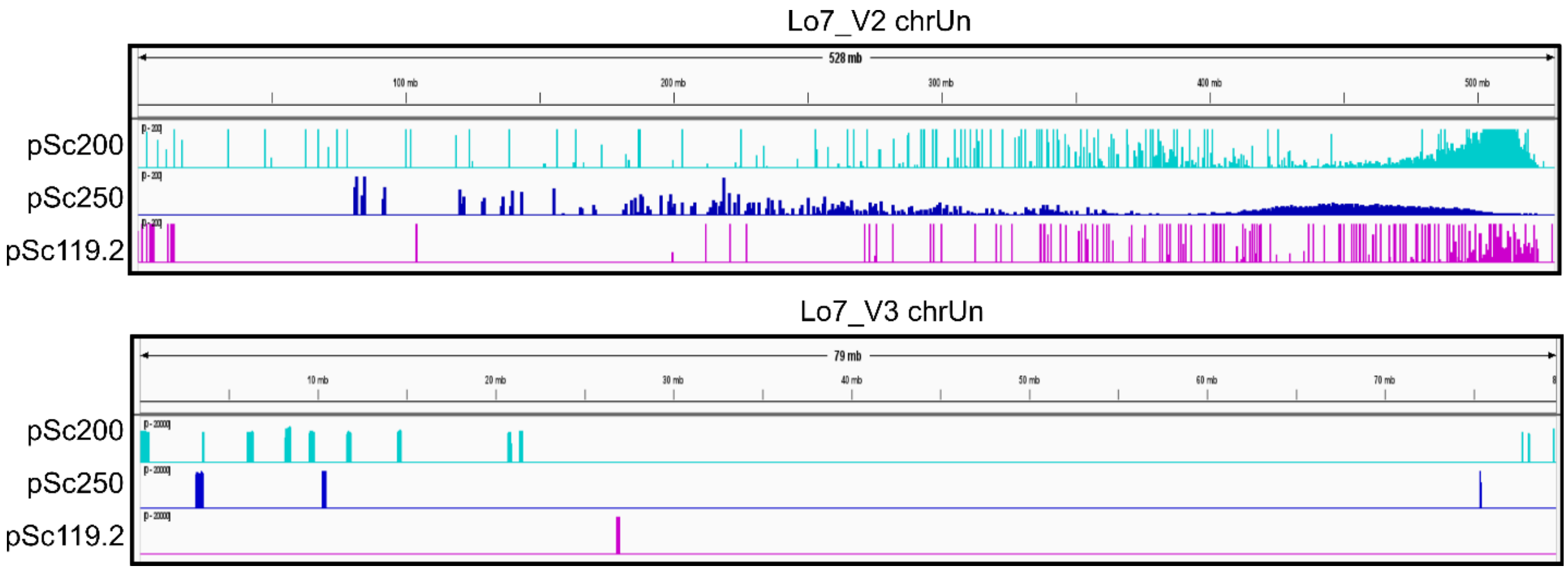

Supplementary Fig. 10. Visualization of pSc200, pSc250 and pSc119.2 distribution in unanchored parts of two Lo7 assemblies. chrUn, unanchored region.

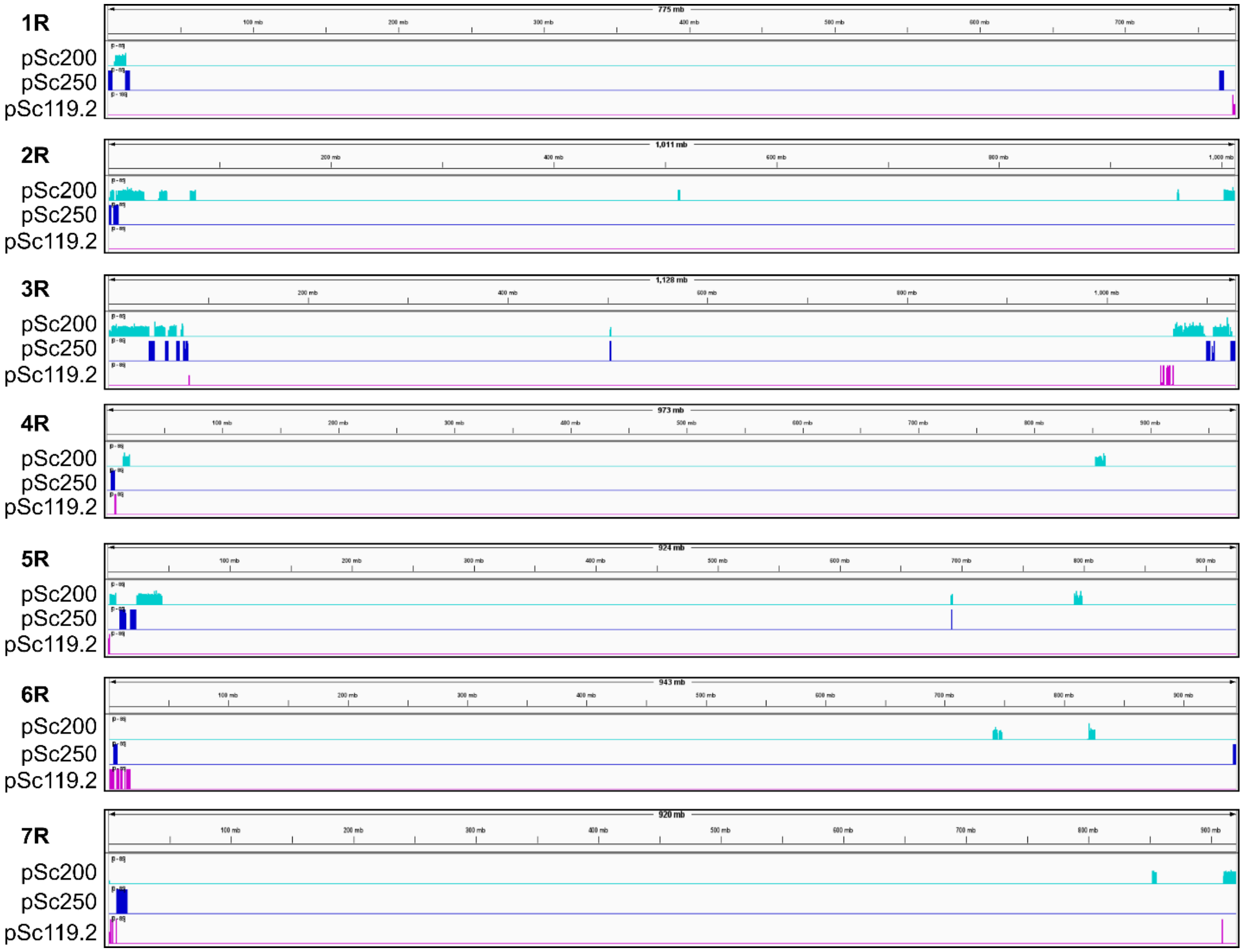

Supplementary Fig. 11. Visualization of pSc200, pSc250 and pSc119.2 distribution from Lo7 chromosome 1R-7R.

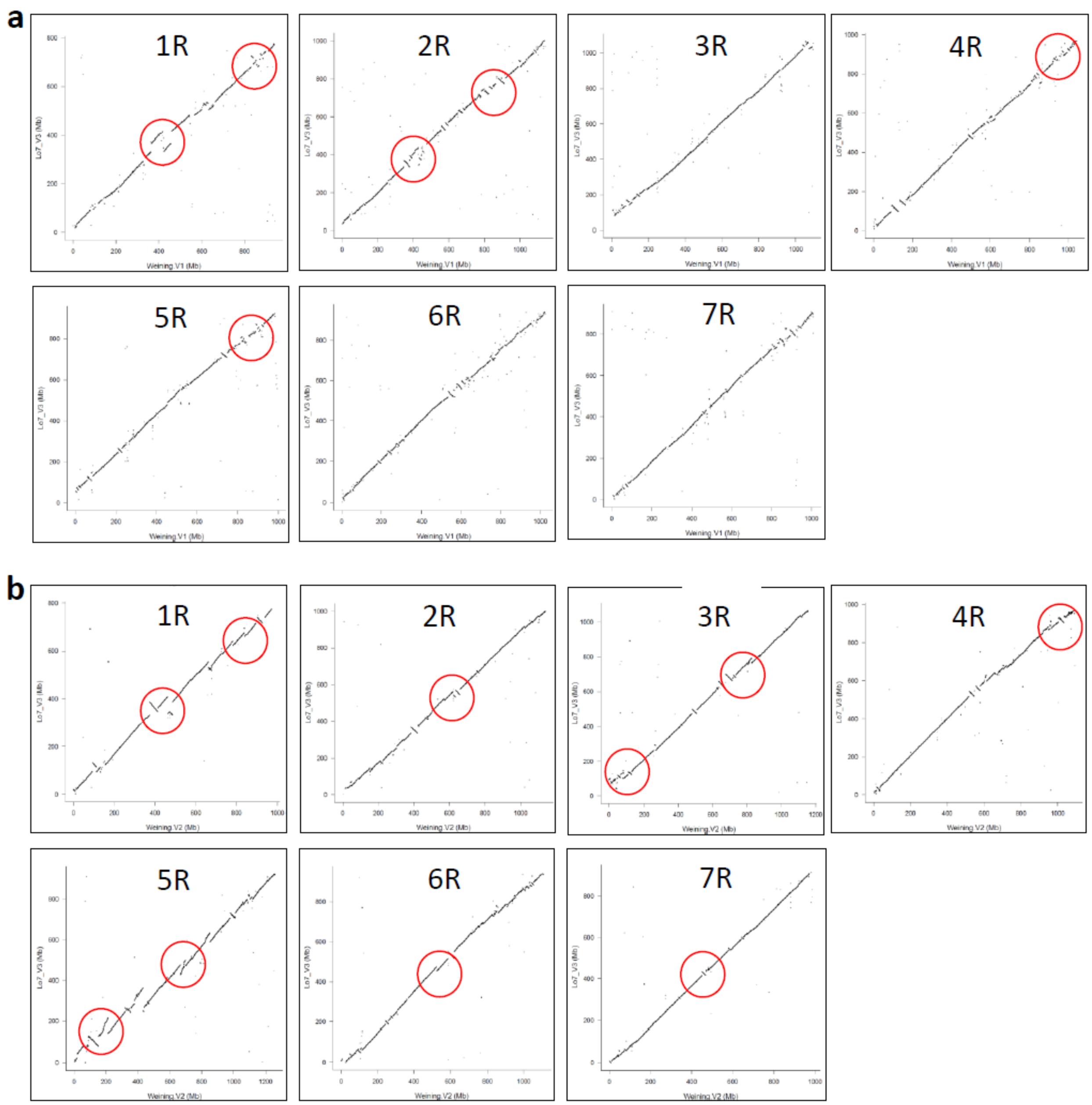

Supplementary Fig. 12. Collinearity plot between Lo7_V3, Weining.V1, and Weining.V2. Each red circle indicates a region of collapsed or redundant haplotypes identified in (**a**) Weining.V1[3] or (**b**) Weining.V2[4]. *x*-axis, Weining.V1 or Weining.V2; *y*-axis, Lo7_V3.

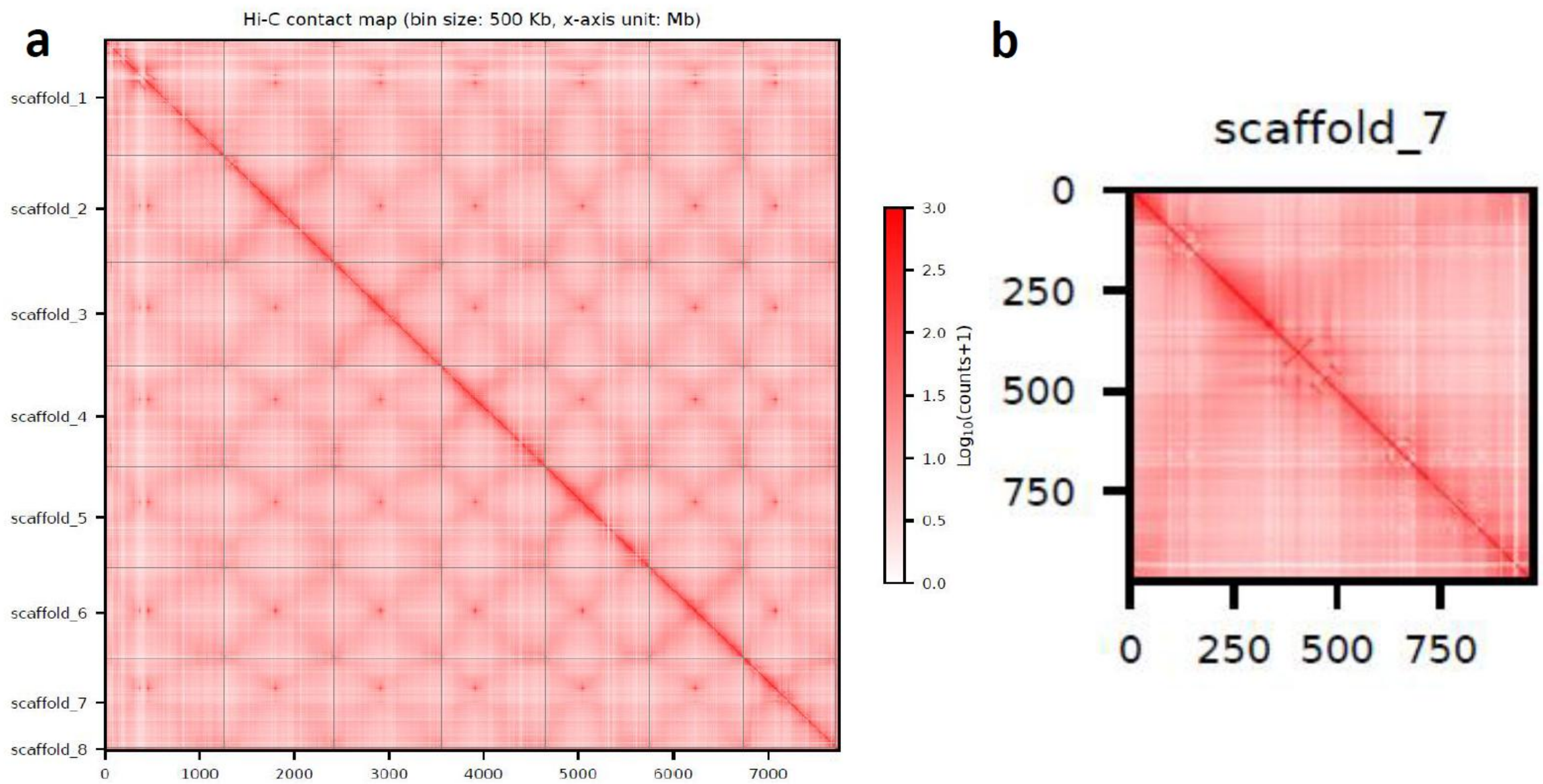

Supplementary Fig. 13. Hi-C interaction plot for the published Weining Hi-C data on the Weining.V2 assembly [4]. a Genome-wide Hi-C interaction matrix. scaffold_1-7, chromosome 1R-7R. scaffold_8, chromosome Un. **b** Detailed view of an example on chromosome 7R for collapsed assembly issues.

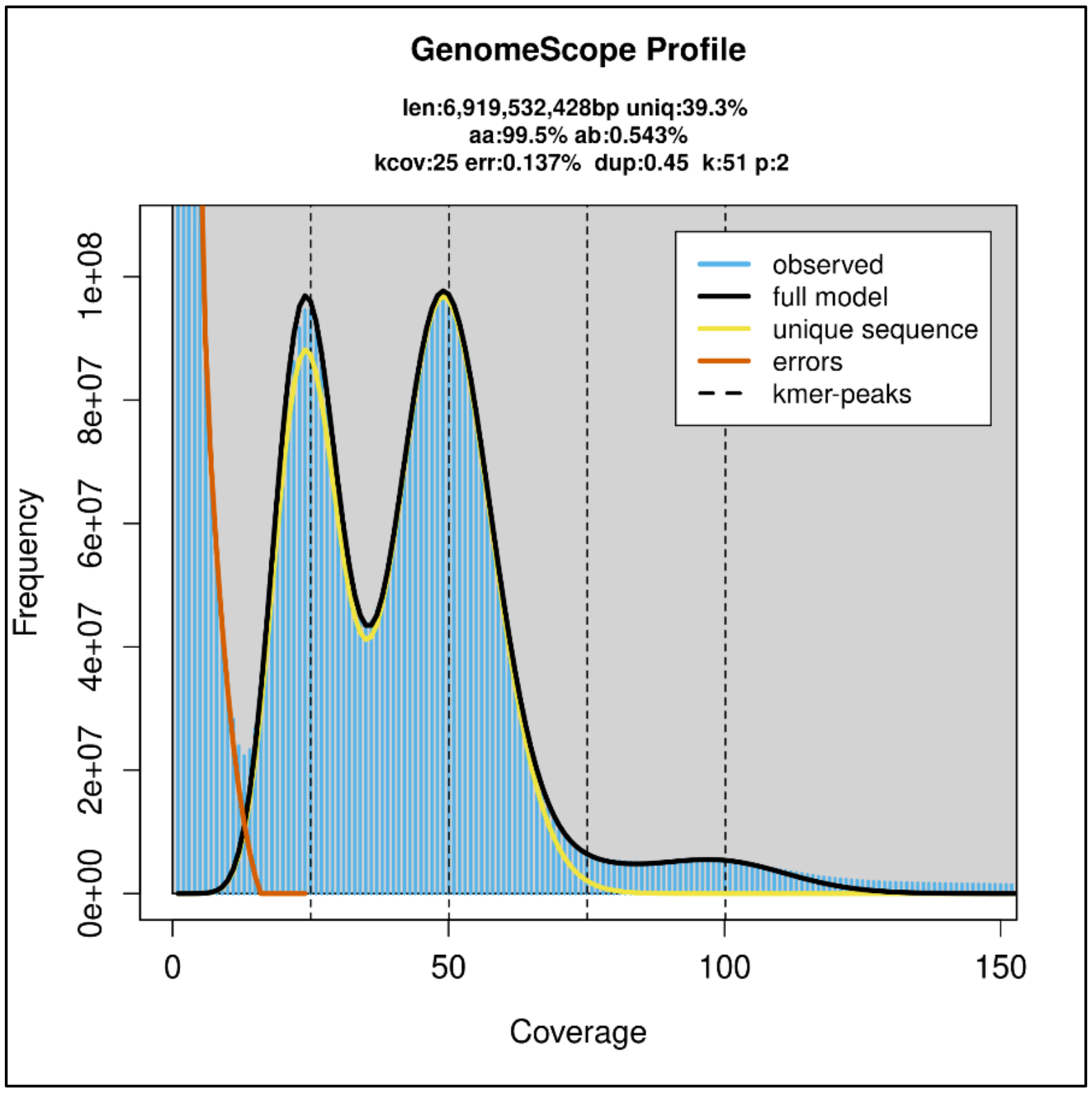

Supplementary Fig. 14. Heterozygosity estimation of rye Weining. Raw HiFi data was retrieved from published datasets (National Genomics Data Center (NGDC), China, under BioProject PRJCA041046)[4].

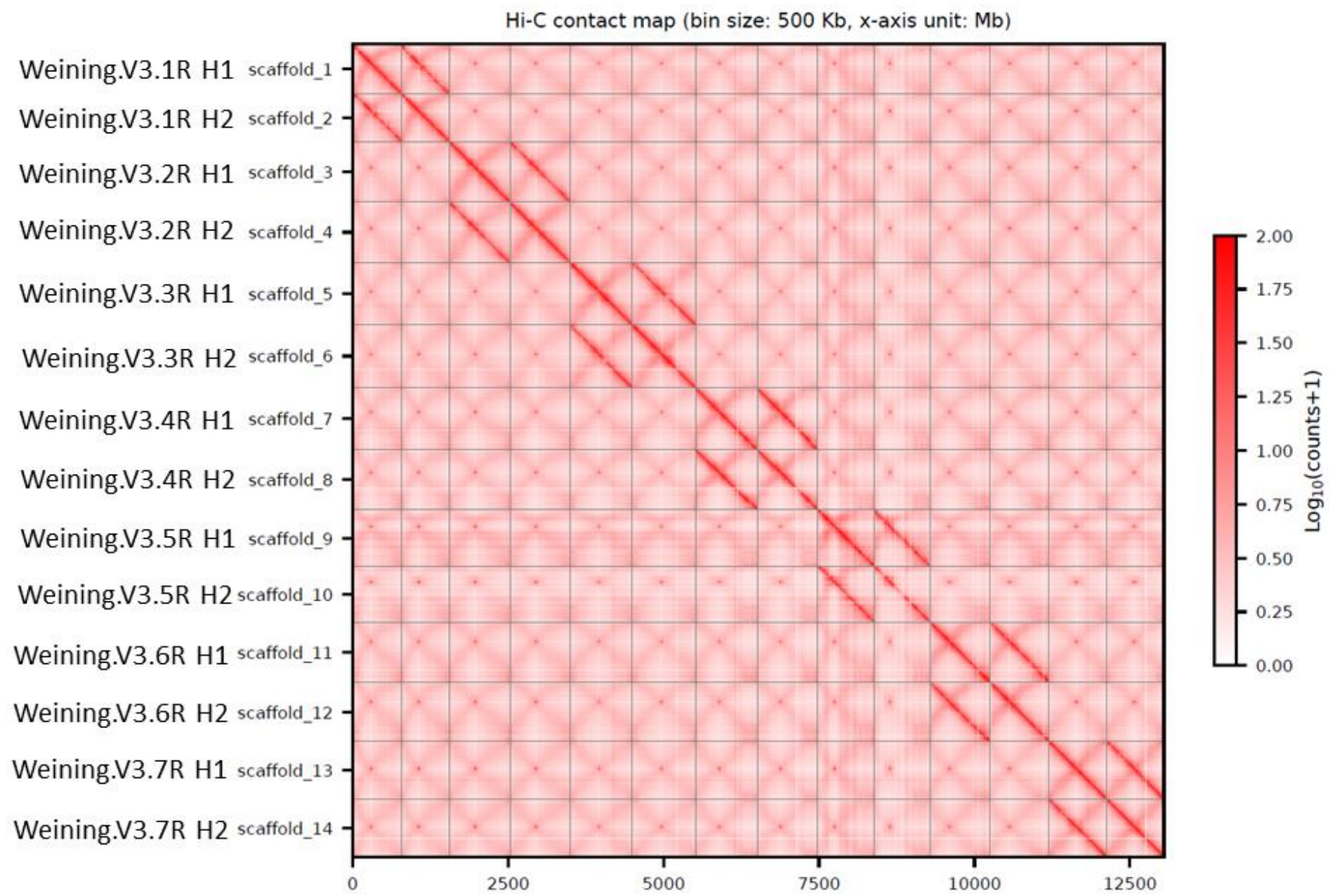

Supplementary Fig. 15. Hi-C contact map of Weining.V3. Hi-C contact matrix visualized at 500 kb resolution shows chromatin interaction frequency across the assembled rye chromosomes. The intensity of each pixel reflects the contact frequency between two genomic regions.

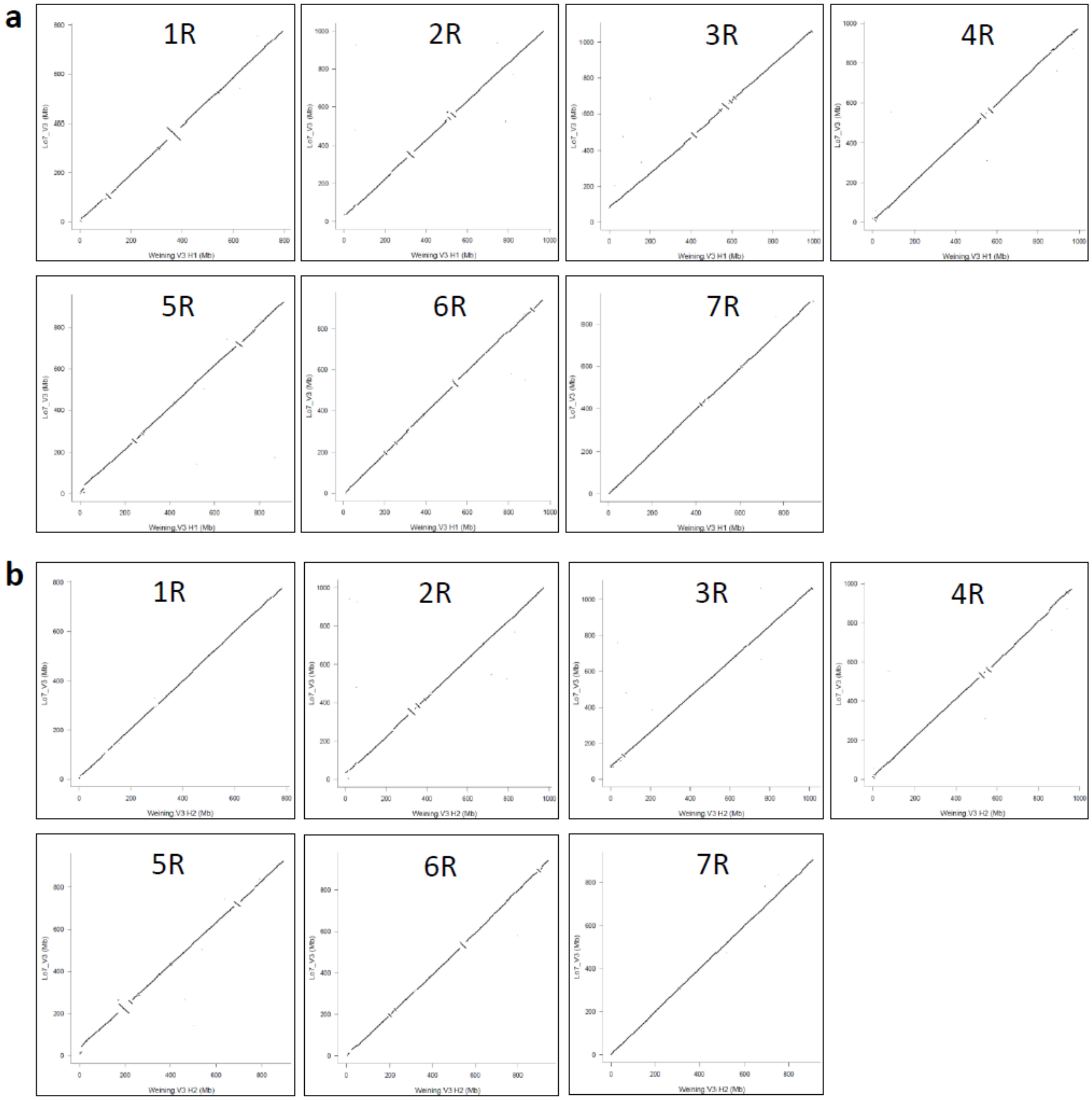

Supplementary Fig. 16. Collinearity plot between Weining.V3 H1, Weining.V3 H2, and Lo7_V3. *x*-axis, Weining.V3 H1 (**a**) or Weining.V3 H2 (**b**); *y*-axis, Lo7_V3.

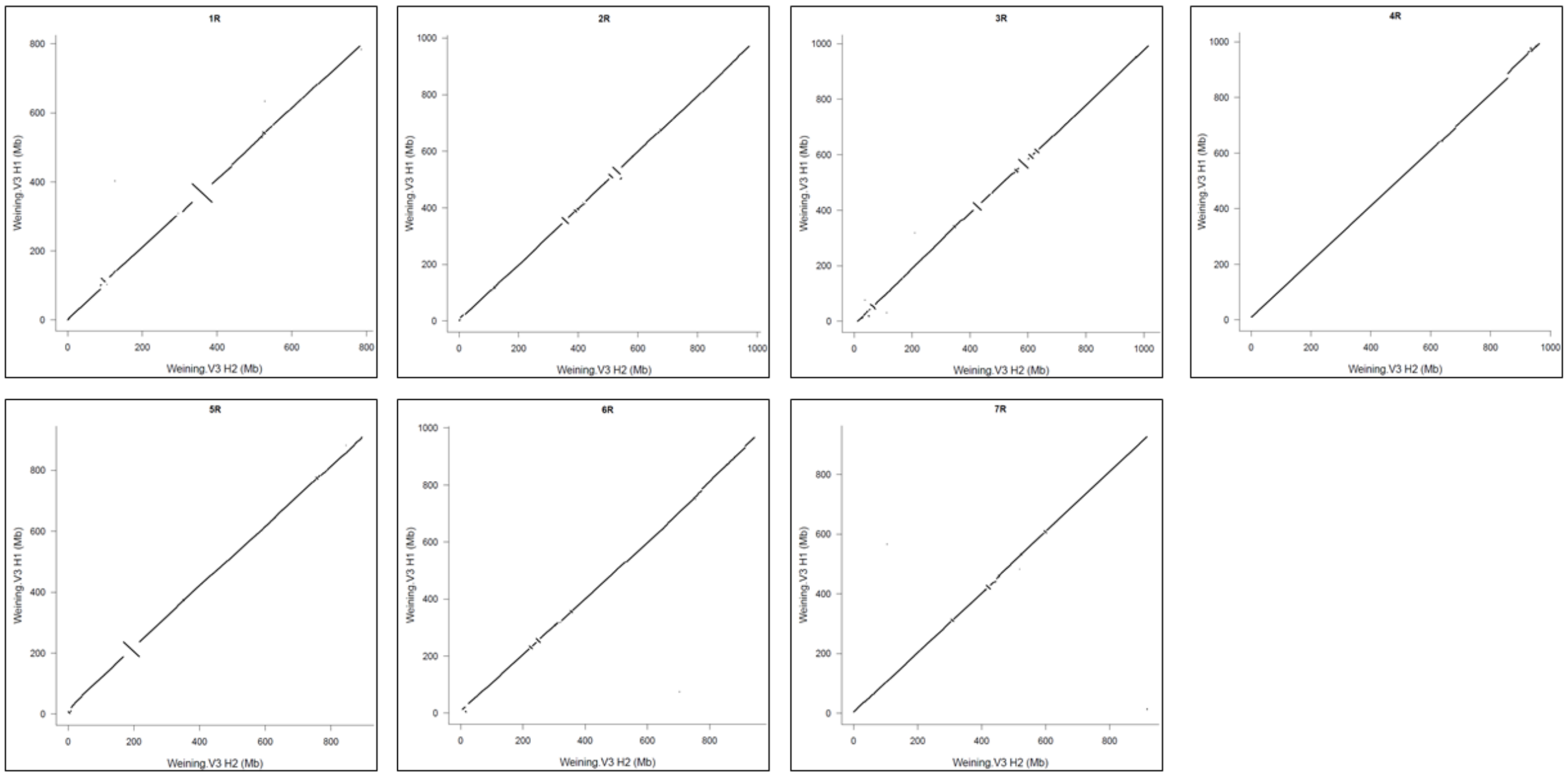

Supplementary Fig. 17. Collinearity plot between Weining.V3 H1 and Weining.V3 H2. *x*-axis, Weining.V3 H2; *y*-axis, Weining.V3 H1.

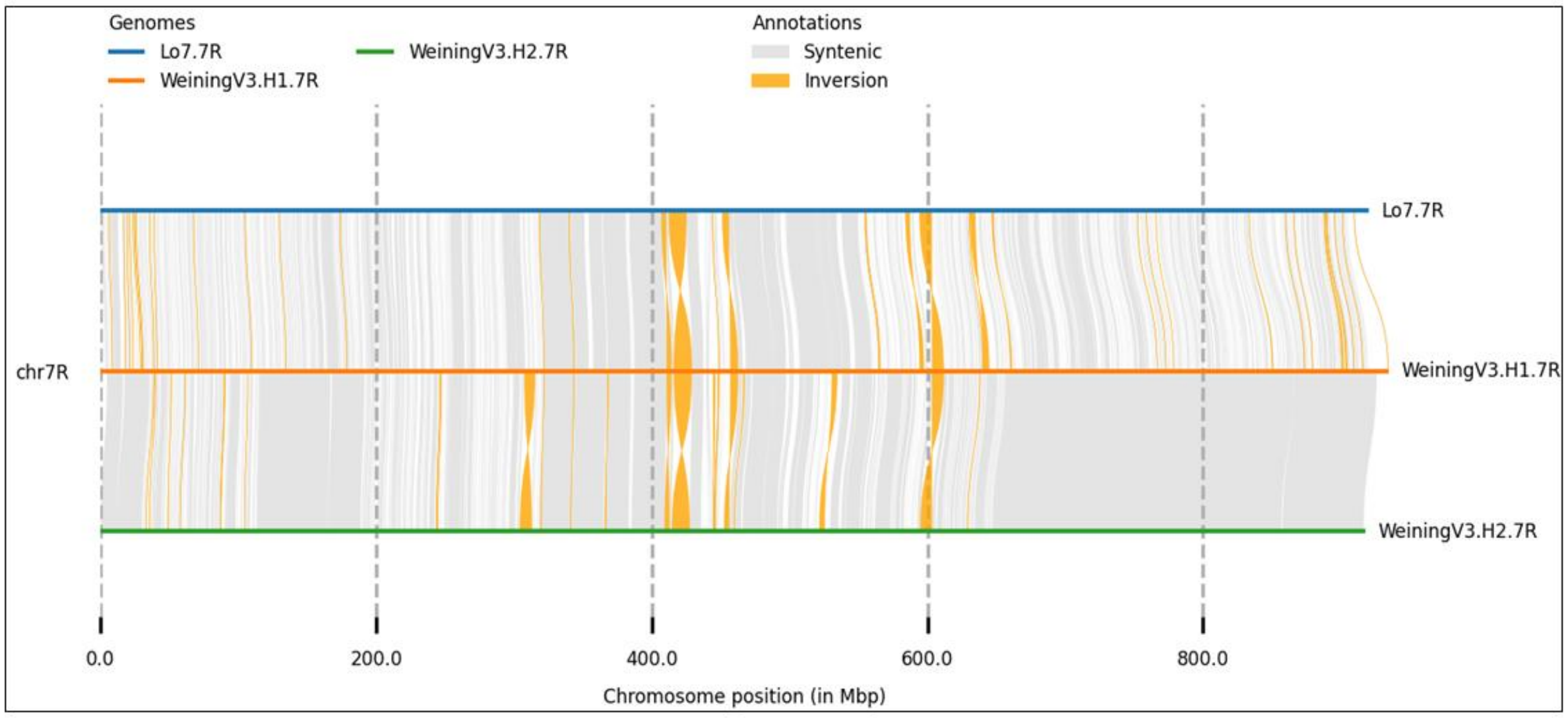

Supplementary Fig. 18. Comparative genomic synteny on chromosome 7R between Lo7_V3, Weining.V3 H1 and Weining.V3 H2. Diagonal alignments of chromosome 7R indicate conserved collinear regions, while shifts reveal structural variations such as inversions. H1, haplotype1; H2, haplotype2.

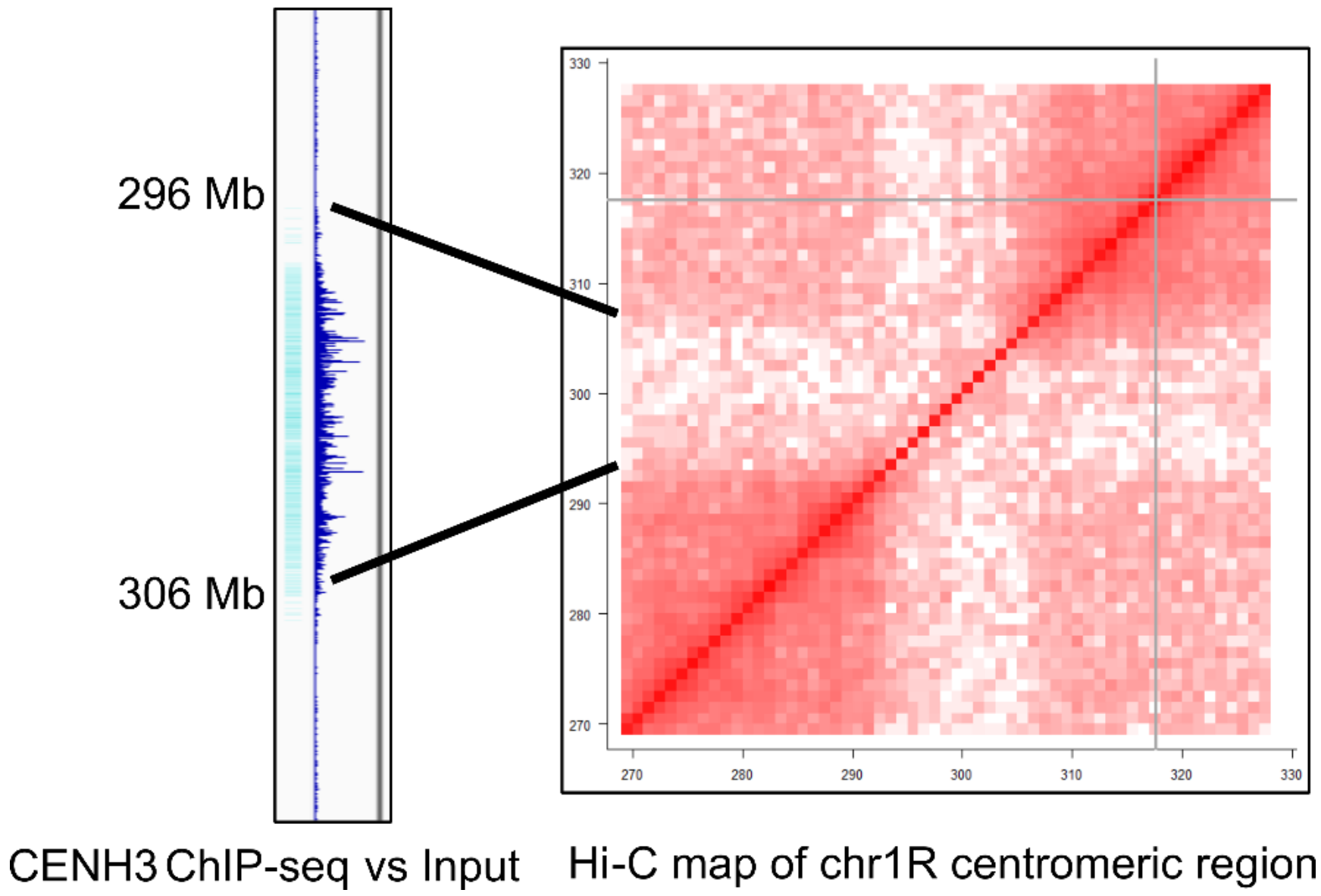

Supplementary Fig. 19. Centromere size predictions of Lo7_V3. Seven rye centromeres were identified and their sizes were determined (Supplementary Table 11**)**. Identification of the centromeric region on chromosome 1R using CENH3 ChIP-seq data combined with Hi-C interaction analysis. Gray lines in Hi-C maps denote contig gap within chromosome 1R.

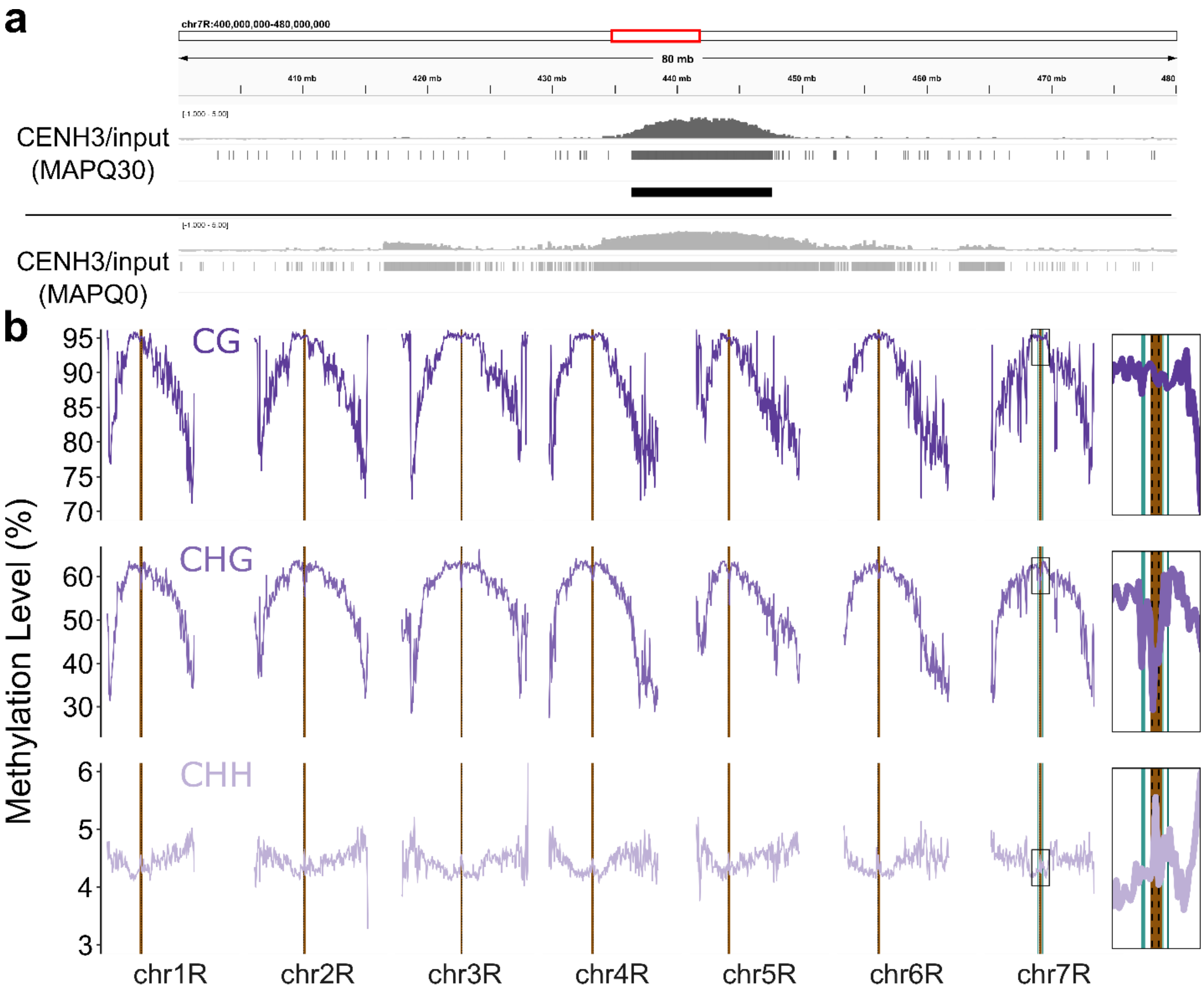

Supplementary Fig. 20. Functional Centromere Size Estimation of Lo7 Using CENH3 ChIP-seq and Whole-Genome Bisulfite Sequencing (WGBS) data. **a** CENH3 ChIP-seq data were analyzed using two mapping quality thresholds (MAPQ0, including all mapped reads, and MAPQ30, including only uniquely mapped reads) to define the centromeric region of chromosome 7R. **b** DNA methylation profiles (CG, CHG, and CHH contexts) across the seven centromeres were generated from WGBS data. Based on the combined CENH3 ChIP-seq and methylation analyses, centromeric regions in Lo7 were delineated. A zoomed-in view of the chromosome 7R centromere (right panel) reveals distinct CHG methylation dips compared with the surrounding pericentromeric regions.

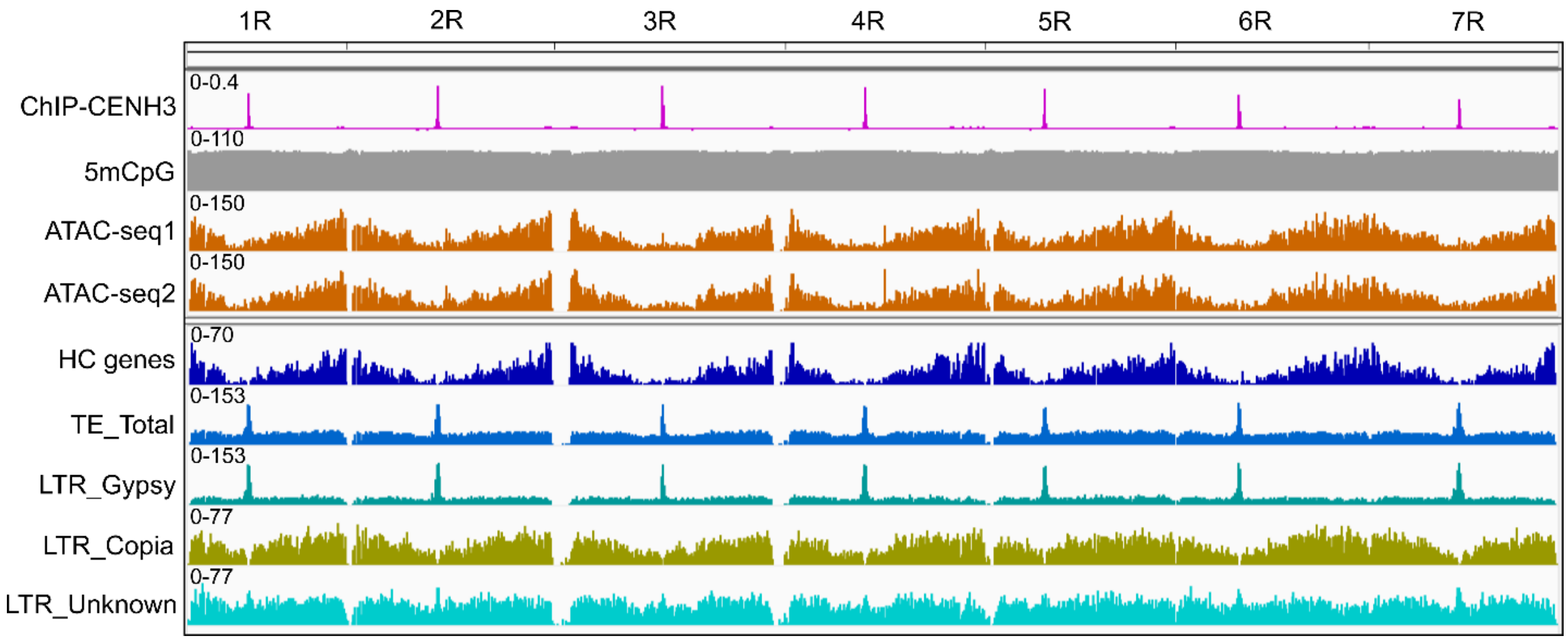

Supplementary Fig. 21. Structural analysis of seven rye complete centromeres. To investigate the structural organization of genes within centromeric regions, we integrated multiple genomic and epigenomic datasets, including CENH3 ChIP-seq, ATAC-seq, 5mCpG methylation, high-confidence gene annotations, and TE annotations.

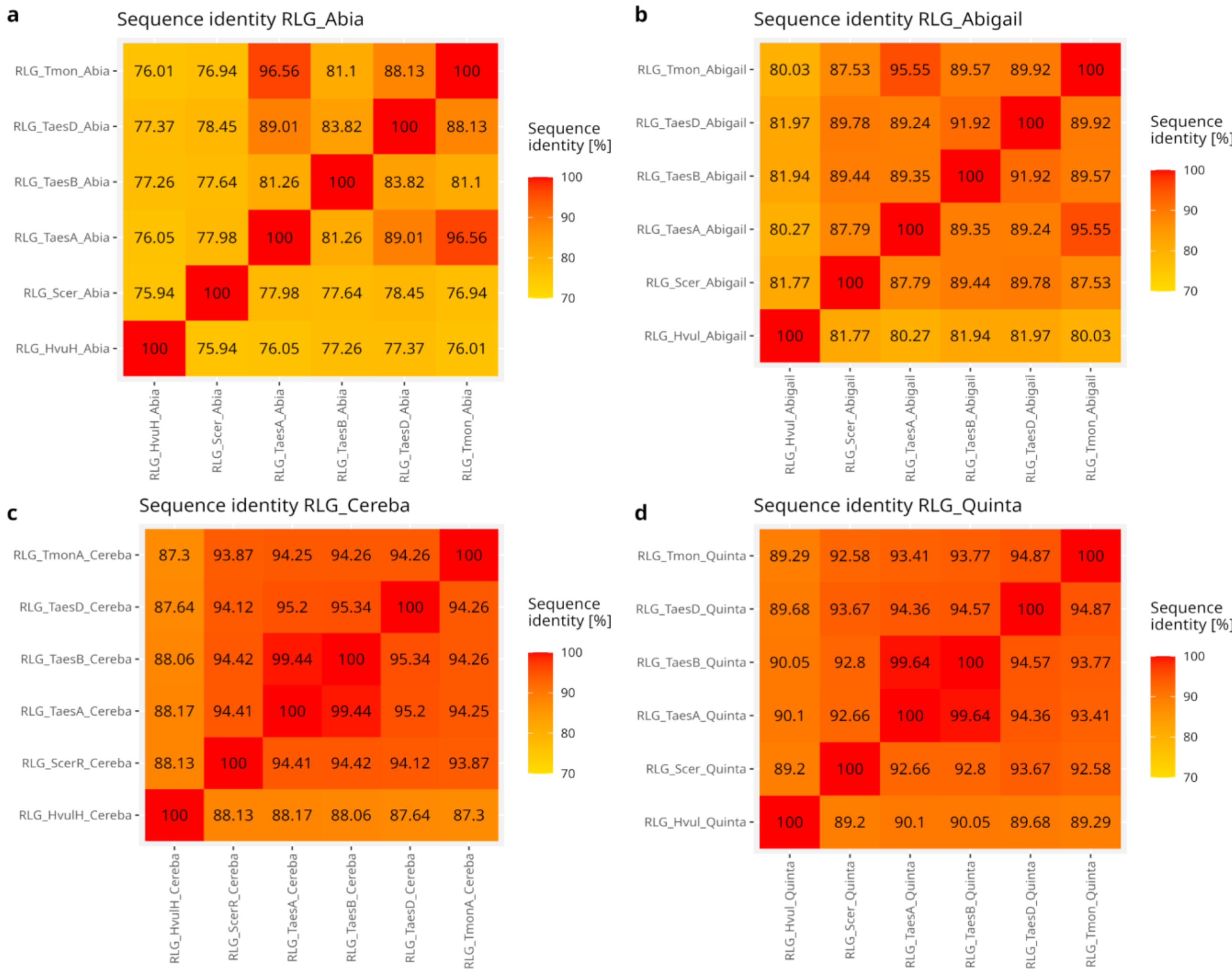

Supplementary Fig. 22. Comparison of consensus sequences of centromeric retrotransposons. The heat maps show sequence identities between consensus sequences (*RLG_Abia*, *RLG_Abigail*, *RLG_Cereba*, and *RLG_Quinta*, **a** - **d**) from rye (Scer), *T. monococcum* (Tmon), *T. aestivum* subgenome A, B and D (TaesA, TaesB and TaesD, respectively) and barley (Hvul).

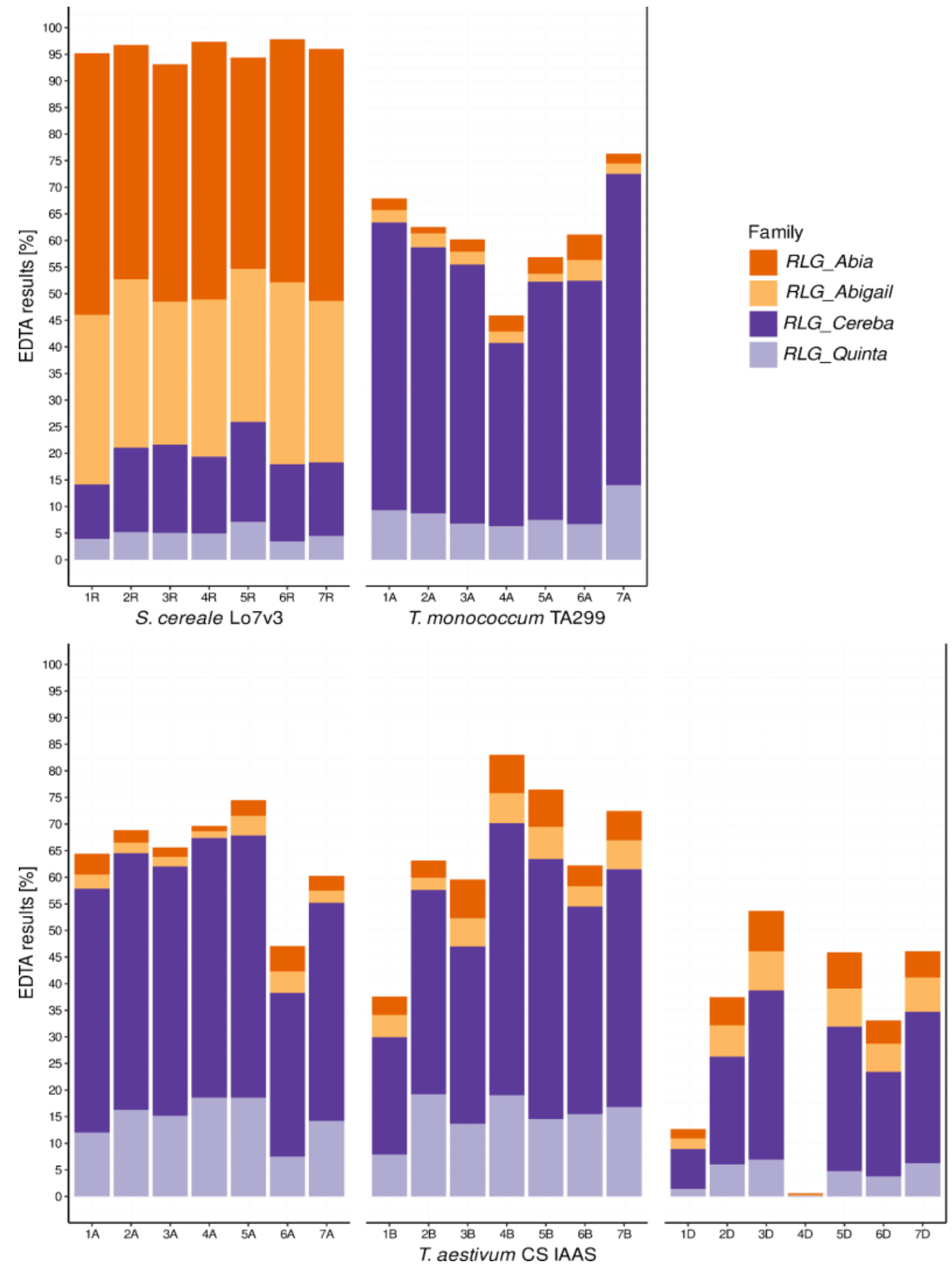

Supplementary Fig. 23. Compositional comparison of four abundant TE families in wheat and rye centromeres. EDTA results from the centromeric regions of *Triticum monococcum* TA299 (Einkorn, ENA project PRJEB61155)[5] and *Secale cereale* Lo7 (this paper) (top) as well as *Triticum aestivum* CS IAAS v1.1 (wheat, via Zenodo https://doi.org/10.5281/zenodo.10716954)[6] (bottom) per chromosome. Note that *RLG_Abia* and *RLG_Abigail* are abundant in rye centromeres in comparison to wheat and Einkorn centromeres. *RLG_Cereba* and *RLG_Quinta* on the other hand are more abundant in Einkorn and wheat centromeres in comparison to rye. The very low annotated abundance of CRM elements in the centromeres of wheat chromosome 1D (12.66%) and 4D (0.60%) is consistent with the data published alongside the CS IAAS assembly (see Supplementary Table 19 of Liu *et al.* [6]).

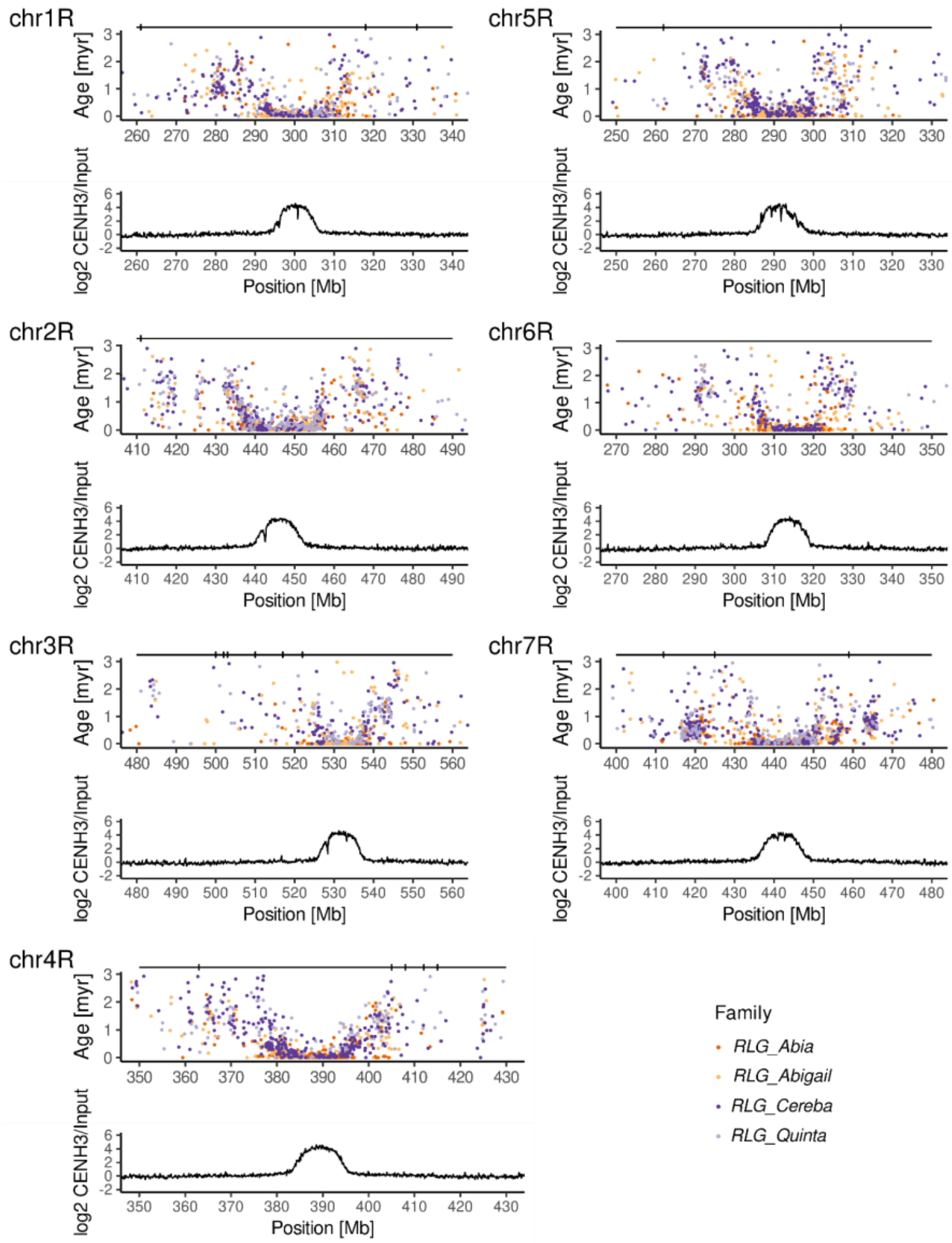

Supplementary Fig. 24. Distribution of the estimated insertion ages of copies from the retrotransposon families *RLG_Abia* (dark orange), *RLG_Abigail* (light orange), *RLG_Cereba* (dark purple) and *RLG_Quinta* (light purple) across the pericentromeric regions of all Lo7 chromosomes. Copies with an estimated insertion age of 3 million years (myrs) or younger are shown. The marks on the top track display the assembly gaps found in the respective region per chromosome. The bottom track shows the corresponding CENH3 ChIP-seq data, the ratio is displayed using a binsize of 100 kb.

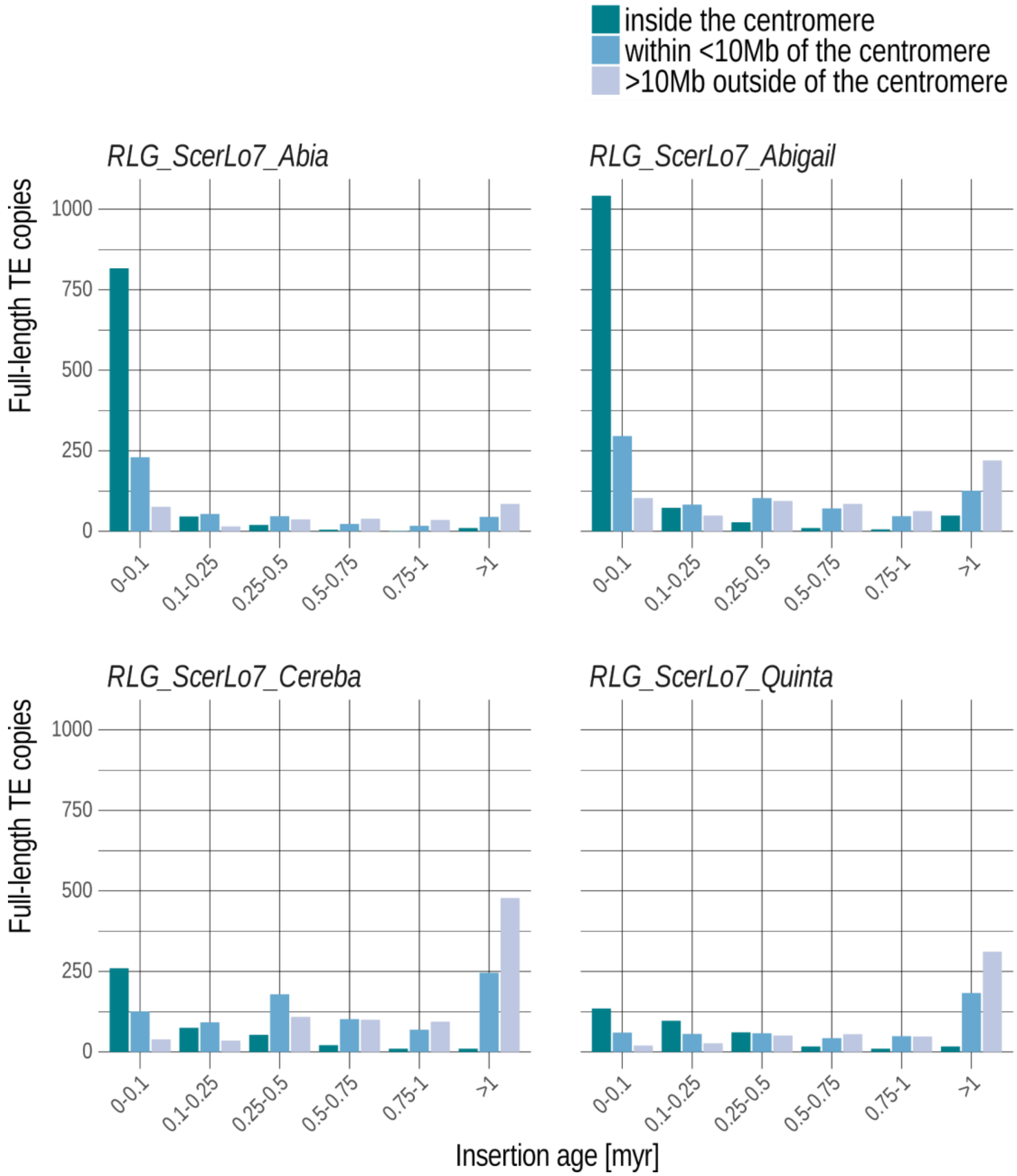

Supplementary Fig. 25. Number of full-length copies of *RLG_Abia*, *RLG_Abigail*, *RLG_Cereba* and *RLG_Quinta* across their distance to the centromere. The youngest full-length copies of the centromere specific retrotransposon families are primarily located within the functional centromere. Full-length copies with an estimated insertion age of more than 1 myr are located further from the centromere (> 10 Mb).

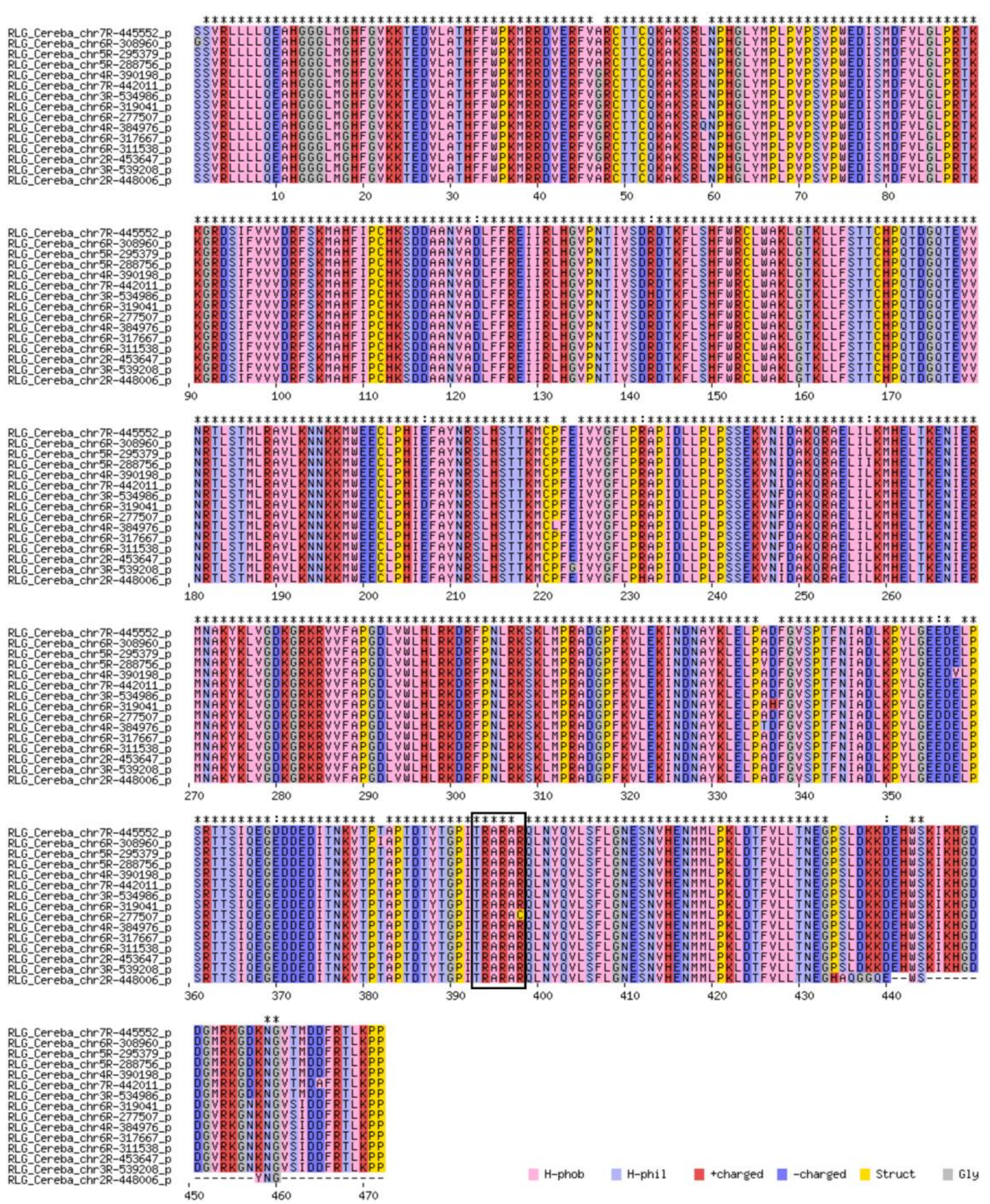

Supplementary Fig. 26. Alignment of the integrase domain (boundaries identified using NCBI conserved domains) and subsequent sequence of 15 randomly chosen copies of *RLG_Cereba* proteins with marked TRARA/K motif [7].

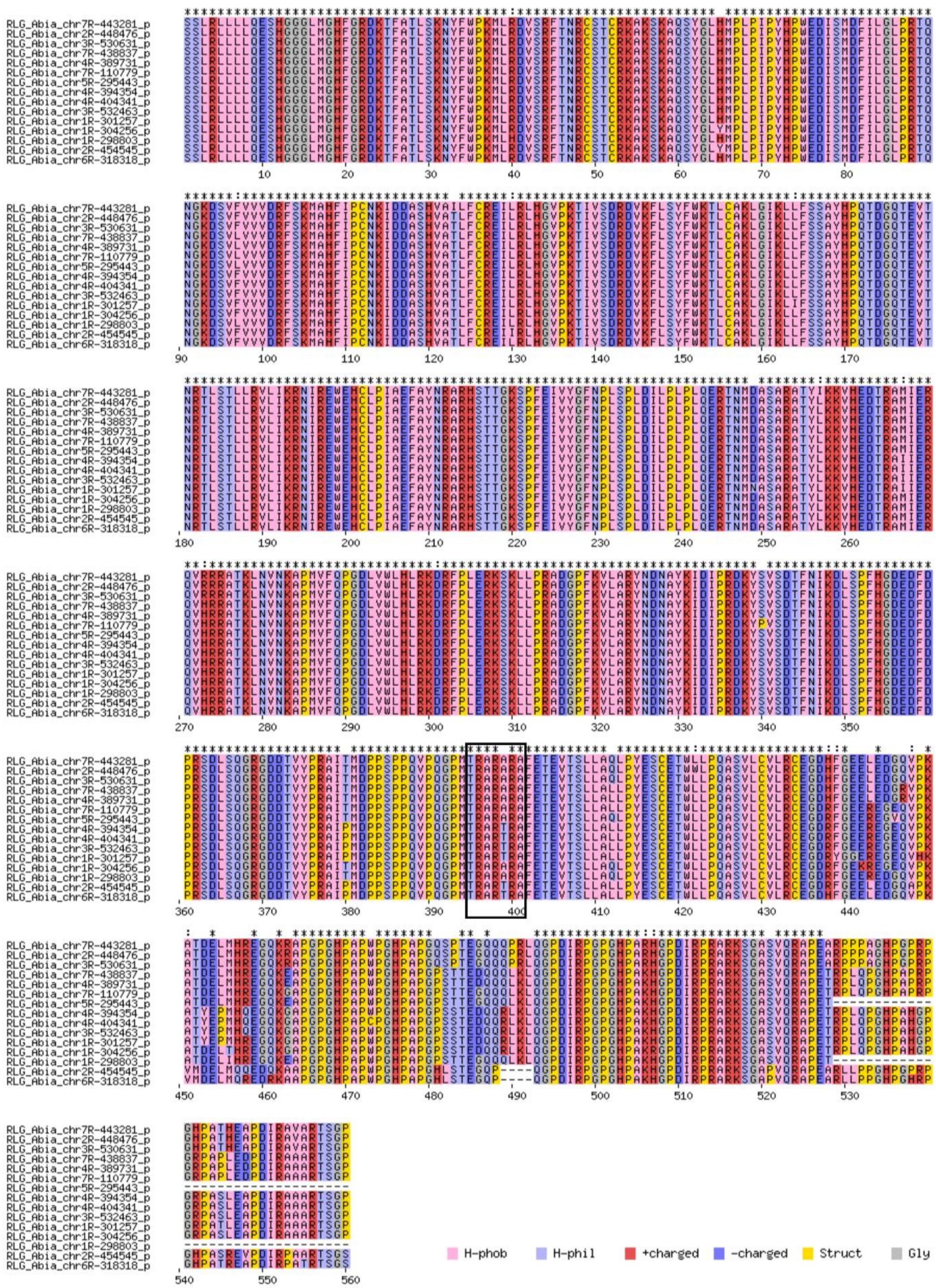

Supplementary Fig. 27. Alignment of the integrase domain (boundaries identified using NCBI conserved domains) and subsequent sequence of 15 randomly chosen copies of *RLG_Abia* proteins with marked TRARA/K motif [7].

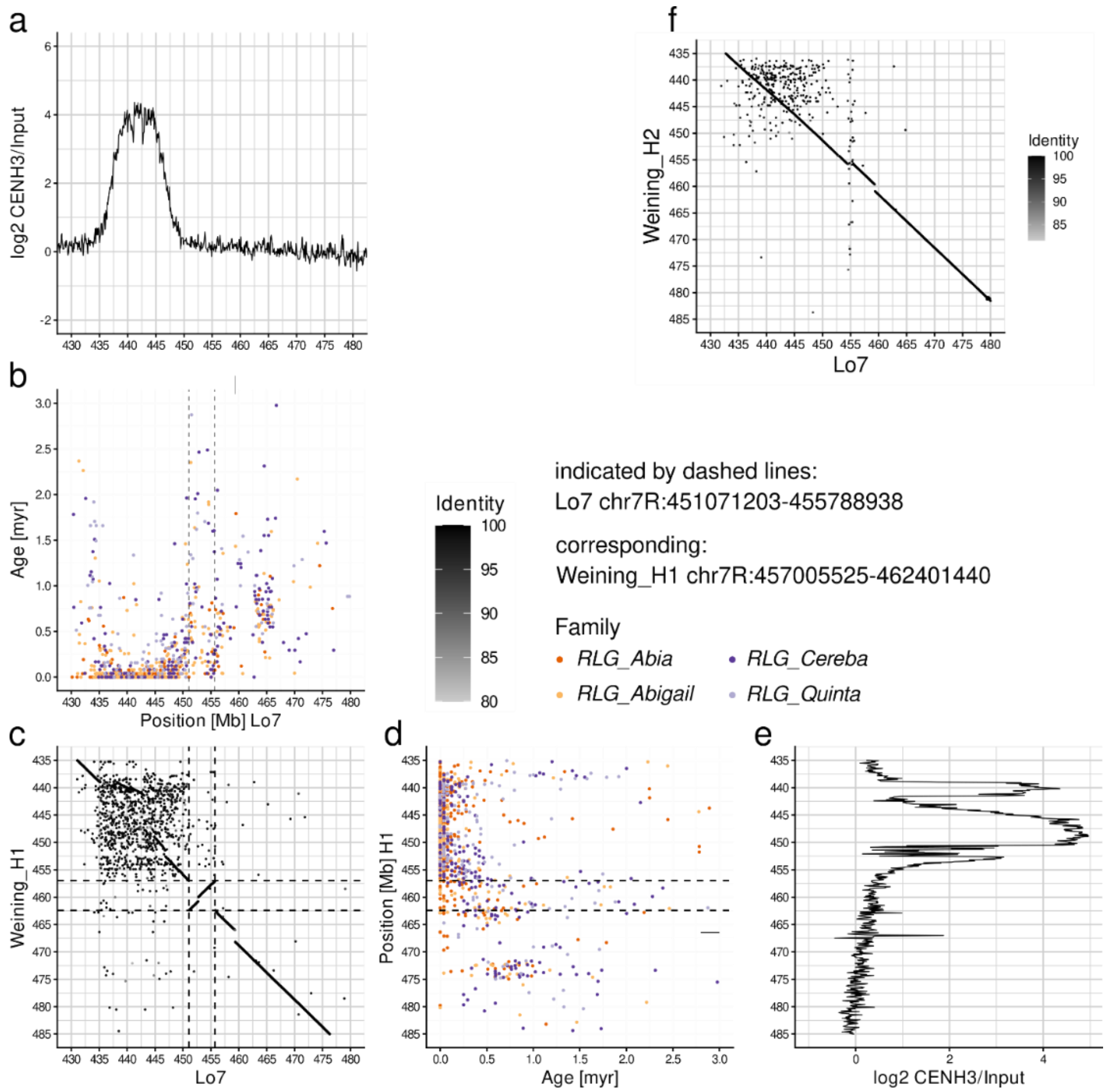

Supplementary Fig. 28. Comparison of chromosome 7R of Lo7 and Weining.V3 H1 assemblies with focus on proposed inversion C. a and **e** show the log2 ratio of CENH3/Input of Lo7 and Weining.V3 H1 respectively. **b** and **d** show the distribution of the four centromere-specific retrotransposon families *RLG_Abia*, *RLG_Abigail*, *RLG_Cereba* and *RLG_Quinta* on chromosome 7R of Lo7 and Weining.V3 H1 respectively. Gaps in the assemblies are indicated by the black strokes on the top and right side of the panels. **c** A blast comparison between the centromeric regions in Lo7 and Weining.V3 H1. The segment size was 1000 bp with a sliding step of 10 kb. Identity is indicated by the shades of gray. The black dashed lines highlight inversion C. Breakpoints in Lo7 are given based on the end of homology to Weining.V3 H1 before and the beginning of homology after the proposed inversion. **f** The same blast comparison plot for Lo7 and Weining.V3 H2.

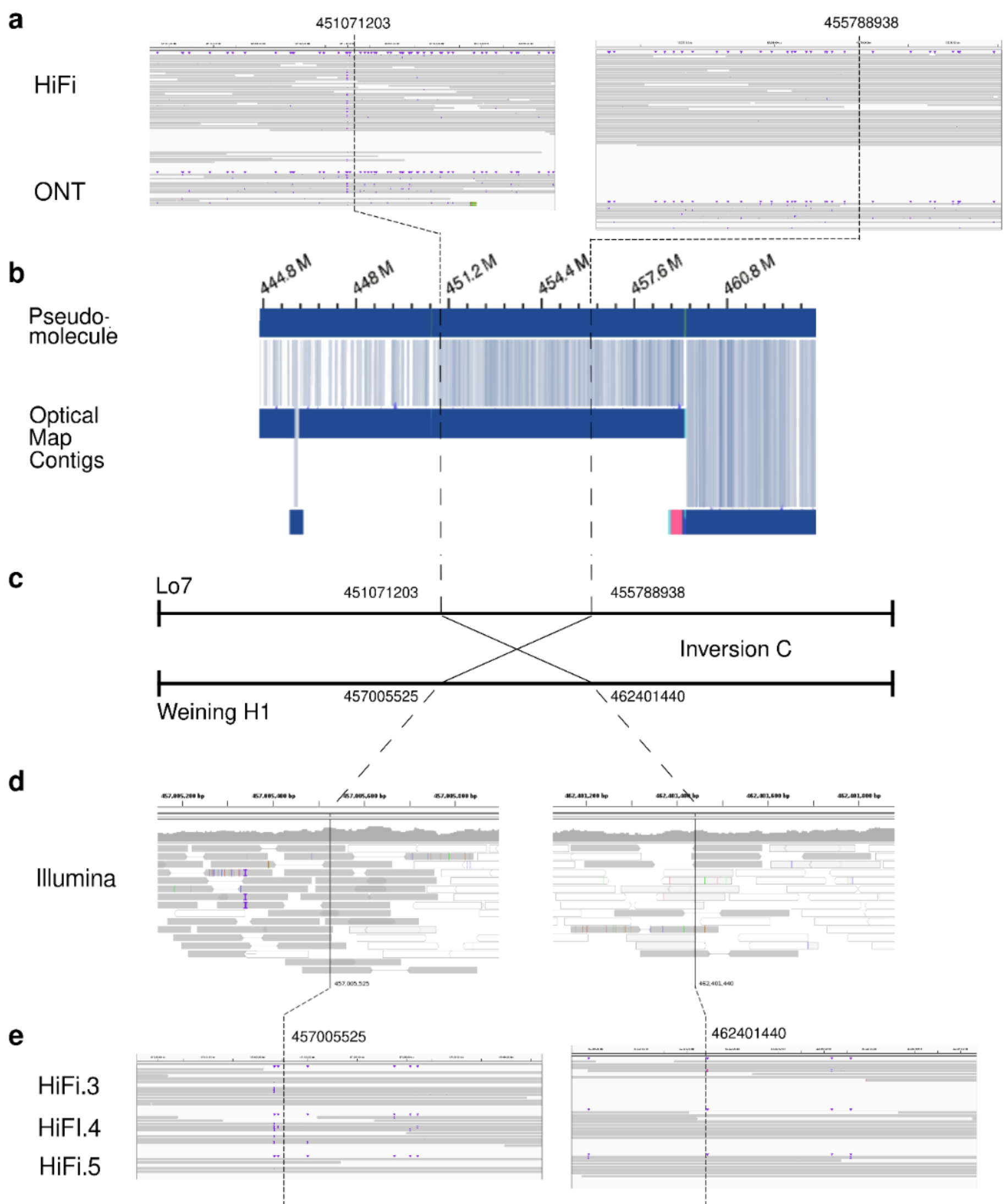

Supplementary Fig. 29. Quality assessment of genome assemblies of rye Lo7 and Weining.V3 H1 in the region of chromosomal inversion C. a PacBio HiFi and ONT reads mapped on to the region of the breakpoints of inversion C in Lo7. Precise positions of breakpoints are indicated above the map. **b** Optical map of chromosome 7 of Lo7 validating the pseudomolecule assembly. The region of inversion C is covered continuously by the optical map (indicated with vertical dashed lines). **c** Schematic representation of inversion C in Lo7 and Weining H1. **d** Mapping of Illumina short reads (Accession CRR2073116 https://ngdc.cncb.ac.cn/gsa/browse/CRA029291/CRR2073116, 40% randomly sampled using seqkit (v2.1.0) onto the phased Weining assembly V3. Note the reads and read pairs spanning the proposed breakpoints in Weining.V3 H1, confirming the assembly at the corresponding positions of the proposed Inversion C breakpoints. **e** Mapping of PacBio HiFi reads on to the region of inversion C. The breakpoints are again covered by continuous reads. Visualizations in **a**, **d** and **e** was done with the Integrative Genomics Viewer (IGV v2.12.0) software.

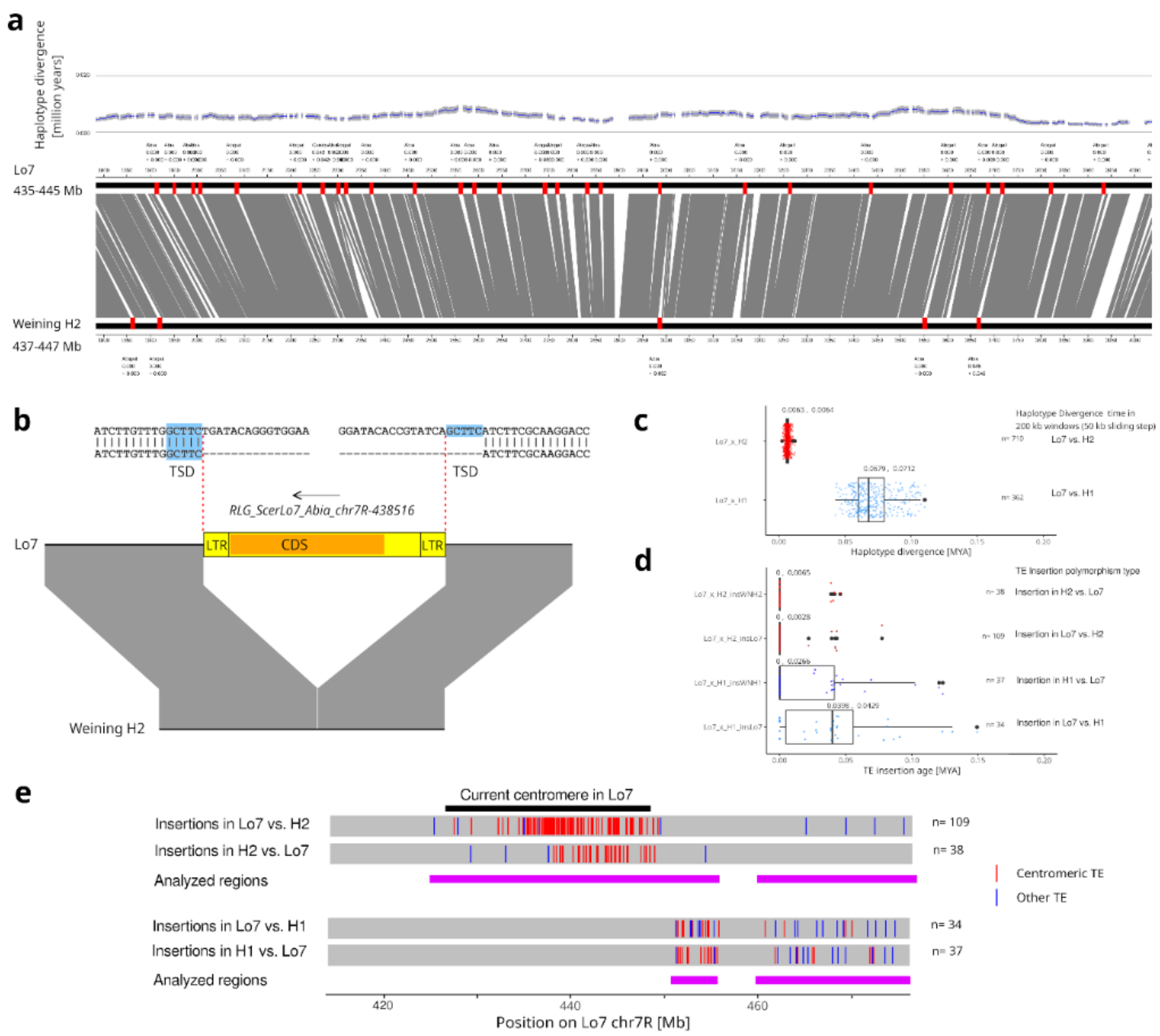

Supplementary Fig. 30. Analysis of polymorphic TE insertions between Lo7 and Weining.V3 H1 and H2, and validation of genome assembly quality. a Sequence alignment of centromeric regions from Lo7 and Weining.V3 H1. Gray shaded areas indicate regions which are conserved between Lo7 and H1. Red boxes indicate Insertions which were confirmed to be TE presence/absence polymorphisms (PAPs). For each polymorphic TE, the family name, estimated insertion age and standard deviation of estimate are given. The top track show divergence time estimates between Lo7 and H1 in sliding windows od 200 kb of conserved sequences (blue lines). Standard deviation for each window is indicated by gray bars. **b** Example of a polymorphic TE (insertion in Lo7). The insertion shows the typical 5 bp target site duplication (TSD, blue) which is created upon insertions of the TE. The two LTRs are identical which is expected for a recent insertion. **c** Box plots of individual divergence time estimates for windows of 200 kb of conserved sequences between Lo7 and H2 (top) and Lo7 and H1 (bottom). The median and average of the estimates is indicated. **d** Box plots of estimated insertion ages of TE polymorphic between Lo7 and H1 and H2. Note that the median and average insertion ages for TE PAPs between Lo7 and H2 and H1 are consistently lower than the median and average of the estimated divergence times for the respective haplotypes (except in one case: 0.0065 v.s 0.0063). **e** Positions of polymorphic TEs between Lo7 and H2 (top) and Lo7 and H1). The positions are given relative to the position in Lo7. Because the centromere region was too divergent between Lo7 and H1, it could not be aligned which is why only pericentromeric regions between the two could be analyzed. Note the high number of TE PAPs in the centromere between Lo7 and H2, which indicates that centromere-specific TEs have been active very recently (i.e. since the divergence of the two haplotypes ~ 6,300 years ago, see c.)

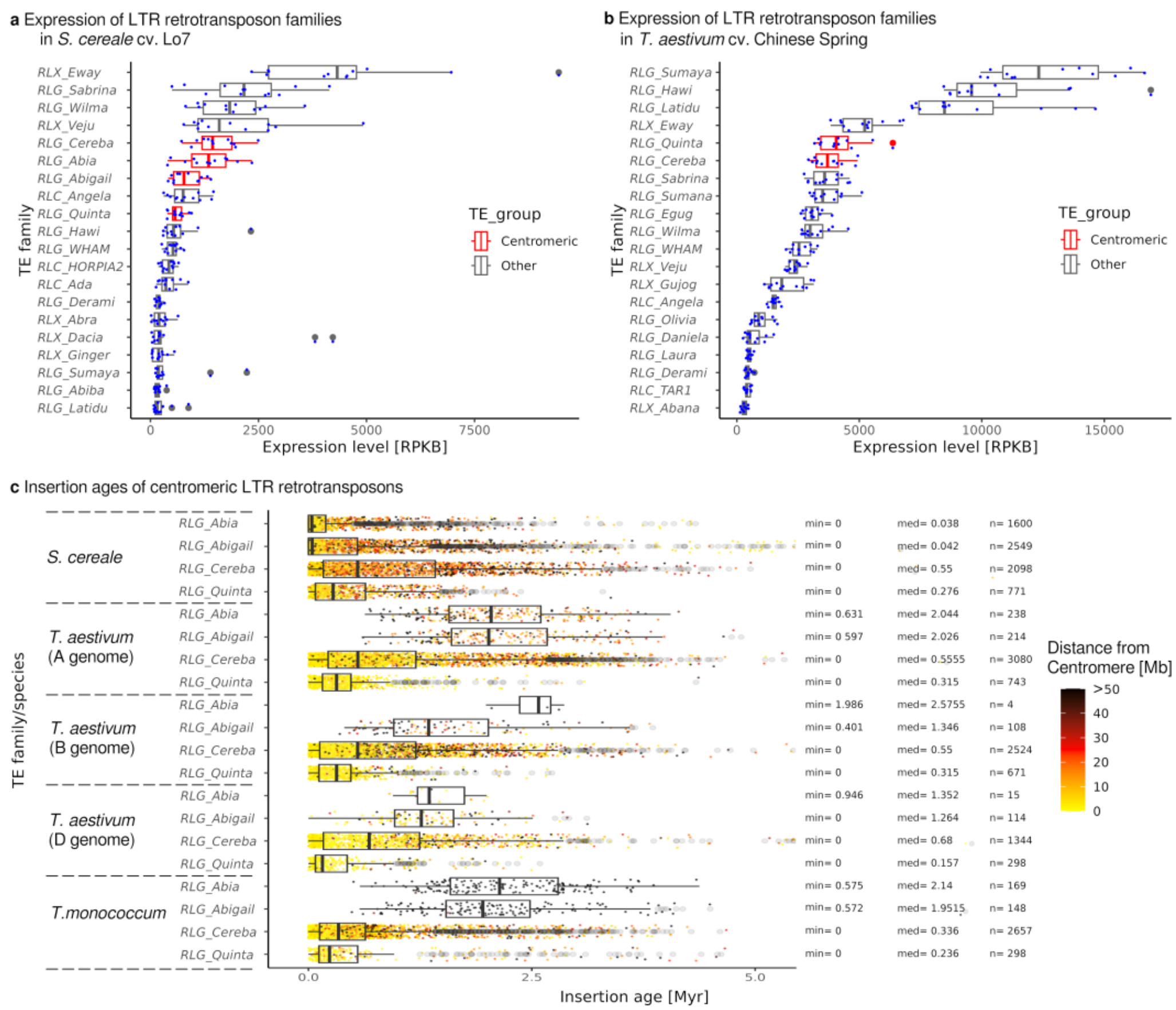

Supplementary Fig. 31. Analysis of evidence for TE activity in *S. cereale*, *T. aestivum* (bread wheat) and *T. monococcum* (Einkorn wheat). a Analysis of transcriptome data from *S. cereale* Lo7 RNAseq reads were mapped on to a TE library containing consensus sequences from rye and wheat. Only mappings in the correct transcriptional orientation were considered. Shown are the 20 LTR retrotransposon families with the highest median read counts. Centromeric retrotransposons are highlighted in red. Individual data points are shown in blue. **b** The analogous analysis with RNAseq data from *T. aestivum* cv. Chinese Spring. Note that only *RLG_Cereba* and *RLG_Quinta* were in the 20 LTR retrotransposon families with the highest median read counts. **c** Insertion age distributions of all full-length copies of centromere-specifc LTR retrotransposons identified in *S. cereale*, *T. aestivum* and *T. monococcum*. Datasets for wheat were split into individual sub-genomes. Datapoints for insertion ages of individual copies are plotted over the box blots, with the color scale indicating the distance of each copy from the predicted centromere midpoint. The columns at the right indicate insertion age of the youngest TE copy (min), median insertion age (med) and total number of TE copies (n) for each group. Note that *RLG_Abia* and *RLG_Abigail* are generally much older in wheat, indicating that they were inactive for a long time, while these two TE families have the youngest insertions in rye. In all panels, boxes indicate the inter-quartile range (IQR) with the central line indicating the median and whiskers indicating the minimum and maximum without outliers, respectively. Outliers were defined as minimum – 1.5*IQR and maximum + 1.5*IQR, respectively.

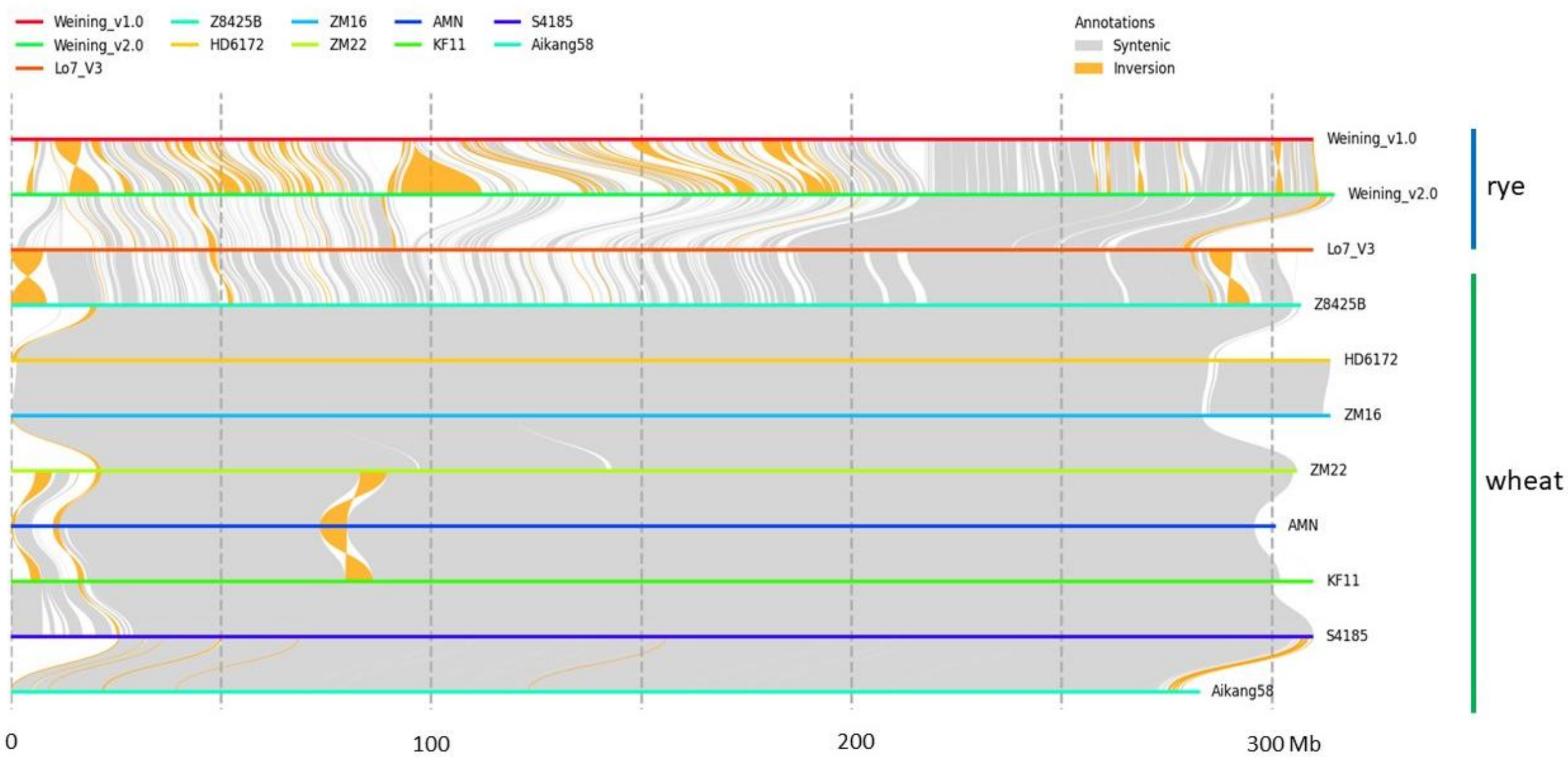

Supplementary Fig. 32. Comparative analysis of 1RS between wheat and rye accessions. Comparative analysis of the 1RS chromosome arm was conducted between two rye accessions and the 1RS arm of eight diverse wheat accessions. The analysis reveals conserved synteny as well as structural variations, highlighting the divergence and potential introgression events between species.

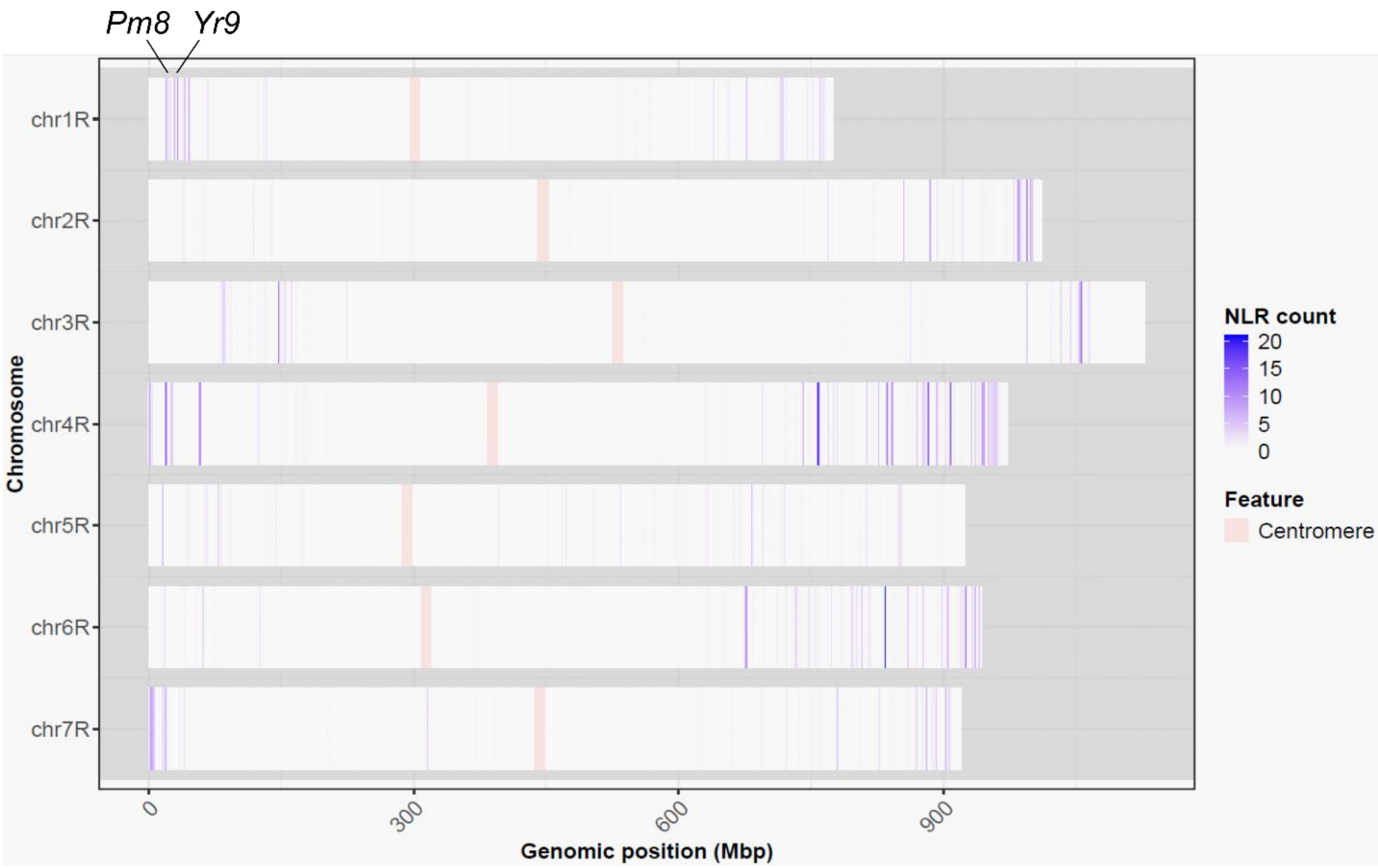

Supplementary Fig. 33. Distribution of R genes across the seven chromosomes. The number of R genes (e.g., NLRs) was calculated in non-overlapping 1 Mb genomic bins along each chromosome, using NLR-Annotator[8]. Color intensity represents the gene count per bin, ranging from low (white) to high (blue). Centromeric regions are highlighted in mistyrose. Blast resistance-associated genes, including *Pm8/S.CEREALE.LO7.r2.1RG00003910*[9] and *Yr9/S.CEREALE.LO7.r2.1RG00006720* [10,11], are located on chromosome 1R. Source data are provided as a Source Data file.

Supplementary Table 1. Accessibility of HiFi, ONT, Hi-C, ATAC-seq, and WGBS datasets.

HiFi and ONT	Sample accession
HiFi1	SAMEA118635564
HiFi2	SAMEA118635564
HiFi3	SAMEA118635564
ONT_duplexQ20_25K	SAMEA118635565

Hi-C	Sample accession	
Hi-C1_R1		Reported in Rabanus-Wallace *et al.*[12]
Hi-C1_R2		Reported in Rabanus-Wallace *et al.*[12]
Hi-C2_R1		Reported in Rabanus-Wallace *et al.*[12]
Hi-C2_R2		Reported in Rabanus-Wallace *et al.*[12]
Hi-C3_R1	SAMEA118635566	Newly generated data in this study
Hi-C3_R2	SAMEA118635566	Newly generated data in this study
Hi-C4_R1	SAMEA118635566	Newly generated data in this study
Hi-C4_R2	SAMEA118635566	Newly generated data in this study

ATAC-seq	Sample accession
A148_Lo7_rep1.1048	SAMEA118635567
A149_Lo7_rep2.1048	SAMEA118635568

WGBS	Sample accession
Lo7.WGBS.1	SAMEA121958694
Lo7.WGBS.2	SAMEA121958695
Lo7.WGBS.3	SAMEA121958696
Lo7.WGBS.4	SAMEA121958697

Raw ONT pod5 fasta	Sample accession
1	SAMEA121379501
2	SAMEA121379502
3	SAMEA121379503
4	SAMEA121379504
5	SAMEA121379505
6	SAMEA121379506
7	SAMEA121379507
8	SAMEA121379508

Supplementary Table 2. Genome size estimation of rye Lo7.

	Genome size (Mbp/1C)		
	Dolezel *et al.* [13]		Soni and Henry[14]
Lo7	7800*	7420**	7054***

* based on 1C DNA value of 3.5 pg for human standard (Tiersch *et al.* [15]), corresponding to 3,423,000,000 bp
** based on GRCh38.p12 of 3,257,319,537 bp for human standard
***recalculated estimate for the CHM13 human male genome (2C = 6.15 pg) (Nurk *et al.* [16])

Supplementary Table 3. Summary of input data and parameters of the resulting OGM assembly.

**Raw molecules filtered (>150 kbp)**	Total length	1500 Gbp
	Molecule N50	231 kbp
	Molecule coverage	213x
**OGM assembly**	No. of contigs	556
	Total length	6.69 Gbp
	Contig N50	99.6 Mbp

Quantification of 45S rDNA in Lo7 genome using optical map raw data

**Repeat statistics for units of 8.8-9.3 kb (arrays of 6+ units)**	
Total bases analysed (Mb)	1483235.79
Total bases in repeats (Mb)	3766.6
% bases in repeats	0.254
Total length of repeats in haploid genome (Mb)*	19.824

* based on Lo7 genome size of 7.06 Gb

Supplementary Table 4. Comparison of chromosome length and scaffold/contig gap between Lo7_V2 and Lo7_V3 assemblies

Chromosome	Lo7_V2 (Mb)	Lo7_V3 (Mb)	Increase (Mb)	% Increase
1R	727.34	775.56	48.22	6.6
2R	946	1011.42	65.42	6.9
3R	965.75	1128.39	162.64	16.8
4R	906.46	973.44	66.98	7.4
5R	876.15	924.46	48.31	5.5
6R	885.15	943.69	58.54	6.6
7R	899.93	920.15	20.22	2.2

Gaps

Chromosome	Lo7_V2 (Scaffold)	Lo7_V3 (Contig)
1R	71	14
2R	65	18
3R	66	24
4R	115	17
5R	84	11
6R	90	17
7R	79	5
In total	570	106

Supplementary Table 5. Comprehensive evaluation of genome assembly quality based on LAI.

Chromosome	Lo7_V2 LAI	Lo7_V3 LAI
1R	20.69	23.97
2R	21.08	24.51
3R	19.82	19.81
4R	20.19	21.75
5R	18.81	24.48
6R	19.75	25.42
7R	18.14	24.35
Average	19.78	23.47

Supplementary Table 6. Gene annotation comparison between Lo7_V2 and Lo7_V3.

All_genes	Lo7_V2	Lo7_V3
Complete BUSCOs (C)	1588, 98.4%	1597, 98.9%
Complete and single-copy BUSCOs (S)	1477, 91.5%	1135, 70.3%
Complete and duplicated BUSCOs (D)	111, 6.9%	462, 28.6%
Fragmented BUSCOs (F)	17, 1.1%	3, 0.2%
Missing BUSCOs (M)	9, 0.5%	14, 0.9%
Total BUSCO groups searched	1614	1614

HC genes_annotation	Lo7_V2	Lo7_V3
HC_chr1R	4045	5113
HC_chr2R	5049	6366
HC_chr3R	4410	5934
HC_chr4R	4837	7045
HC_chr5R	5155	6645
HC_chr6R	4457	6573
HC_chr7R	4549	5964
HC_chrUn	1939	114

HC_genes	Lo7_V2	Lo7_V3
Complete BUSCOs (C)	1546, 95.8%	1560, 96.6%
Complete and single-copy BUSCOs (S)	1446, 89.6%	1101, 68.2%
Complete and duplicated BUSCOs (D)	100, 6.2%	459, 28.4%
Fragmented BUSCOs (F)	7, 0.4%	4, 0.2%
Missing BUSCOs (M)	61, 3.8%	50, 3.2%
Total BUSCO groups searched	1614	1614

Supplementary Table 7. Phased genome assembly of Weining.V3.

	sum_len
HiFi_CCS (Gbp)	454.3
ONT (Gbp, > 25 kb)	138.5
Hi-C (Gbp)	422.4

National Genomics Data Center (NGDC), China, under BioProjectPRJCA041046

Genome assembly of Weining.V3

**Chromosome length**	**Weining.V3 H1**	**Weining.V3 H2**
chr1R (Mbp)	793.61	786.62
chr2R (Mbp)	971.81	973.33
chr3R (Mbp)	992.47	1017.28
chr4R (Mbp)	994.01	963.33
chr5R (Mbp)	909.02	894.69
chr6R (Mbp)	968.58	944.43
chr7R (Mbp)	934.7	917.79
anchored (Gbp)	6.56	6.50

Weining.V3	
anchored (Gbp)	13.06
Unanchored (Mbp)	608
In total (Gbp)	13.67

Supplementary Table 8. Phased genome assembly evaluation of Weining.V3.

LAI of Weining.V3 H1 and H2.

Chromosome	Weining.V3 H1	Weining.V3 H2
chr1R	24.24	24.88
chr2R	25.2	24.38
chr3R	24.25	22.88
chr4R	20.93	21.81
chr5R	23.62	24.82
chr6R	25.14	23.86
chr7R	24.14	24.75
Average	23.93	23.91

BUSCO of Weining.V3 H1 and H2

	Weining.V3
Complete BUSCOs (C)	1600, 99.1%
Complete and single-copy BUSCOs (S)	18, 1.1%
Complete and duplicated BUSCOs (D)	1582, 98.0%
Fragmented BUSCOs (F)	9, 0.6%
Missing BUSCOs (M)	5, 0.3%
Total BUSCO groups searched	1614

GC contents of Weining.V3 H1 and H2

	Weining.V3 H1	Weining.V3 H2
GC (%)	46.1	46.1

Supplementary Table 9. Centromere position of Weining.V3 H1 and H2.

Read mapping (MAPQ30).

Weining.V3 H1 Centromeres	Position_Sart
chr1R	301,147,091
chr2R	409,717,265
chr3R	454,166,648
chr4R	380,788,062
chr5R	274,320,526
chr6R	316,907,163
chr7R	438,949,146

Read mapping (MAPQ30)

Weining.V3 H2 Centromeres	Position_Sart	Position_End	Length
chr1R	293,253,315	304,331,999	11,078,684
chr2R	413,460,966	425,647,008	12,186,042
chr3R	465,269,971	479,781,287	14,511,316
chr4R	368,783,427	382,551,201	13,767,774
chr5R	253,503,691	267,567,947	14,064,256
chr6R	313,061,531	322,195,007	9,133,476
chr7R	436,303,155	449,909,466	13,606,311

Read mapping (MAPQ0)	Position_Sart	Position_End	Length
Weining.V3 H1 chr7R	433,454,308	465,941,162	32,486,854
Weining.V3 H2 chr7R	430,609,843	462,370,708	31,760,865

Supplementary Table 10. Quality control of CENH3 ChIP-seq short reads.

	avg_len (bp)	Q30(%)		Data source
CRR828114_f1	150	94.32	ChIP1	Liu *et al.* [17]
CRR828114_r2	150	94.07	ChIP1	Liu *et al.* [17]
CRR828115_f1	150	92.83	ChIP2	Liu *et al.* [17]
CRR828115_r2	150	91.18	ChIP2	Liu *et al.* [17]
CRR828116_f1	150	93.99	Input1	Liu *et al.* [17]
CRR828116_r2	150	93.86	Input1	Liu *et al.* [17]
CRR828117_f1	150	93.54	Input2	Liu *et al.* [17]
CRR828117_r2	150	93.05	Input2	Liu *et al.* [17]

Supplementary Table 11. Estimated sizes of the seven Lo7 rye centromeres.

Reads mapping (MAPQ30)

Centromere	Position start	Position end	Length
1R	294,894,686	305,528,973	10,634,287
2R	440,193,008	451,759,380	11,566,372
3R	525,992,185	537,137,158	11,144,973
4R	383,457,614	394,737,003	11,279,389
5R	286,328,000	295,889,973	9,561,973
6R	308,033,807	319,335,901	11,302,094
7R	436,325,765	447,617,757	11,291,992

Reads mapping (MAPQ0)

Centromere	Position start	Position end	Length
1R	289,857,897	314,916,269	25,058,372
2R	433,868,872	458,034,649	24,165,777
3R	523,258,072	540,395,139	17,137,067
4R	375,162,173	404,590,119	29,427,946
5R	278,508,655	301,385,042	22,876,387
6R	303,224,529	326,693,863	23,469,334
7R	428,672,858	459,009,684	30,336,826

Supplementary Table 12. Comparison of inversion number, maximum size, and average size between rye and wheat accessions.

	Weining_v2.0 vs. Lo7_V3	Lo7_V3 vs. Z8425B	Z8425B vs. HD6172
Num_Inversion	20	29	1
Max_Inversion	1,368,334	7,806,654	1,473,975
Avg_Inversion	198,588	534,159	1,473,975

Supplementary references

1 Ranallo-Benavidez, T. R., Jaron, K. S. & Schatz, M. C. GenomeScope 2.0 and Smudgeplot for reference-free profiling of polyploid genomes. *Nat. Commun.* **11**, 1432 (2020).

2 Goel, M., Sun, H., Jiao, W. B. & Schneeberger, K. SyRI: finding genomic rearrangements and local sequence differences from whole-genome assemblies. *Genome Biol.* **20**, 277 (2019).

3 Li, G. et al. A high-quality genome assembly highlights rye genomic characteristics and agronomically important genes. *Nat. Genet.* **53**, 574-584 (2021).

4 Yi, C. et al. High-resolution genome assembly reveals retrotransposon-mediated centromere dynamics in rye. *Genome Biol.* **26**, 308 (2025).

5 Ahmed, H. I. et al. Einkorn genomics sheds light on history of the oldest domesticated wheat. *Nature* **620**, 830–838 (2023).

6 Liu, S. et al. A telomere-to-telomere genome assembly coupled with multi-omic data provides insights into the evolution of hexaploid bread wheat. *Nat. Genet.* **57**, 1008–1020 (2025).

7 Heuberger, M. et al. Evolution of Einkorn wheat centromeres is driven by the mutualistic interplay of two LTR retrotransposons. *Mob. DNA* **15**, 16 (2024).

8 Steuernagel, B. et al. The NLR-Annotator Tool Enables Annotation of the Intracellular Immune Receptor Repertoire. *Plant Physiol.* **183**, 468-482 (2020).

9 Hurni, S. et al. Rye *Pm8* and wheat *Pm3* are orthologous genes and show evolutionary conservation of resistance function against powdery mildew. *Plant J.* **76**, 957-969 (2013).

10 Yu, Y. et al. Wheat stripe rust resistance gene *Yr9*, derived from rye, is a CC-NBS-LRR gene in a highly conserved NLR cluster. *Sci. China Life Sci.* **68**, 2807-2809 (2025).

11 Wang, C. et al. The *Yr9* gene encoding a CC-NBS-LRR protein in the 1RS-1BL translocation confers wheat stripe rust resistance. *Sci. China Life Sci*. **68**, 2804–2806 (2025).

12 Rabanus-Wallace, M. T. et al. Chromosome-scale genome assembly provides insights into rye biology, evolution and agronomic potential. *Nat. Genet.* **53**, 564-573 (2021).

13 Dolezel, J., Bartos, J., Voglmayr, H. & Greilhuber, J. Letter to the editor. *Cytometry A* **51**, 127-128 (2003).

14 Soni, A. & Henry, R. J. Re-calibration of flow cytometry standards for plant genome size estimation. *Front. Plant. Sci.* **16**, 1548766 (2025).

15 Tiersch, T. R., Chandler, R. W., Wachtel, S. S. & Elias, S. Reference standards for flow cytometry and application in comparative studies of nuclear DNA content. *Cytometry* **10**, 706-710 (1989).

16 Nurk, S. et al. The complete sequence of a human genome. *Science* **376**, 44-53 (2022).

17 Liu, C. et al. Young retrotransposons and non-B DNA structures promote the establishment of dominant rye centromere in the 1RS.1BL fused centromere. *New Phytol.* **241**, 607-622 (2024).
