# Supplementary material for: Unveiling centromeric retrotransposon dynamics through a high-quality rye genome assembly
Source: Nat Commun. 2026 Aug 15;17:8413. doi: 10.1038/s41467-026-76753-4 (PMC13477480; doi:10.1038/s41467-026-76753-4)
Supplement: Supplementary file 3 [file 41467_2026_76753_MOESM2_ESM.pdf]

nature portfolio

Peer Review File

Unveiling centromeric retrotransposon dynamics through a high-quality rye genome assembly

Corresponding Author: Professor Nils Stein

This file contains all reviewer reports in order by version, followed by all author rebuttals in order by version.

Version 0:

Reviewer comments:

Reviewer #1

(Remarks to the Author)
This manuscript presents an improved chromosome-scale genome assembly (Lo7_V3) for the inbred rye line Lo7, leveraging PacBio HiFi, ONT, Hi-C, and BioNano technologies. The authors highlight advancements in contiguity, centromere assembly, and annotation over previous versions (Lo7_V1/V2), alongside analyses of centromeric retrotransposon dynamics and 1RS translocation diversity in wheat-rye hybrids. While the resource is valuable for the community, the study lacks novelty relative to recent rye genome publications (e.g., Rabanus-Wallace et al. and Li et al., Nat Genet 2021) and suffers from critical methodological omissions. Key claims—including centromere "shifts" and species-specific TE activity—are inadequately supported, and technical flaws (e.g., low sequencing depth, incomplete benchmarking) undermine confidence in the assembly. Overall, the work falls short of the standards expected for publication in Nature Communications and requires major revisions to address the following concerns.

Major revisions:
1. Insufficient assembly quality assessment, the reliance on LAI (LTR Assembly Index) alone is inadequate for evaluating assembly completeness and accuracy. Standard metrics like BUSCO scores (for gene space), Merqury QV (consensus accuracy), and coverage uniformity of HiFi/ONT reads mapped to the assembly must be included. These omissions obscure potential biases in repetitive regions and undermine claims of "high quality."
2. The sequencing depth, particularly for Hi-C data (20X), is insufficient for a genome as complex as rye—characterized by outcrossing behavior, high heterozygosity, large size, and extensive repeats. Hi-C data at this depth may fail to resolve chromosomal interactions in repetitive regions, leading to misassemblies or incomplete scaffolding. The authors must justify why 20X Hi-C is adequate for their purposes or provide additional Hi-C data to support the chromosome-scale assembly.
3. The updated gene annotation (75,850 genes) lacks mapping to previous Lo7_V2 genes. Without clear correspondence (e.g., shared identifiers or synteny analysis), transitioning between versions is impractical for end users. A gene liftover track or comparative table is essential.
4. The reported genome size of Lo7_V3 (6.76 Gb) is substantially smaller than the ~7.9 Gb estimated for rye in two 2021 Nature Genetics studies, including a flow cytometry-based measurement for Weining rye. This discrepancy raises concerns about potential assembly gaps or underrepresentation of repetitive regions. The authors must investigate and explicitly explain this difference—whether it stems from assembly errors, insufficient sequencing depth, biological variation between accessions, or other factors—and provide evidence to support their explanation.
5. The claim of multiple centromere shifts on chromosome 7R, inferred from TE insertion patterns, is inadequately validated. The authors rely solely on TE age and distribution, without confirming these shifts using Hi-C interaction data (e.g., changes in contact intensity at proposed inversion breakpoints) or synteny analyses with other rye accessions. Without such validation, these shifts could be artifacts of assembly errors. The authors must strengthen this claim with orthogonal evidence, including structural variant calls from Hi-C and comparative analyses with related genomes.
6. The conclusion that RLG_Abia is "highly active" in rye but suppressed in wheat overlooks technical confounders. The superior continuity of Lo7_V3 likely enables more complete TE annotation, whereas fragmented wheat assemblies may miss elements. To assert biological divergence, the authors must reannotate a high-quality wheat genome (e.g., CS_T2T) using their pipeline and statistically compare TE abundances.
7. Additionally, The authors do not quantify the accuracy of repeat annotations (e.g., consistency of TE family classification with Lo7_V2) or explain the root causes of errors in Lo7_V2 (e.g., 1R inversions). Without evaluating potential biases in assembly tools (e.g., short-read assemblers), it is unclear whether improvements stem from technological advances or parameter optimization.

Reviewer #2

(Remarks to the Author)
Rye is an important cereal crop within the tribe Triticeae, which poses unique challenges for genome assembly due to its large genome, high repeat contents, and extensive structural heterogeneity. In this study, the authors present a chromosome-scale, near-complete genome assembly for rye inbred variety Lo7, utilizing PacBio HiFi, Oxford Nanopore, Hi-C, and BioNano sequencing and TRITEX pipeline. Compared with previous versions, the assembly further improved the resolution of complex repetitive regions in subtelomeres and centromeres. This work makes rye more accessible to genomic research and a valuable resource that will support future genomic research and crop improvement efforts in rye and related cereal species.
Overall, the logic is good, the analyses are clear and data are reliable. Here are some comments:

1. Some errors/typos in the manuscript, either from the text or from the figures, need to be carefully checked and revised. Here are only several examples.
(1) Please ensure consistent decimal precision for numerical values throughout the manuscript, such as ~0.6 million years ago (Line 219), 416 Mb (Line 220), 0.25 mya (Line 221) and so on. Please carefully review the entire text.
(2) Some specialized terms should be italicized, such as the names of species (e.g., in reference 23) and genes (e.g., in in reference 4). Authors are kindly requested to carefully review the entire text.
2. The chloroplast sequences were removed from the final rye genome. Were the mitochondrial genome sequences removed from the final rye genome as well? Please provide an explanation in the Methods section.
3. Compared to previously published rye genomes, how many additional genes were identified in the new assembly, and how is the gene prediction accuracy? What are the methodological criteria for distinguishing high-confidence versus low-confidence genes in the annotation pipeline? Please provide an explanation in the Methods section.
4. Please provide the accession numbers (e.g., NCBI/ENA/DDBJ) of all released datasets associated with this study in the data availability section.
5. T2T genome assemblies of crops have significantly expanded our understanding of genome architecture and function. In the Discussion section, please discuss the potential strategies to advance this rye genome assembly to T2T level in the future.

Reviewer #3

(Remarks to the Author)
This manuscript presents an updated assembly of Rye Lo7 genome. The authors have used that latest PacBio HiFi, ONT and Hi-C to create a much more contiguous assembly than previously available, which fully resolves the centromeric sequences and provides an useful updated resource for the field. It highlights the role of Gypsy superfamily transposons which form the majority of the centromere sequences and use the updated assembly to investigate the sequence diversity of wheat/rye 1RS. Overall, I consider the manuscript of excellent quality and well written.

I only have a couple of comments:

The manuscript could benefit from a more extensive discussion on the evolutionary implications of centromeric retrotransposon dynamics in other cereal genomes. In the centromere TEs found RLG_Cereba, RLG_Quinta and RLG_Abia, a comparative analysis how similar are these TEs in structure to the RLG_Cereba and RLG_Quinta found in closely related species such as wheat or barley, would enhance evolutionary context.

Additionally, is there any specific mutations or domain structures in these elements that are specific to the centromere clades that could explain the involvement in centromere function? Such as those recently reported (Tsukahara, S., Bousios, A., Perez-Roman, E. et al. Centrophilic retrotransposon integration via CENH3 chromatin in Arabidopsis. Nature 637, 744–748 (2025). https://doi.org/10.1038/s41586-024-08319-7)

Activity and Dynamics of RLG_Abia (Lines 197-198): Is there any evidence in transcriptome data that the RLG_Abia retrotransposons are actively transcribed? Is this continuing ongoing integration at the centromere or a burst of centromeric integration creating a younger region in whihch the functional centromere shifts?

In the methylation profiling only CG context reported, however all contexts could be called using the Nanopore data which would enhance the manuscript – Particularly it would be interesting to see this profile over the centromere regions which often have a centromeric dip region in CG or CHG context methylation

Minor:
In Fig 2 a comparative plot between the old and new assembly would be beneficial to compare the updated sequence - like the syri plot in Supplementary Figure 13

Version 1:

Reviewer comments:

Reviewer #1

(Remarks to the Author)
Although the authors have addressed several technical concerns raised in the first round and improved the assembly metrics with additional quality control (Merqury QV, BUSCO, coverage distribution), the revised manuscript remains primarily incremental. The study reports an upgraded genome assembly (Lo7_V3) and descriptive analyses of centromeric retrotransposons but fails to deliver substantive biological novelty or mechanistic insight. The revisions mainly add data rather than resolve the conceptual issues previously noted.
Major reasons for rejection:
1. Limited conceptual advance and biological novelty. The new version continues to focus on technical improvement—longer contigs, fewer gaps, and slightly enhanced annotation accuracy—without introducing a new biological discovery. The main results, such as the completion of seven centromeres and the high abundance of RLG_Abia/RLG_Abigail elements, are descriptive in nature. There is no experimental or computational evidence explaining how these transposons influence centromere function, structure, or evolution. The work therefore reads as a genome-resource update rather than a research paper with conceptual impact suitable for Nature Communications.
2. Insufficient validation of the proposed centromere shifts on chromosome 7R. The authors added additional analyses to support their model of three consecutive inversions on 7R, including dotplot comparisons with Lo225, Hi-C contact maps, and optical map consistency checks. However, these additions remain insufficient to substantiate the claim of multiple centromere shifts. The interpretation is still derived mainly from the distribution and estimated ages of centromeric retrotransposons, which represent indirect correlations rather than structural evidence. No independent breakpoint-level validation, statistical support, or reproducible synteny analysis across multiple published rye genomes is provided. Without explicit sequence-based inversion calls and public access to the Lo225 data, this claim remains speculative and does not meet the evidentiary standard for a high-impact journal.

3.Assembly completeness and data–claim mismatch. The authors emphasize that all seven centromeres are “fully assembled,” yet only 6 of 14 telomeres were detected and 106 gaps remain unresolved. The final assembly size (6.76 Gb) is substantially smaller than cytometric estimates of ~7–8 Gb. The explanations provided—such as differences in standard genome size calibration or the collapse of STRs—are qualitative and unsupported by quantitative breakdowns of missing sequence classes. Similarly, the justification for the adequacy of ~20× Hi-C depth in a 7 Gb repeat-rich genome is based on empirical experience rather than any quantitative modeling or curve-fitting analysis demonstrating coverage sufficiency across repetitive regions. As a result, the claim of a “near-complete” or “complete centromere” assembly overstates what is currently achieved.

4. Questionable interpretation of RLG_Abia activity differences between rye and wheat. The authors reannotated TEs using a unified library and added RNA-seq expression comparisons between rye and wheat. Nevertheless, the conclusion that RLG_Abia and RLG_Abigail are “highly active” in rye but “suppressed” in wheat is still weakly supported. The analysis remains vulnerable to annotation bias, inconsistent RNA-seq datasets, and lack of population-level validation. No transposition polymorphism (TIP) evidence, recent insertion signatures, or molecular intermediates are provided to demonstrate genuine activity. Hence, the observed differences could simply reflect assembly and annotation quality rather than true biological divergence.

In summary, while the revised manuscript presents an improved genome assembly, it does not resolve the central conceptual and evidential issues identified previously. The work remains incremental, with key claims inadequately supported by independent validation. I therefore recommend rejection of this manuscript for Nature Communications. The dataset could be more appropriately published as a technical resource after further validation and expansion of biological insight.

Reviewer #2

(Remarks to the Author)
The authors have properly addressed all my concerns, and I have no further questions.

Reviewer #3

(Remarks to the Author)
I thank the authors for their revisions and for their response to my comments. I have reviewed the revised manuscript and find it substantially improved, with most of my previous comments addressed. I have only a small number of remaining points:

1. Line 180: The authors should clarify what is meant by the term “functional” centromere here. Does this refer specifically to the CENH3-enriched region (spanning approximately 10–12 Mb, Supplementary Table 8), or is this being conflated with the full centromeric retrotransposon array? A 10–12 Mb region seems quite large for a functional centromere, even in comparison to large genomes like wheat.

Looking at the CENH3 analysis it is not stated whether the ChIP-seq mapping includes multi-mapping reads. If multi-

mappers are included, this could substantially inflate the apparent size of the centromeric domain due to the repetitiveness of the TEs. I would recommend estimating upper and lower bounds of centromere size using two mapping strategies:
1) multiple + unique mapping to provide a maximum (upper bound) estimate this is likely the reported 10-12Mb, and
2) unique-only mapping only to provide a minimum (lower bound) estimate.
This approach has been used successfully in other species (e.g. https://doi.org/10.1111/tpj.70153, https://doi.org/10.1371/journal.pgen.1000743) to provide a range on which the likely functional centromere occupies

2. Nanopore does provide a tool for calling methylated cytosines, and this can be done through Dorado basecaller using R10 data their integrated modified base models (see below). While I understand that the assembly subset (>25 kb reads) may only provide ~9× coverage, relaxing this threshold to >5–10 kb would likely yield higher coverage sufficient to generate a useful genome-wide methylation profile, which would be a valuable addition to the manuscript.

If this still doesn't provide sufficient coverage for this analysis, the basecalling should be performed using the dorado dna_r10.4.1_e8.2_400bps_sup@v5.2.0 with the 5mC_5hmC modified base model, and that the resulting BAM file can provided to the community. This is important, because methylation cannot be re-called at a later date as the authors suggested without the pod5 files, which are not currently available through the ENA under project ID PRJEB91463 (based on the methods the current provided .bam file wont contain the methylation tags for the community).

Version 2:

Reviewer comments:

Reviewer #1

(Remarks to the Author)
The authors have clearly invested considerable effort in improving the manuscript. The additional analyses have improved the manuscript, especially with respect to assembly validation, re-analysis of centromeric regions, and the comparison with the Weining datasets. I appreciate the amount of work that went into the revision.

That said, after reading the revised manuscript and rebuttal carefully, I remain unconvinced that the main concerns have been fully resolved. In my view, the study is still centered primarily on an improved rye genome assembly and a more detailed description of centromeric retrotransposons, but it does not yet provide enough conceptual or mechanistic advance to justify publication in Nature Communications.

My main concerns are the following.

First, the overall biological advance remains limited. The revised manuscript now provides a stronger assembly resource and a richer description of centromeric TE composition and distribution, but the interpretation is still largely descriptive. In particular, the study does not yet establish clearly how the observed TE patterns translate into a deeper mechanistic understanding of centromere function, centromere repositioning, or chromosome evolution. For this reason, the manuscript still reads more like a resource and comparative genomics study than a paper with a clear conceptual advance.

Second, the evidence for the proposed 7R model remains uneven. The additional comparison with the phased Weining reassembly strengthens the case for the most recent event, inversion C, and provides more convincing support through approximate breakpoint definition and read-based validation. However, the older proposed events are still inferred mainly from TE age-position patterns rather than from comparable structural evidence. The rebuttal itself acknowledges that exact breakpoints cannot be provided for these older events, yet the Results section still presents a relatively strong three-inversion scenario with temporal assignments. I think the current data support the conclusion that the peri-centromeric region of 7R has undergone structural rearrangement, with one recent event being relatively well supported, but the full three-step model remains at least partly speculative.

Third, some of the wording in the revised manuscript is still stronger than the data support. This is particularly noticeable in the discussion of RLG_Abia and RLG_Abigail activity. In the rebuttal, the authors state that they toned down the language and that there is no evidence for recent activity in wheat. However, the manuscript still states that these families were "silent for a long time" in wheat and are "currently highly active" in rye. This goes beyond the more limited conclusion that recent activity is supported in rye but not demonstrated in wheat. I think this section should be phrased more cautiously.

Fourth, I am concerned by inconsistencies between the rebuttal and the revised manuscript in the reporting of the TE insertion polymorphism results. The numerical summaries do not appear fully consistent, and part of the rebuttal summary also seems internally inconsistent. These issues may reflect revision-stage updating, but they should be reconciled clearly, as they weaken confidence in an analysis that is now central to the revised argument.

Fifth, the discussion of the published Weining.V2 assembly should be more balanced. I agree that unresolved haplotypic redundancy in a more heterozygous assembly may complicate direct fine-scale comparison with the highly homozygous Lo7 assembly, and I understand why the authors generated a phased reassembly for this purpose. However, the current manuscript tends to treat this as evidence of broadly reduced assembly quality or structural flaws in the published Weining.V2 assembly. In my view, the analyses presented here support the conclusion that Weining.V2 is not ideal for this specific benchmarking purpose, but they do not support a broader assessment of its overall quality. This distinction should be made more clearly.

Sixth, the completeness issue is still not fully addressed quantitatively. The revised flow-cytometric estimate is useful and does make the reported assembly size more plausible. However, the remaining discrepancy is still explained mainly in qualitative terms, for example by invoking subtelomeric tandem repeats and repeat-rich unresolved regions. In addition, Supplementary Fig. 5 appears to show several localized windows with elevated depth in both HiFi and ONT data despite relatively stable GC content. This pattern could be consistent with local collapsed or over-compressed repetitive sequence representation, yet these outlier regions are not quantified or discussed in detail. It would be important to estimate their genomic span, determine whether they are enriched for particular repeat classes, and assess how much of the remaining assembly-length discrepancy they may explain.

Finally, the Discussion still goes somewhat beyond what is directly supported by the data. For example, some statements are framed in terms of a broader retrotransposon-mediated mechanism of centromere evolution or long-term competition for the centromeric niche. These are interesting ideas, but I do not think the present analyses support them strongly enough to present them as conclusions.

Overall, the revision improves the manuscript technically, but I do not think it fully resolves the main concerns regarding conceptual advance and support for the strongest claims. For these reasons, I do not think the manuscript reaches the threshold for publication in Nature Communications.

Version 3:

Reviewer comments:

Reviewer #1

(Remarks to the Author)
I have read the revised manuscript and the rebuttal carefully. The authors have addressed several technical points. The assembly validation is clearer, the comparison with the Weining datasets is more useful, and the revised analysis of the centromeric regions is improved.

However, my main concern remains. The revision has not substantially changed the nature of the study. The manuscript still reads primarily as an improved rye genome assembly, with a descriptive survey of centromeric retrotransposon composition, distribution, and insertion-age patterns. These are useful data, but I do not think they amount to a sufficiently clear conceptual advance for Nature Communications.

The strongest biological claim concerns centromere-associated structural evolution, especially the proposed three-step inversion model on chromosome 7R. The additional Weining comparison strengthens the case for the most recent event, inversion C. In contrast, the older proposed events are still inferred mainly from TE age-position patterns rather than from comparable structural evidence. The current data support the conclusion that the 7R pericentromeric region has undergone structural rearrangement, but I do not think they support the full three-inversion model with the confidence and temporal resolution suggested in the manuscript.

I also remain concerned that some statements are still stronger than the data justify. For example, the discussion of RLG_Abia and RLG_Abigail would be better framed as evidence for recent accumulation and possible ongoing activity in rye, rather than as direct evidence that these elements are currently highly active. Similarly, the lack of comparable recent insertions in wheat should not be presented too strongly as long-term silence without additional supporting evidence.

The completeness of the assembly also limits the resource argument. The assembly is clearly improved, but it is not telomere-to-telomere, several subtelomeric regions remain unresolved, and the authors still report an assembly-size discrepancy consistent with collapsed or unresolved repeat-rich regions. For a resource-centered manuscript at this journal level, either the assembly would need to be exceptionally complete, or the biological conclusions drawn from it would need to be substantially stronger.

Finally, parts of the Discussion still go beyond what is directly demonstrated. The proposed retrotransposon-mediated mechanism of centromere evolution and the idea of long-term competition for the centromeric niche are interesting hypotheses, but the current study does not establish them as conclusions.

Overall, the revision improves the manuscript, but it does not resolve the central issue. I still do not think the manuscript reaches the threshold for publication in Nature Communications.

Open Access This Peer Review File is licensed under a Creative Commons Attribution 4.0 International License, which permits use, sharing, adaptation, distribution and reproduction in any medium or format, as long as you give appropriate credit to the original author(s) and the source, provide a link to the Creative Commons license, and indicate if changes were made.
In cases where reviewers are anonymous, credit should be given to 'Anonymous Referee' and the source.
The images or other third party material in this Peer Review File are included in the article's Creative Commons license, unless indicated otherwise in a credit line to the material. If material is not included in the article's Creative Commons license and your intended use is not permitted by statutory regulation or exceeds the permitted use, you will need to obtain permission directly from the copyright holder.
To view a copy of this license, visit https://creativecommons.org/licenses/by/4.0/

REVIEWER COMMENTS

Reviewer #1 (Remarks to the Author):

This manuscript presents an improved chromosome-scale genome assembly (Lo7_V3) for the inbred rye line Lo7, leveraging PacBio HiFi, ONT, Hi-C, and BioNano technologies. The authors highlight advancements in contiguity, centromere assembly, and annotation over previous versions (Lo7_V1/V2), alongside analyses of centromeric retrotransposon dynamics and 1RS translocation diversity in wheat-rye hybrids. While the resource is valuable for the community, the study lacks novelty relative to recent rye genome publications (e.g., Rabanus-Wallace et al. and Li et al., Nat Genet 2021) and suffers from critical methodological omissions. Key claims—including centromere "shifts" and species-specific TE activity—are inadequately supported, and technical flaws (e.g., low sequencing depth, incomplete benchmarking) undermine confidence in the assembly. Overall, the work falls short of the standards expected for publication in Nature Communications and requires major revisions to address the following concerns.

Major revisions:
1. Insufficient assembly quality assessment, the reliance on LAI (LTR Assembly Index) alone is inadequate for evaluating assembly completeness and accuracy. Standard metrics like BUSCO scores (for gene space), Merqury QV (consensus accuracy), and coverage uniformity of HiFi/ONT reads mapped to the assembly must be included. These omissions obscure potential biases in repetitive regions and undermine claims of "high quality."

Response: We thank the reviewer for this suggestion. The BUSCO scores are provided in Table 1 and Supplementary Table 5. We have provided now in addition to LAI the Merqury QV value of 64.66 (see L119 from revised main text).
We also provided genome-wide distribution of HiFi and ONT coverage alongside GC content (Supplementary Fig. 5).
For HiFi coverage uniformity,

chr1R	mean=37.90	sd=5.52 CV=0.146
chr2R	mean=38.72	sd=5.89 CV=0.152
chr3R	mean=38.20	sd=6.70 CV=0.175
chr4R	mean=37.69	sd=5.59 CV=0.148
chr5R	mean=37.35	sd=5.87 CV=0.157
chr6R	mean=37.56	sd=5.75 CV=0.153
chr7R	mean=37.91	sd=5.25 CV=0.139

For ONT coverage uniformity,

chr1R	mean=9.28	sd=2.45 CV=0.264
chr2R	mean=9.64	sd=3.13 CV=0.325
chr3R	mean=9.83	sd=3.26 CV=0.331
chr4R	mean=9.24	sd=2.45 CV=0.265
chr5R	mean=9.23	sd=2.55 CV=0.276
chr6R	mean=9.23	sd=2.48 CV=0.268
chr7R	mean=9.30	sd=2.38 CV=0.256

2. The sequencing depth, particularly for Hi-C data (20X), is insufficient for a genome as complex as rye—characterized by outcrossing behavior, high heterozygosity, large size, and extensive repeats. Hi-C data at this depth may fail to resolve chromosomal interactions in repetitive regions, leading to misassemblies or incomplete scaffolding. The authors must justify why 20X Hi-C is adequate for their purposes or provide additional Hi-C data to support the chromosome-scale assembly.

Response: We appreciate the reviewer's concern regarding the Hi-C sequencing depth. The Lo7 genotype used in this study is a highly inbred line developed by KWS and contains only residual heterozygosity. Based on our experience with the sequencing and assembly of several hundred diploid and polyploid Triticeae genomes, we have found that a Hi-C coverage of approximately 20× is sufficient to achieve high-quality chromosome-scale assemblies in such material.

In the case of Lo7_V3, scaffolding resulted in 5–24 contigs per chromosome (see Supplementary Fig. 4), and the Hi-C contact maps did not reveal any misassemblies or inconsistencies (see Supplementary Fig. 2). Moreover, the scaffolding was independently validated with a Bionano optical map, which confirmed the structural integrity of the assembly (see Supplementary Fig. 3). As an example, the scaffolding of chromosome 1R was verified by both Hi-C (**Response Figure 1**) and optical map data, and the same strategy was applied across all chromosomes.

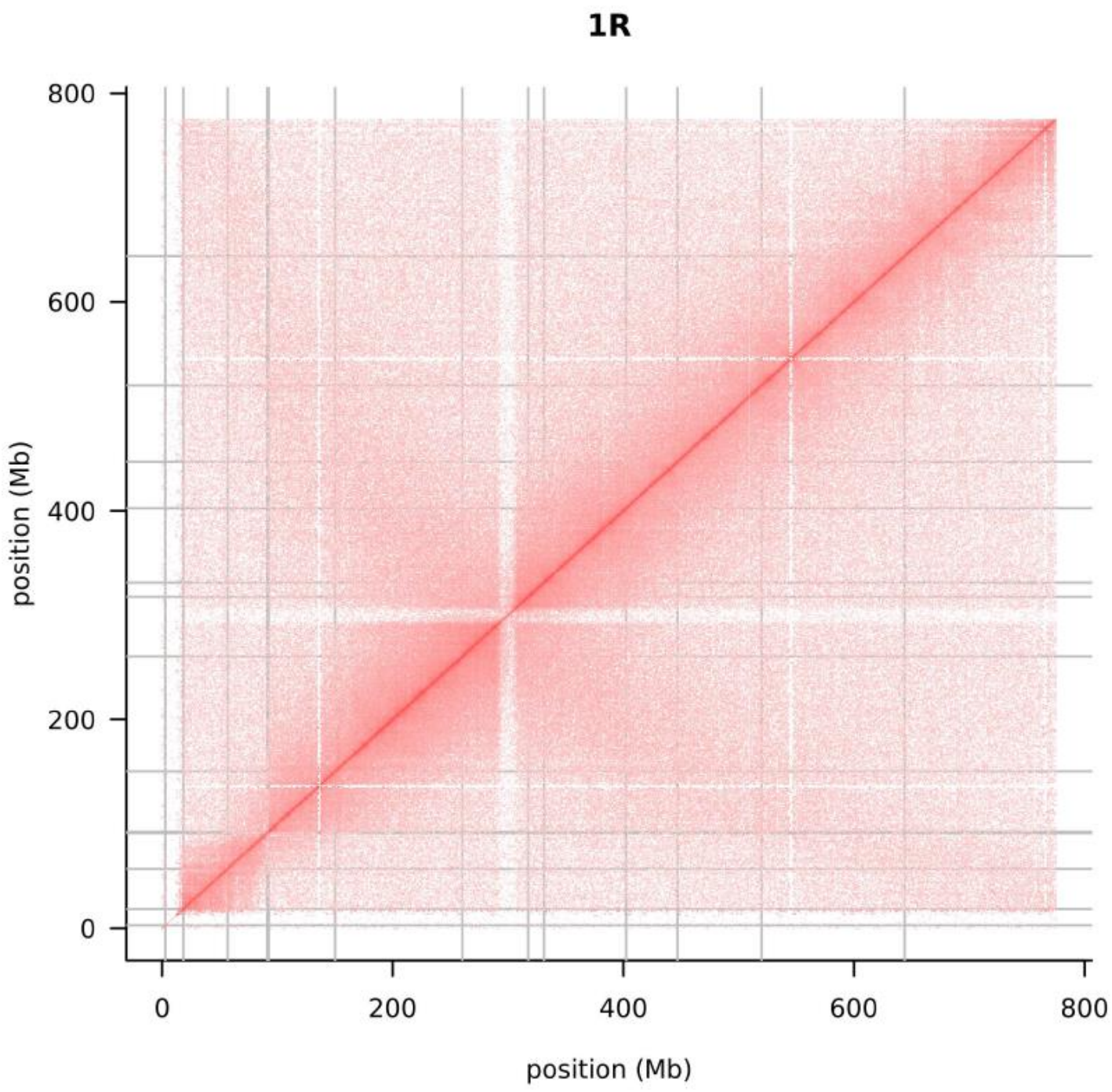

Response Figure 1. Hi-C map of Lo7_V3 1R. Grey lines indicate contig gaps.

3. The updated gene annotation (75,850 genes) lacks mapping to previous Lo7_V2 genes. Without clear correspondence (e.g., shared identifiers or synteny analysis), transitioning between versions is impractical for end users. A gene liftover track or comparative table is essential.

Response: We appreciate the reviewer's emphasis on ensuring that genome resources remain traceable and useful for the community, which includes the need of tracing gene models in different assembly versions. The gene liftover table was now provided as Supplementary Table 12.

4. The reported genome size of Lo7_V3 (6.76 Gb) is substantially smaller than the ~7.9 Gb estimated for rye in two 2021 Nature Genetics studies, including a flow cytometry-based measurement for Weining rye. This discrepancy raises concerns about potential assembly gaps or underrepresentation of repetitive regions. The authors must investigate and explicitly explain this difference—whether it stems from assembly errors, insufficient sequencing depth, biological variation between accessions, or other factors—and provide evidence to support their explanation.

Response: We appreciate the reviewer's comment on the genome size discrepancy. The smaller assembly size of Lo7_V3 (6.76 Gb) compared to previous reports (e.g., ~7.9 Gb for Weining rye) can be explained by several factors. First, Lo7 is a highly inbred genotype, whereas Weining exhibits a low level of inbreeding, which likely results in partial haplotype merging in the Weining assembly and therefore larger estimated genome sizes. Second, Lo7 possesses extensive cytologically visible STR-based heterochromatic regions that represent the main source of remaining gaps in Lo7_V3, potentially contributing to small underestimations in total size.

Regarding the flow-cytometric estimates, as discussed in the barley MorexV3 paper[1], such measurements depend on the assumed size of the human genome standard. The conventional value of 3.5 pg corresponds to 3.423 Gb per haploid genome[2], whereas the current human reference assembly (GRCh38.p14) spans approximately 3.1 Gb. Adjusting the standard accordingly reduces the estimated size of the rye genome to around 7.15 Gb, which aligns more closely with Lo7_V3.

In addition, recent HiFi-based plant genome assemblies consistently report slightly smaller total assembly sizes compared with flow-cytometry estimates, reflecting the combination of collapsed repetitive regions and the exclusion of unassembled highly repetitive DNA. Together, these factors explain the apparent difference between Lo7_V3 and earlier estimates based on flow cytometry.

5. The claim of multiple centromere shifts on chromosome 7R, inferred from TE insertion patterns, is inadequately validated. The authors rely solely on TE age and distribution, without confirming these shifts using Hi-C interaction data (e.g., changes in contact intensity at proposed inversion breakpoints) or synteny analyses with other rye accessions. Without such validation, these shifts could be artifacts of assembly errors. The authors must strengthen this claim with orthogonal evidence, including structural variant calls from Hi-C and comparative analyses with related genomes.

Response:
As demonstrated by Ahmed *et al.*[3] in *Triticum monococcum*, the distribution and estimated ages of centromere-specific TE insertions can be used to infer centromeric inversions (see

their Supplementary Fig. 6). Following this approach, we applied the same analysis to the *S. cereale* Lo7 assembly. The results support our evolutionary model: the youngest predicted inversion (Inversion C) in Lo7 is absent in Lo225, another high-quality rye genome assembly that will be described elsewhere.

The Hi-C interaction plot for Lo7 chromosome 7R shows no indication of mis-assembly in the centromeric region (**Response Figure 2**). A dot-plot alignment of the 7R centromere between Lo7 and Lo225 (**Response Figure 3**) confirms the structural difference: the cluster of young TE insertions near ~455 Mb in Lo7 is absent at the corresponding position (~460 Mb) in Lo225, consistent with an inversion detected by sequence comparison (**Response Figure 3b**). A comparable pattern was also observed for chromosome 2R, where Lo225 carries a recent inversion (**Response Figure 4**). The corresponding dot-plot alignment between Lo7_V3 and Lo225_V1 has been deposited at https://doi.org/10.5281/zenodo.17434221.

The assembly accuracy in the investigated region of Lo7 chromosome 7R is further supported by optical-map validation and Hi-C contact patterns, both of which show no evidence of mis-assembly at the proposed breakpoints. In response to the reviewer's suggestion, we now cite the *T. monococcum* study in the Results section (L229) and have rephrased the corresponding text more cautiously, as the Lo225 data are not yet published.

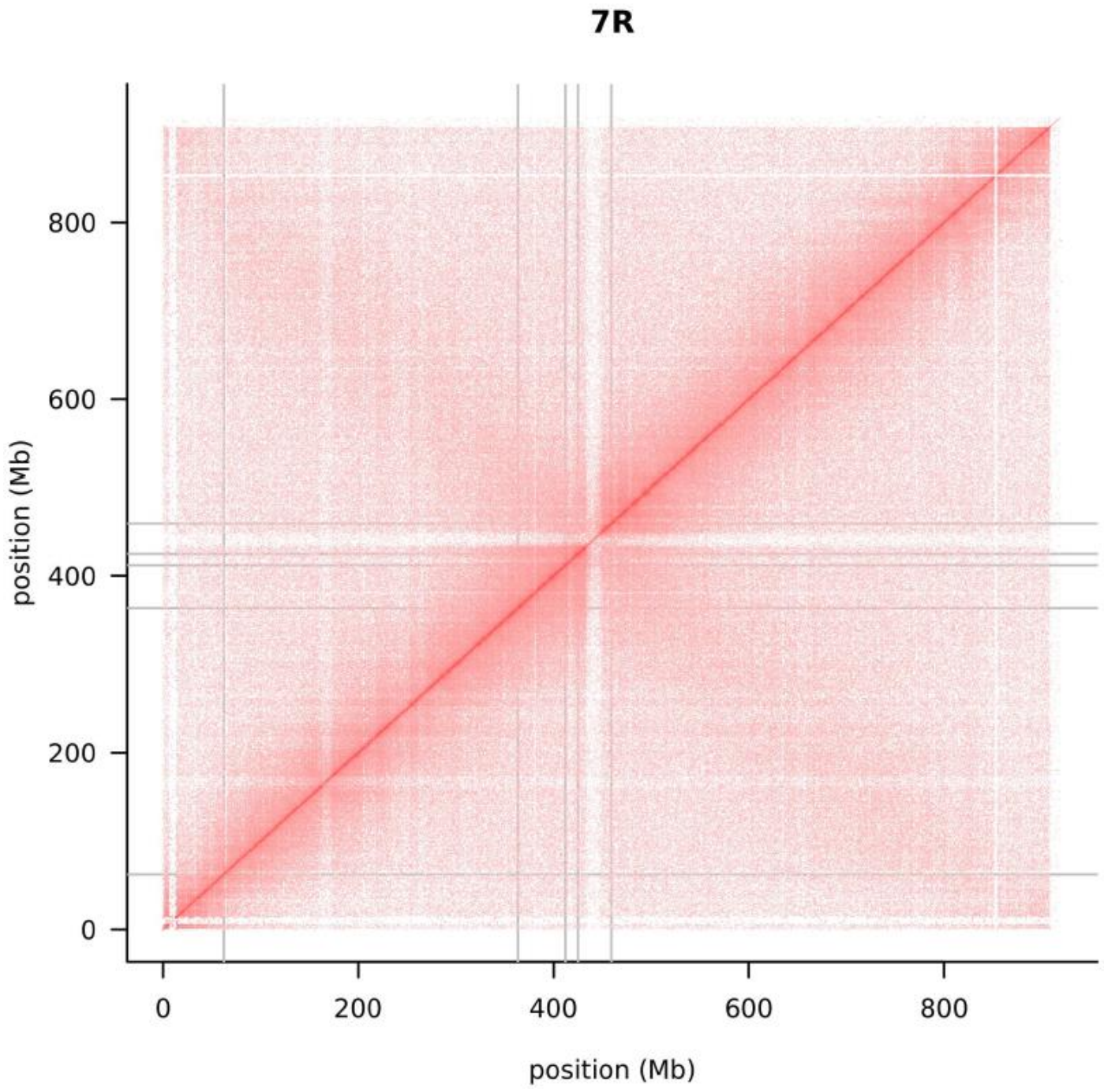

Response Figure 2. Hi-C map of Lo7_V3 7R. Gray lines indicate contig gaps.

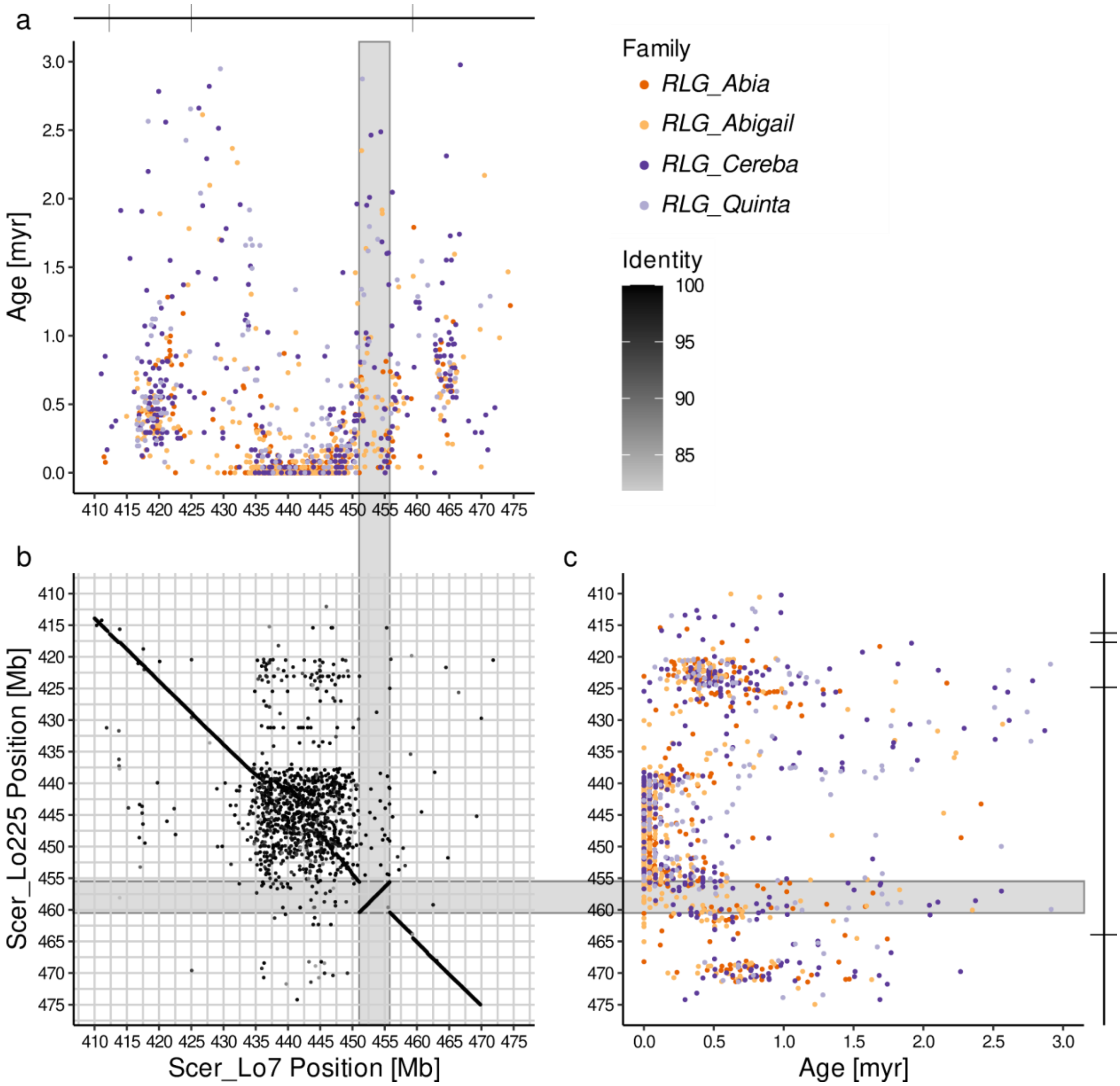

Response Figure 3. Comparison of chromosome 7R in Lo7 and Lo225 assemblies. a and c show the distribution of the four centromere-specific retrotransposon families *RLG_Abia*, *RLG_Abigail*, *RLG_Cereba* and *RLG_Quinta* on chromosome 7R of *S. cereale* Lo7 and *S. cereale* Lo225 respectively. Gaps in the assemblies are indicated by the black strokes on the top and right side of the panels respectively. b A blast comparison between the centromeric regions in Lo7 and Lo225. The segment size was 1000 bp with a sliding step of 10 kb. Identity is indicated by the shades of gray. The gray boxes highlight the area of inversion in both genomes.

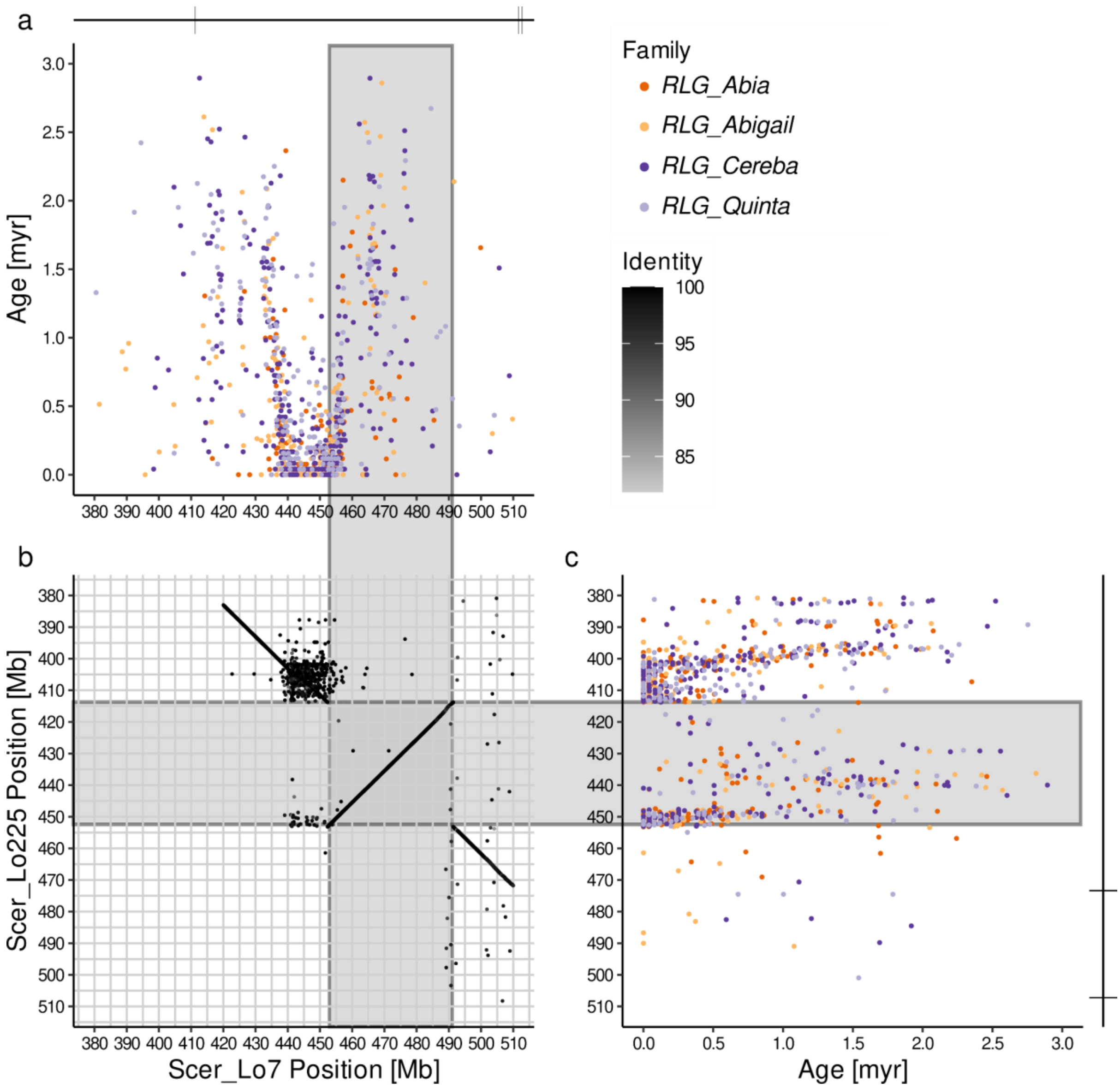

Response Figure 4. Comparison of chromosome 2R in Lo7 and Lo225 assemblies. a and c show the distribution of the four centromere-specific retrotransposon families *RLG_Abia*, *RLG_Abigail*, *RLG_Cereba* and *RLG_Quinta* on chromosome 2R of *S. cereale* Lo7 and *S. cereale* Lo225 respectively. Gaps in the assemblies are indicated by the black strokes on the top and right side of the panels respectively. b A blast comparison between the centromeric regions in Lo7 and Lo225. The segment size was 1000 bp with a sliding step of 10 kb. Identity is indicated by the shades of gray. The gray boxes highlight the area of inversion in both genomes.

6. The conclusion that RLG_Abia is "highly active" in rye but suppressed in wheat overlooks technical confounders. The superior continuity of Lo7_V3 likely enables more complete TE annotation, whereas fragmented wheat assemblies may miss elements. To assert biological divergence, the authors must reannotate a high-quality wheat genome (e.g., CS_T2T) using their pipeline and statistically compare TE abundances.

Response:

In response to the reviewer's comment, we ran EDTA specifically on the identified functional centromeres of *S. cereale* Lo7, the suggested *T. aestivum* CS-IAAS v1.1 genome and *T. monococcum* TA299 (a high-quality assembly with completely assembled centromeres)[1] with a newly compiled library of TE sequences from wheat and rye. We could show that *RLG_Abia* contributes ~40-50% to centromeres of Lo7 while it on average only accounted for ~2.64% of the centromeres in *T. monococcum* TA299 and ~4.21% in hexaploid wheat (Supplementary Fig. 15). Because we used an improved TE library which included additional wheat sequences, we identified a non-autonomous version of *RLG_Abia* (which we named *RLG_Abigail*) and which was abundant in rye centromeres. This improved overall annotation of the centromeric regions in *S. cereale* Lo7 where we can now annotate ~95% of centromeres with the 4 TE families *RLG_Abia*, *RLG_Abigail*, *RLG_Cereba* and *RLG_Quinta*. We now included *RLG_Abigail* in all our centromere analyses and revised the results section accordingly. We found *RLG_Abigail* to be highly abundant in Lo7 in comparison to *T. monococcum* and *T. aestivum*, and *RLG_Cereba* as well as *RLG_Quinta* were both more abundant in *T. monococcum* and *T. aestivum* in comparison to Lo7 (Supplementary Fig. 15).

Additionally, in response to Reviewer 3 (see below) we analyzed expression data bioprojects PRJEB35461 for rye (*S. cereale* Lo7) and PRJNA427159 for wheat (*T. aestivum* vc. Chinese Spring). We discovered that all 4 centromeric retrotransposon families (*RLG_Abia*, *RLG_Abigail*, *RLG_Cereba* and *RLG Quinta*) are found among the top 10 most expressed LTR retrotransposons in rye (from a total of 61 LTR retrotransposon families). In contrast, only *RLG_Cereba* and *RLG Quinta* were among the top 10 in wheat, while transcripts of *RLG_Abia* and *RLG_Abigail* were barely detectable in wheat (Supplementary Fig. 20). Additionally, rye centromeres contain high numbers of very young copies of *RLG_Abia* and *RLG_Abigail*, while wheat and *T. monococcum* only contain few and exclusively old copies. We include this information now in the main text (L248) and provide additional supplementary figures 15 and 20.

7. Additionally, The authors do not quantify the accuracy of repeat annotations (e.g., consistency of TE family classification with Lo7_V2) or explain the root causes of errors in Lo7_V2 (e.g., 1R inversions). Without evaluating potential biases in assembly tools (e.g., short-read assemblers), it is unclear whether improvements stem from technological advances or parameter optimization.

Response: We thank the reviewer for this comment. We have provided an updated and revised Supplementary Table 6, adressing this point. We provided the precised annotation of repeats and summary of genome-wide prominent LTR annotations in Lo7_V2 and Lo7_V3.

Reviewer #2 (Remarks to the Author):

Rye is an important cereal crop within the tribe Triticeae, which poses unique challenges for genome assembly due to its large genome, high repeat contents, and extensive structural heterogeneity. In this study, the authors present a chromosome-scale, near-complete genome assembly for rye inbred variety Lo7, utilizing PacBio HiFi, Oxford Nanopore, Hi-C, and BioNano sequencing and TRITEX pipeline. Compared with previous versions, the

assembly further improved the resolution of complex repetitive regions in subtelomeres and centromeres. This work makes rye more accessible to genomic research and a valuable resource that will support future genomic research and crop improvement efforts in rye and related cereal species.
Overall, the logic is good, the analyses are clear and data are reliable. Here are some comments:

1. Some errors/typos in the manuscript, either from the text or from the figures, need to be carefully checked and revised. Here are only several examples.
(1) Please ensure consistent decimal precision for numerical values throughout the manuscript, such as ~0.6 million years ago (Line 219), 416 Mb (Line 220), 0.25 mya (Line 221) and so on. Please carefully review the entire text.

Response: Thank you for pointing this out. We have carefully revised the manuscript and believe that we have removed all such inconsistencies. Any remaining issues will be identified during the copy-editing stage.

(2) Some specialized terms should be italicized, such as the names of species (e.g., in reference 23) and genes (e.g., in reference 4). Authors are kindly requested to carefully review the entire text.

Response: Thanks. We carefully revised the manuscript.

2. The chloroplast sequences were removed from the final rye genome. Were the mitochondrial genome sequences removed from the final rye genome as well? Please provide an explanation in the Methods section.

Response: We have removed chloroplast and mitochondrial genome sequences from the final genome assembly.

3. Compared to previously published rye genomes, how many additional genes were identified in the new assembly, and how is the gene prediction accuracy? What are the methodological criteria for distinguishing high-confidence versus low-confidence genes in the annotation pipeline? Please provide an explanation in the Methods section.

Response: We provided the methods for distinguishing high-confidence versus low-confidence genes in the annotation pipeline. We classified predicted proteins into high and low confidence categories. High-confidence proteins had over 80% coverage and were complete, either with significant hits in UniMag or UniPoa but not PTREP. Low-confidence proteins were incomplete with hits in UniMag or UniPoa but not PTREP, or were complete without hits in any database. We provided additional genes identified in each chromosome between Lo7_V2 and Lo7_V3, which were validated using BUSCO (see Supplementary Table 5).
As shown in Supplementary Table 5, we quantify the additional HC genes identified in the new assembly,

HC genes_annotation	Lo7_V2	Lo7_V3
HC_chr1R	4045	5113

HC_chr2R	5049	6366
HC_chr3R	4410	5934
HC_chr4R	4837	7045
HC_chr5R	5155	6645
HC_chr6R	4457	6573
HC_chr7R	4549	5964
HC_chrUn	1939	114

4. Please provide the accession numbers (e.g., NCBI/ENA/DDBJ) of all released datasets associated with this study in the data availability section.

Response: We have provided all accession numbers of the datasets newly generated for this study. Please check the section, Data Availability.

5. T2T genome assemblies of crops have significantly expanded our understanding of genome architecture and function. In the Discussion section, please discuss the potential strategies to advance this rye genome assembly to T2T level in the future.

Response: As discussed in the second paragraph (L299-305) of the Discussion, we identified the main limitations and future directions for rye T2T genome assembly. Which raises the key question: how can we generate longer contigs and scaffolds in rye genome assembly? Besides sufficient HiFi long reads, it is likely that incorporating more than 100× ultra-long Oxford Nanopore (ONT) sequencing reads could substantially improve assembly continuity and completeness. Only with such ultra-long reads it may be possible to span the highly repetitive satellite sequence regions and ultimately close most of the remaining gaps, faciliating the rye genome assembly closer to a real telomere-to-telomere (T2T) level, however, this remains speculative, and achieving a true T2T assembly may ultimately depend on future technological advances that will make it possible to bridge multimegabase repeat regions that are lacking sequence polymorphisms in their subunits.

Reviewer #3 (Remarks to the Author):

This manuscript presents an updated assembly of Rye Lo7 genome. The authors have used that latest PacBio HiFi, ONT and Hi-C to create a much more contiguous assembly than previously available, which fully resolves the centromeric sequences and provides an useful updated resource for the field. It highlights the role of Gypsy superfamily transposons which form the majority of the centromere sequences and use the updated assembly to investigate the sequence diversity of wheat/rye 1RS. Overall, I consider the manuscript of excellent quality and well written.

I only have a couple of comments:

1. The manuscript could benefit from a more extensive discussion on the evolutionary implications of centromeric retrotransposon dynamics in other cereal genomes. In the centromere TEs found RLG_Cereba, RLG_Quinta and RLG_Abia, a comparative analysis how

similar are these TEs in structure to the RLG_Cereba and RLG_Quinta found in closely related species such as wheat or barley, would enhance evolutionary context.

Response: In response to the reviewer's comment, we have performed extensive additional analysis comparing homologs of the 4 centromeric retrotransposon families from wheat (*T. aestivum*), Einkorn wheat (*T. monococcum*) and barley (*H. vulgare*). All four retrotransposon families can be found in other members of the *Triticeae*. We now include a new supplementary Figure (Supplementary Fig. 15) which shows DNA sequence conservation of the individual families across the *Triticeae*. In addition, we found that *RLG_Abia* and *RLG_Abigail* are generally much older in wheat in comparison to rye, indicating that they were inactive for a long time in wheat, while these two TE families have recently been active in rye, hence the high number of young insertions (Supplementary Fig. 17 and 20). See also our response to the comment of reviewer 1 above.

An interesting point to mention here is that a segment of *RLG_Abia* had been used in previous studies as a centromere specific probe in FISH assays (pAWRC.1)[2,3]. This segment was previously referred to as "*Bilby*" but should actually be re-classified as *RLG_Abia* according to the reference wheat TE annotation[4]. We now also added this information and corresponding references in the text.

2. Additionally, is there any specific mutations or domain structures in these elements that are specific to the centromere clades that could explain the involvement in centromere function? Such as those recently reported (Tsukahara, S., Bousios, A., Perez-Roman, E. et al. Centrophilic retrotransposon integration via CENH3 chromatin in Arabidopsis. Nature 637, 744–748 (2025). https://doi.org/10.1038/s41586-024-08319-7)

Response: There are several studies that propose that a unique protein domain (CR domain) that is fused to the integrase of centromere-specific retrotransposons is responsible for their centromere specificity[5,6]. The evidence shows that the CR domain specifically recognized CENH3 histone variants thereby guiding new insertions into the functional centromere[7]. We now added the information on these studies to the introduction. We also emphasize that they are distinct from the *Copia* superfamily element *Tal1* (*Transposon of Arabidopsis lyrata 1*)[8] mentioned by the reviewer, as they belong to the *Gypsy* superfamily and the mechanism by which *Tal1* targets CENH3 chromatin in *Arabidopsis* is unknown.

3. Activity and Dynamics of RLG_Abia (Lines 197-198): Is there any evidence in transcriptome data that the RLG_Abia retrotransposons are actively transcribed? Is this continuing ongoing integration at the centromere or a burst of centromeric integration creating a younger region in which the functional centromere shifts?

Response: In response to the reviewer's comment, we have analyzed transcriptome data from rye and wheat (See also our response to the comment of reviewer 1 above). We found that all 4 centromeric retrotransposon families (*RLG_Abia*, *RLG_Abigail*, *RLG_Cereba* and *RLG Quinta*) are found among the top 10 most expressed LTR retrotransposons in rye (from a total of 61 LTR retrotransposon families). In contrast, only *RLG_Cereba* and *RLG Quinta* were among the top 10 in wheat, while transcripts of *RLG_Abia* and *RLG_Abigail* were barely

detectable. We describe this now in the main text (L249) and provide the data in the new Supplementary Figure 20.

Regarding the second question, we understand the reviewer to be asking whether targeted insertion of centromere-specific retrotransposons may have driven the observed centromere shifts. As mentioned in a response to another comment of the reviewer (see above), the centromeric retrotransposons encode a so-called CR-domain which is fused to their integrase, which has long been discussed to play a role in their integration specificity[5,6]. Recently, it was proposed that the CENH3 histone variants are recognized by the CR domain of the TEs thus guiding new insertions into the functional centromere. We added this information in the introduction.

4. In the methylation profiling only CG context reported, however all contexts could be called using the Nanopore data which would enhance the manuscript – Particularly it would be interesting to see this profile over the centromere regions which often have a centromeric dip region in CG or CHG context methylation

Response: We thank the reviewer for this insightful suggestion. We explored the possibility of detecting CHG and CHH methylation from our ONT data. However, several technical barriers prevent us from generating results with sufficient reliability:
1) Tool compatibility: Unlike PacBio 5mC, ONT does not provide an official pipeline for methylation calling, with DeepSignal[9] being the only prevalent tool at the moment for this purpose. However, the plant version of the tool DeepSignal-plant (https://github.com/PengNi/deepsignal-plant), is so far not compatible with the Dorado basecaller, which is required to handle our raw-signal data in the newest pod5 format;
2) Lack of a trained model for rye: Accurate detection of non-CG methylation from ONT data requires species-specific trained models, which are not yet available for rye; existing models are only available for rice and Arabidopsis (DeepSignal3 and DeepSignal-plant);
3) Data coverage: Our ONT dataset (9x) was generated primarily for genome assembly support. This coverage is insufficient for robust methylation analysis (and model training) across all contexts, particularly in repetitive centromeric regions.
Since this work presents the first fully assembled and validated rye centromeres, we wish to avoid reporting preliminary epigenomic patterns that may not be reliable. Instead, we have noted this opportunity for future investigation, and all ONT data are publicly available at European Nucleotide Archive (ENA) under project ID PRJEB91463, so the community can revisit this analysis when appropriate tools become available.

Minor:
5. In Fig 2 a comparative plot between the old and new assembly would be beneficial to compare the updated sequence - like the syri plot in Supplementary Figure 13

Response: Thanks. Please see Supplementary Fig. 7.

References:
1 Ahmed, H. I. *et al.* Einkorn genomics sheds light on history of the oldest domesticated wheat. *Nature*, doi:10.1038/s41586-023-06389-7 (2023).

2 Francki, M. G. Identification of *Bilby*, a diverged centromeric Ty1-*copia* retrotransposon family from cereal rye (*Secale cereale* L.). *Genome* 44, 266-274, doi:10.1139/g00-112 (2001).
3 Francki, M. G., Berzonsky, W. A., Ohm, H. W. & Anderson, J. M. Physical location of a *HSP70* gene homologue on the centromere of chromosome 1B of wheat (*Triticum aestivum* L.). *Theor Appl Genet* 104, 184-191, doi:10.1007/s00122-001-0781-4 (2002).
4 Wicker, T. *et al.* Impact of transposable elements on genome structure and evolution in bread wheat. *Genome Biol* 19, 103, doi:10.1186/s13059-018-1479-0 (2018).
5 Gao, X., Hou, Y., Ebina, H., Levin, H. L. & Voytas, D. F. Chromodomains direct integration of retrotransposons to heterochromatin. *Genome Res* 18, 359-369, doi:10.1101/gr.7146408 (2008).
6 Neumann, P., Novak, P., Hostakova, N. & Macas, J. Systematic survey of plant LTR-retrotransposons elucidates phylogenetic relationships of their polyprotein domains and provides a reference for element classification. *Mob DNA* 10, 1, doi:10.1186/s13100-018-0144-1 (2019).
7 Heuberger, M. *et al.* Evolution of Einkorn wheat centromeres is driven by the mutualistic interplay of two LTR retrotransposons. *Mob DNA* 15, 16, doi:10.1186/s13100-024-00326-9 (2024).
8 Tsukahara, S. *et al.* Centrophilic retrotransposon integration via CENH3 chromatin in Arabidopsis. *Nature* 637, 744-748, doi:10.1038/s41586-024-08319-7 (2025).
9 Ni, P. *et al.* DeepSignal: detecting DNA methylation state from Nanopore sequencing reads using deep-learning. *Bioinformatics* 35, 4586-4595, doi:10.1093/bioinformatics/btz276 (2019).

REVIEWER COMMENTS

Reviewer #1 (Remarks to the Author):

Although the authors have addressed several technical concerns raised in the first round and improved the assembly metrics with additional quality control (Merqury QV, BUSCO, coverage distribution), the revised manuscript remains primarily incremental. The study reports an upgraded genome assembly (Lo7_V3) and descriptive analyses of centromeric retrotransposons but fails to deliver substantive biological novelty or mechanistic insight. The revisions mainly add data rather than resolve the conceptual issues previously noted.

Major reasons for rejection:

1. Limited conceptual advance and biological novelty. The new version continues to focus on technical improvement—longer contigs, fewer gaps, and slightly enhanced annotation accuracy—without introducing a new biological discovery. The main results, such as the completion of seven centromeres and the high abundance of RLG_Abia/RLG_Abigail elements, are descriptive in nature. There is no experimental or computational evidence explaining how these transposons influence centromere function, structure, or evolution. The work therefore reads as a genome-resource update rather than a research paper with conceptual impact suitable for Nature Communications.

Response: We thank the reviewer for his/her personal view. While we agree that the manuscript reports a highly valuable community resource, however, it is not an update but a de novo sequence assembly, derived by different technology. Previous inherent limitations of the short-read based genome assembly of Lo7 were removed as independently documented by a newly developed optical map. We provide detailed analysis on the full set of seven rye centromeres and demonstrate that structural variation in (proximity to) the centromere is a frequent feature in rye. Considering data from a recently published rye genome, allow us to provide additional independent proof of our hypothesis, providing robust support for the dating of independent events.

2. Insufficient validation of the proposed centromere shifts on chromosome 7R. The authors added additional analyses to support their model of three consecutive inversions on 7R, including dotplot comparisons with Lo225, Hi-C contact maps, and optical map consistency checks. However, these additions remain insufficient to substantiate the claim of multiple centromere shifts. The interpretation is still derived mainly from the distribution and estimated ages of centromeric retrotransposons, which represent indirect correlations rather than structural evidence. No independent breakpoint-level validation, statistical support, or reproducible synteny analysis across multiple published rye genomes is provided. Without explicit sequence-based inversion calls and public access to the Lo225 data, this claim remains speculative and does not meet the evidentiary standard for a high-impact journal.

Response: In order to respond to the reviewer's comment, we took advantage of recently published data for another rye cultivar Weining. After initial assessment we observed structural flaws of the published assembly version (Weining.V2)[1] (**Supplementary Fig. 12-13**). Therefore, we downloaded all public data and reassembled the genome de novo (Weining.V3). Weining contains much higher heterozygosity than Lo7 (**Supplemental Fig. 14**), thus we obtained a phased assembly of two haplotypes (H1 and H2) of Weining, allowing for several comparative analyses (see also response to comment 4 below). We demonstrate that Weining.V3 H1 is more distantly related to Lo7 and does

not contain the most recent inversion proposed in our model (Inversion C) (**Fig. 5d**). This allowed us to precisely identify the breakpoints of this inversion. Breakpoints in Lo7 are now provided in the main text (Lo7 chr7R:451071203-455788938) and the corresponding breakpoints in Weining.V3 H1 (chr7R:457005525-462401440) in the **Supplementary Fig. 29**. This demonstrates that centromeric inversions can indeed be inferred from positions and estimated insertion ages of retrotransposons, in cases where their local pattern (hereafter called “spatio-temporal clusters”) deviates from the typical “volcano” shape that is expected in centromeres that do not have inversions. The results of the comparison are now shown in **Supplementary Fig. 29**. Thus, this generally supports our hypothesis that the particular spatio-temporal TE clusters present in Lo7 chromosome 7R are the result of inversions. Drawing on insight from the analysis of Weining.V3 and the comparison to Lo7, we were able to improve our evolutionary model for Lo7 (**see Fig. 5**). For the two older proposed inversions, we narrowed down the putative break points. However, since these are older, we do not have genome assemblies of haplotypes that do not carry the inversions and thus cannot provide exact breakpoints. Nevertheless, we now also use more cautious language and emphasize the presence of inversions in the (peri-) centromeric regions of 7R.

This analysis was possible only by generating a reassembly of the recently published Weining data[1]. For your transparent review, all methods and results required to support our conclusions were included in the main manuscript (**Line 181**) and supplementary data files (**Supplementary Fig. 12-18**).

(1) Collapsed genome assembly of Weining.V1 and Weining.V2

To benchmark our Lo7v3 assembly we conducted a collinearity analysis to illustrate the synteny among the assemblies Weining.V1[2] and the recently published Weining.V2[1]. Compared with the highly homozygous Lo7_V3 reference, the Weining assemblies exhibited several redundant haplotypic segments (see red circles in the response figures 1+2), suggesting the presence of duplicated haplotype representations in the assembly. These regions likely reflect residual heterozygosity or incomplete haplotype collapsing during the assembly process.

a)

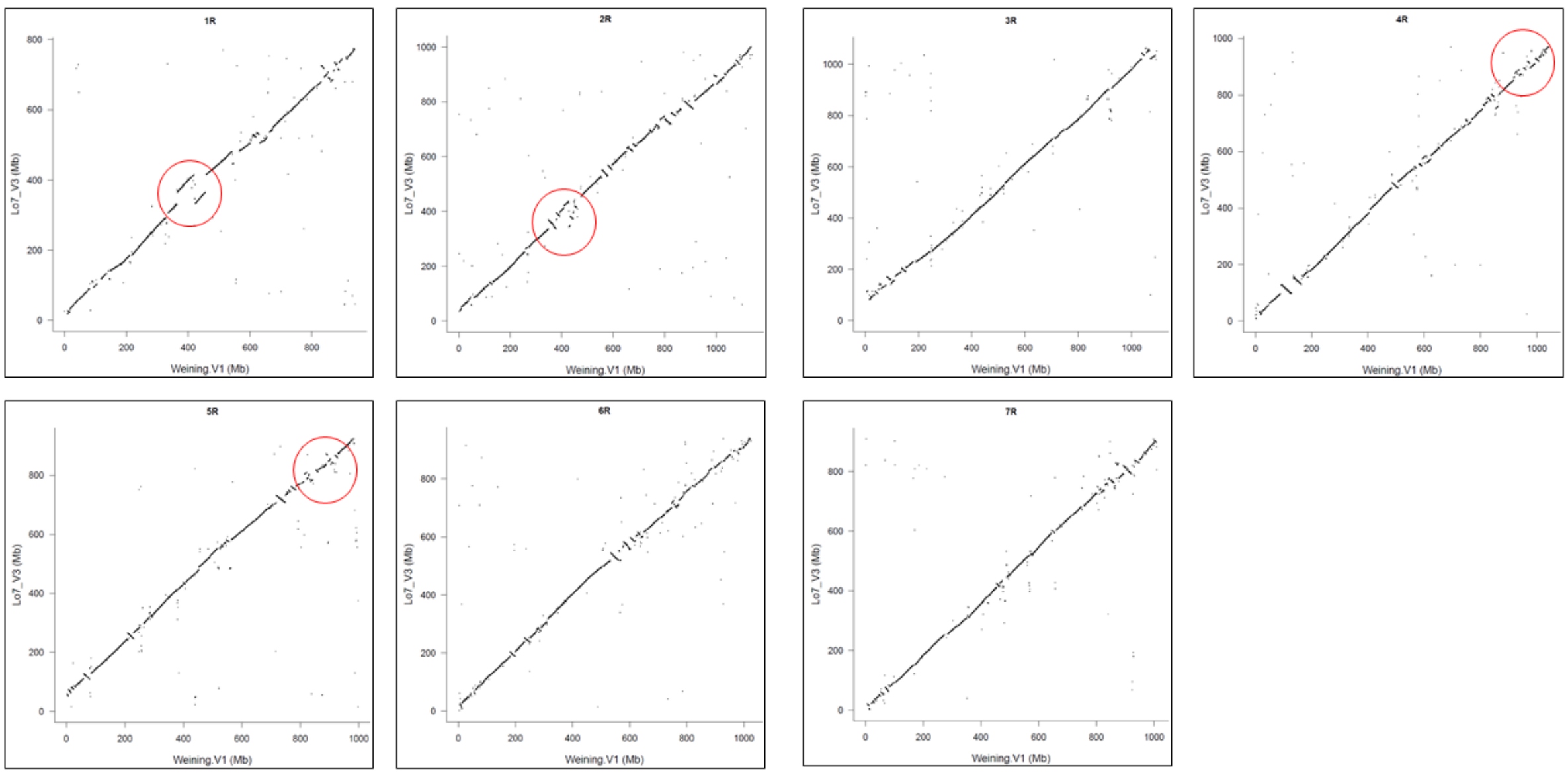

b)

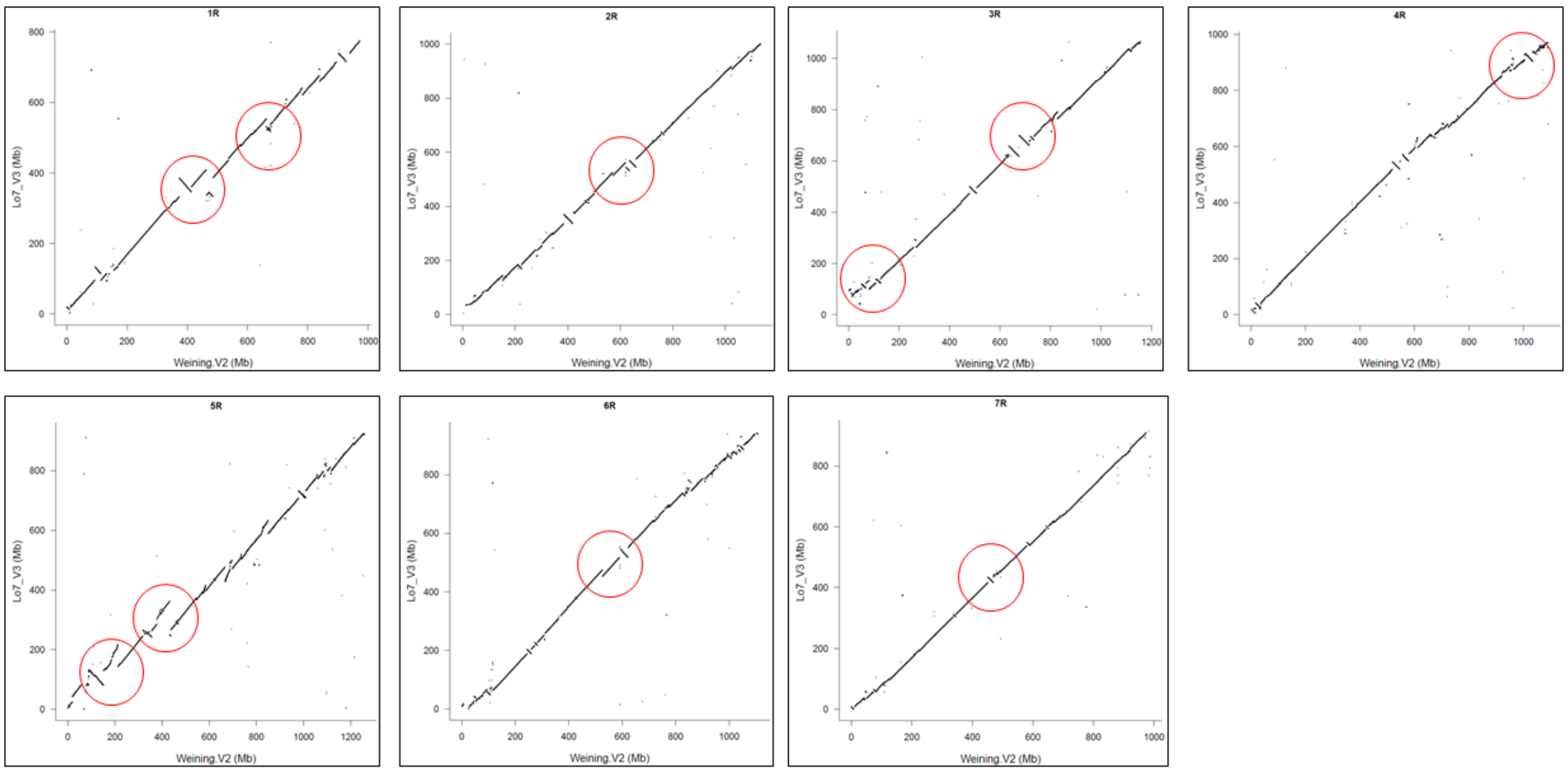

Response Figure 1: From our dotplot comparisons with Lo7_V3 we found that (a) Weining.V1[2] and (b) Weining.V2[1] represent partially redundant genome assemblies exhibiting incomplete collapsing of haplotypes, thus causing genome size inflation and artificial redundancy (indicated by red circles). Therefore, to perform a meaningful comparison, we decided to use all public data of Weining.V2[1] for reassembly, in order to have comparable assembly quality and a maximal reduction of assembly quality differences that are attributed to different assembly strategies.

We performed Hi-C mapping of the published Weining Hi-C data using HapHiC[3] on the Weining.V2 genome assembly[1] and found that haplotype redundancy exists on certain chromosomes (**Response Figure 2a +b**).

Heterozygosity, estimated from HiFi long reads, was found to be 0.54% (**Supplementary Fig. 14**). Therefore, we considered a phased genome assembly of Weining necessary for haplotype analysis.

a)

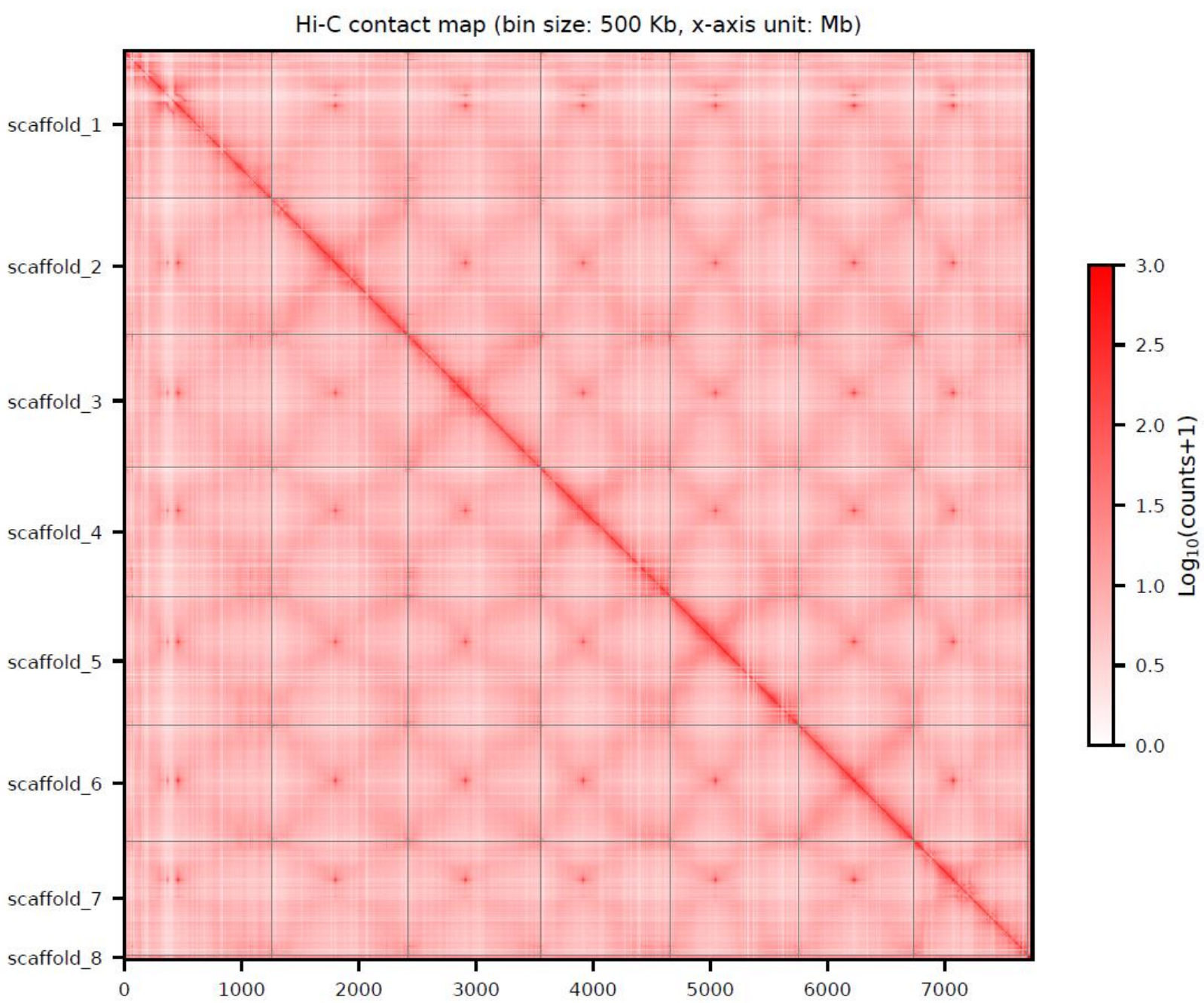

b)

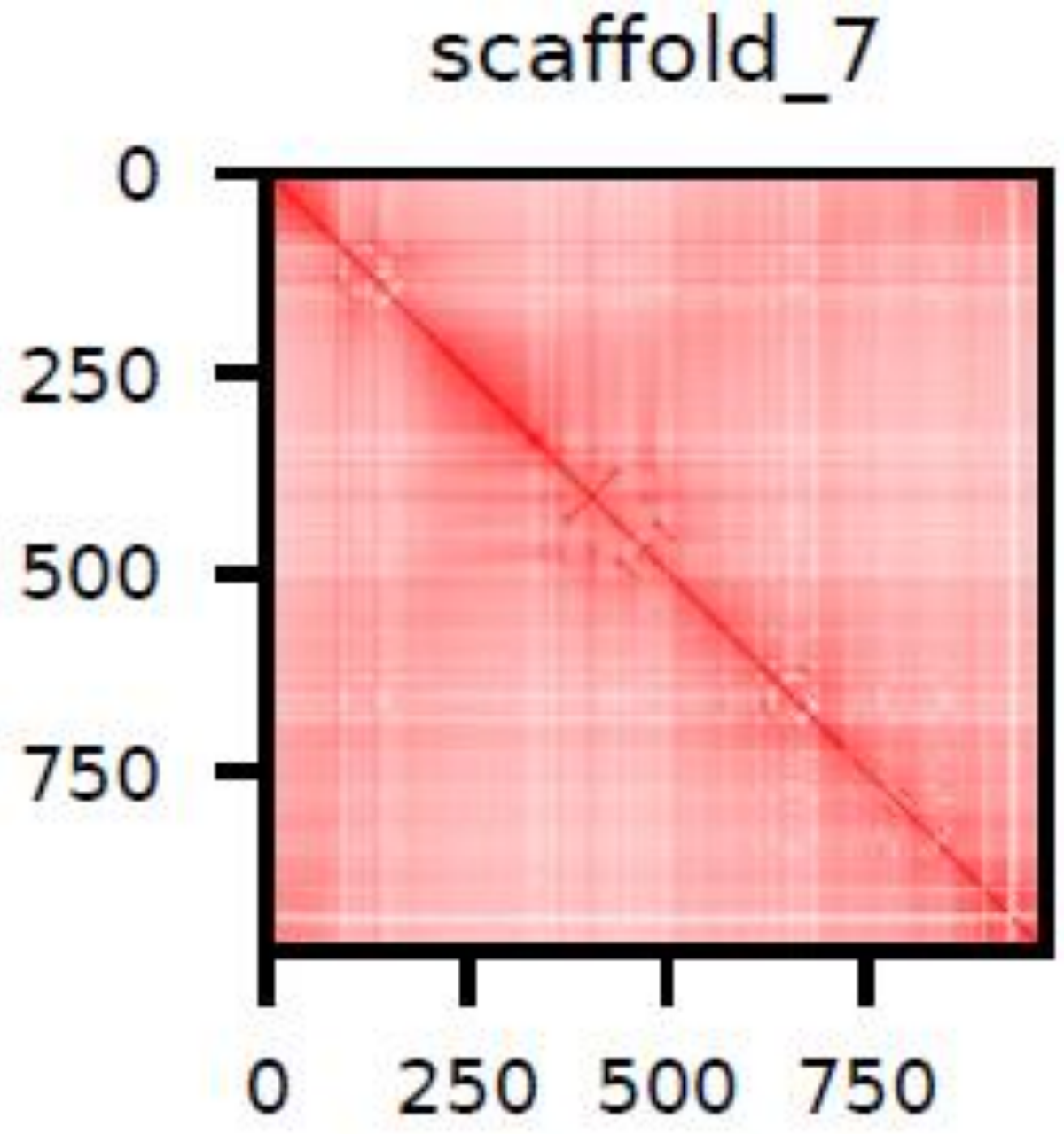

Response Figure 2: HiC interaction plot for the published Weining Hi-C data on the Weining.V2 assembly[1]. a) Genome-wide Hi-C interaction matrix. b) Detailed view of an example for assembly issues.

(2) Phased Genome Assembly and Evaluation of Weining.V3

We download the HiFi, ONT and Hi-C data from publication[1]. We integrated the following assembly pipeline to obtain the Weining.V3 haplotype 1 (H1) and Haplotype 2 (H2) contig groups.

First, after performing quality control on both HiFi, Hi-C and ONT reads, the HiFi reads were used to estimate the heterozygosity rate using software GenomeScope 2.0[4]. Genome assembly was performed following the pipeline previously established for potato[5]. HiFi, ONT and Hi-C reads were processed with hifiasm (v.0.24.0)[6] to generate contig groups such as p_utg, ctg_Hap1, and ctg_Hap2.

Using the TRITEX assembly pipeline[7], https://tritexassembly.bitbucket.io/, the two contig groups generated by the software were separately assembled. During this process, chimeric contigs were broken, ultimately yielding two preliminary draft pseudomolecules, Weining_H1 and Weining _H2. At the same time, we obtained information on contigs that were incorrectly partitioned in hifiasm for both assemblies.

To correct the contig partitioning errors, contigs from the two above assemblies were combined and mapped again with Hi-C reads. Using YaHS[8] and, we generated .hic file. Finally, the mis-partitioned contigs were corrected with the help of Juicebox[9] (https://github.com/aidenlab/Juicebox/wiki), and with the assistance of HapHiC[3], we obtained the haplotype-resolved assemblies along with the Hi-C contact maps (**Response Figure 3 and 4)**.

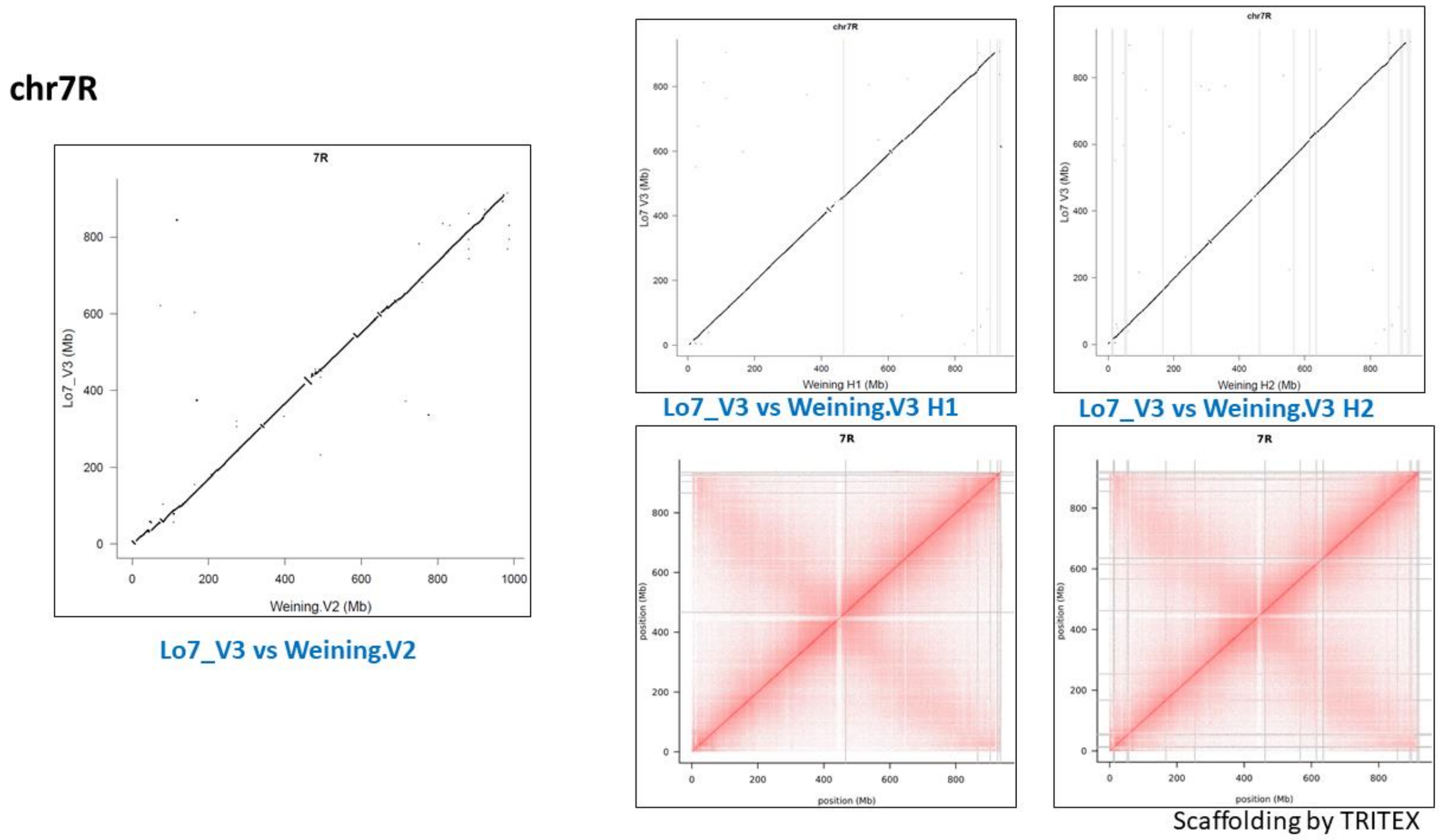

Response Figure 3: Lo7_V3 chromosome 7R was compared by dotplot to Weining.V2 chromosome 7R and both haplotypes H1 and H2 of our newly assembled phased Weining.V3 assembly. Accuracy of the two haplotypes is demonstrated in the Hi-C interaction plots.

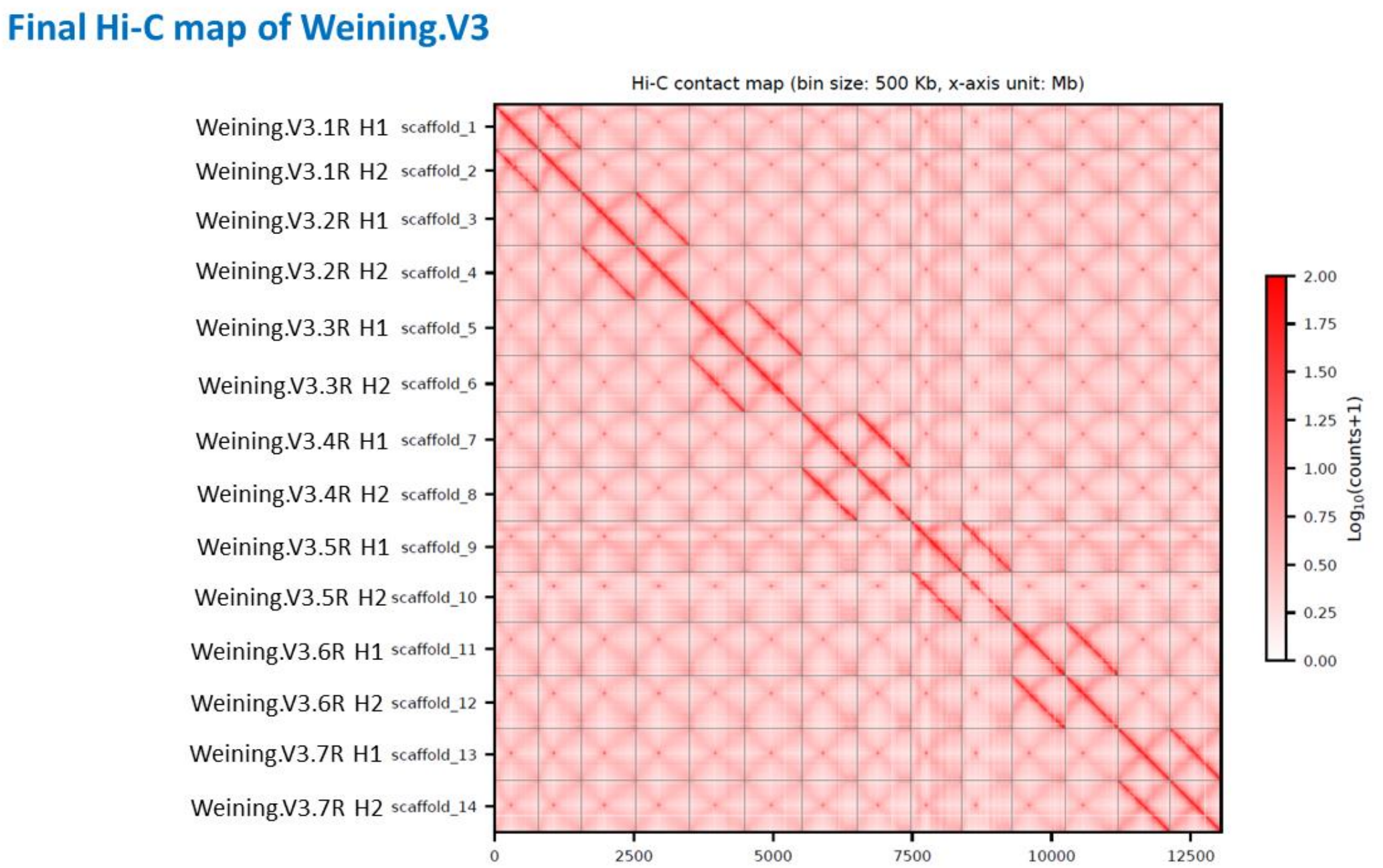

Response Figure 4: Hi-C interaction matrix for the newly generated de novo phased assembly Weining.V3.

Genome completeness (97.7%) and QV (63.5, based on HiFi data) were evaluated using Merqury (v.1.3)[10]. Additionally, BUSCO (Benchmarking Universal Single-Copy Orthologs; v5.8.2)[11] was employed

with the embryophyta_odb10 database to evaluate gene space completeness of the assembly (99.1%). And the average LAI for Weining.V3 H1 and H2 chr1R-7R are 23.93 and 22.91, respectively.

3. Genome characteristics of the phased assembly Weining.V3

Based on the newly generated phased assembly it becomes evident that genome size differences of Lo7 to previous Weining assemblies is mainly attributed by redundancy in the published Weining assemblies due to assembly artifacts. We compared TE (**Response Table 2**) and STR (**Response Figure 5**) statistics, which indicate highly comparable assembly quality.

Response Table 1: Anchored genome sizes between Lo7_V3, Weining.V3 H1 and Weining.V3 H2

	Weining.V1	Weining.V2	Weining.V3 H1	Weining.V3 H2	Lo7_V3
chr1R (Mbp)	941.0	974.0	793.61	786.62	775.56
chr2R (Mbp)	1140.5	1134.9	971.81	973.33	1011.42
chr3R (Mbp)	1094.8	1157.7	992.47	1017.28	1128.39
chr4R (Mbp)	1043.8	1091.4	994.01	963.33	973.44
chr5R (Mbp)	998.9	1259.0	909.02	894.69	924.46
chr6R (Mbp)	1024.5	1106.7	968.58	944.43	943.69
chr7R (Mbp)	1010.0	987.1	934.70	917.79	920.15
Unanchored (Mbp)	484.4	37.3	608		23
In total (Gbp)	7.74	7.75	6.56	6.50	6.75

Response Table 2: TE annotations

	Lo7_V3	Weining.V3 H1	Weining.V3 H2
**Repeat Classes**	**%masked**	**%masked**	**%masked**
LINE_L1	0.24%	0.34%	0.47%
LINE_unknown	0.45%	0.44%	0.45%
SINE	0.24%	0.25%	0.25%
LTR_Copia	14.12%	15.35%	14.76%
LTR_Gypsy	49.34%	49.48%	50.93%
LTR_unknown	9.86%	11.19%	10.85%
TIR_CACTA	4.89%	5.18%	5.13%
TIR_Mutator	0.71%	0.72%	0.80%
TIR_PIF_Harbinger	0.95%	1.06%	0.97%
TIR_Tc1_Mariner	1.24%	1.58%	1.47%
TIR_hAT	0.24%	0.26%	0.29%

TIR_piggyBac	0%	0.00%	0.00%
TIR_unknown	0.01%	0.01%	0.01%
Helitron	1.77%	2.05%	2.56%
Others/Repeat_fragment	5.2%	2.40%	1.78%
Total interspersed	89.26%	90.32%	90.71%

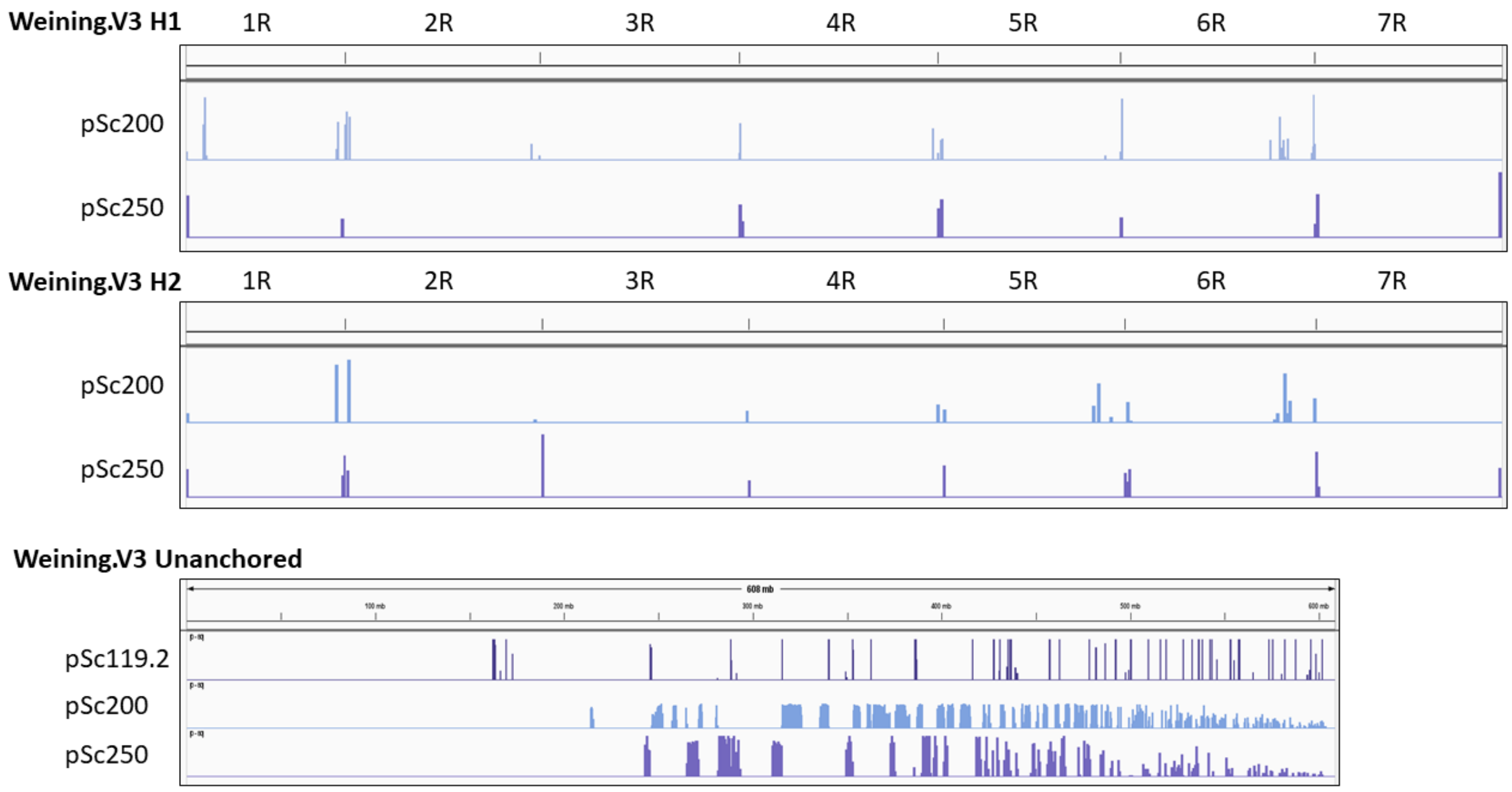

Response Figure 4: STR annotations. We also used prominent STR loci, i.e., pSc119.2, pSc200 and pSc250, to annotate Weining.V3 H1, H2 and unanchored regions. Similar to the Lo7v3 assembly, most of the unanchored DNA sequence is composed of STR families, highlighting that STR loci contribute predominantly to remaining assembly gaps in rye.

4. Centromere identification

We used published Weining CENH3 ChIP-seq data for illustrating the positions of the functional centromeres in haplotypes H1 and H2 (**Response Figure 5**), demonstrating further the assembly quality.

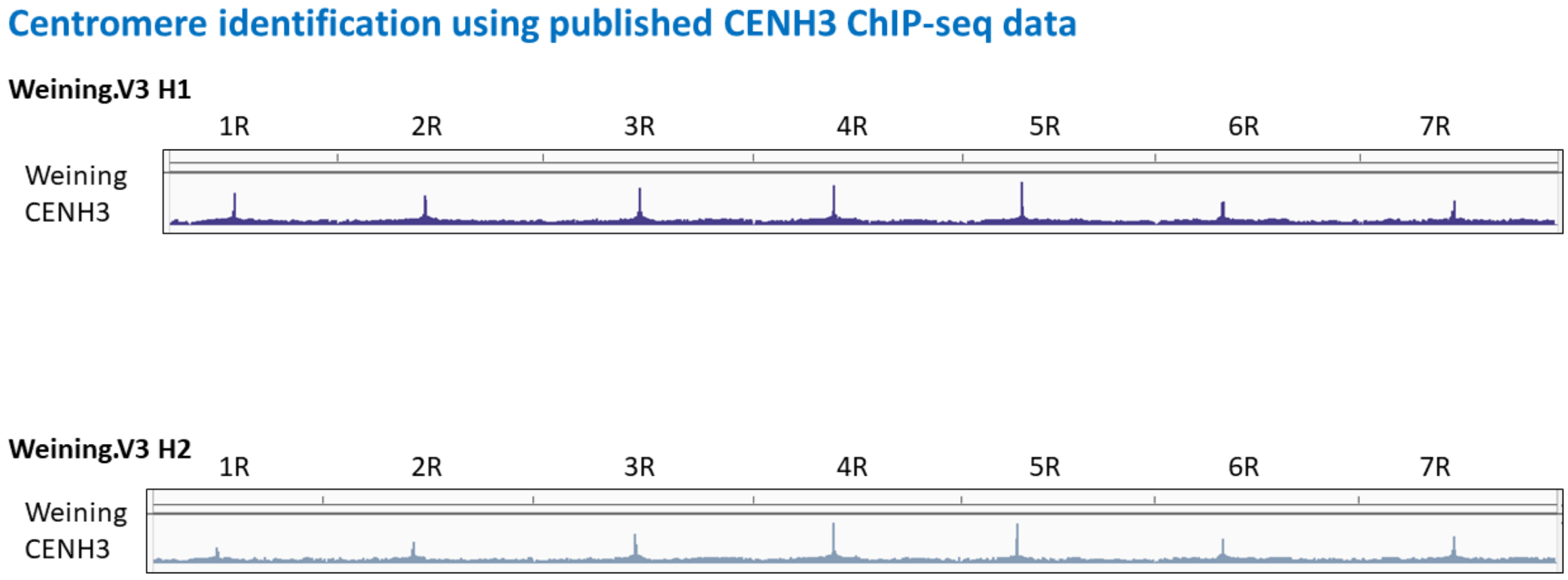

Response Figure 5: Using published Weining CENH3 ChIP-seq data[12], centromeres associated with H1 and H2 were also identified in Weining.V3.

5. Chromosome 7R inversion

Based on the newly generated phased Weining.V3 assembly we revisited the assembly structure at the centromeric region of chromosome 7R. There are two haplotypes within chromosome 7R centromeric region, compared with published Weining.V2 (**Response Figure 6-7**).

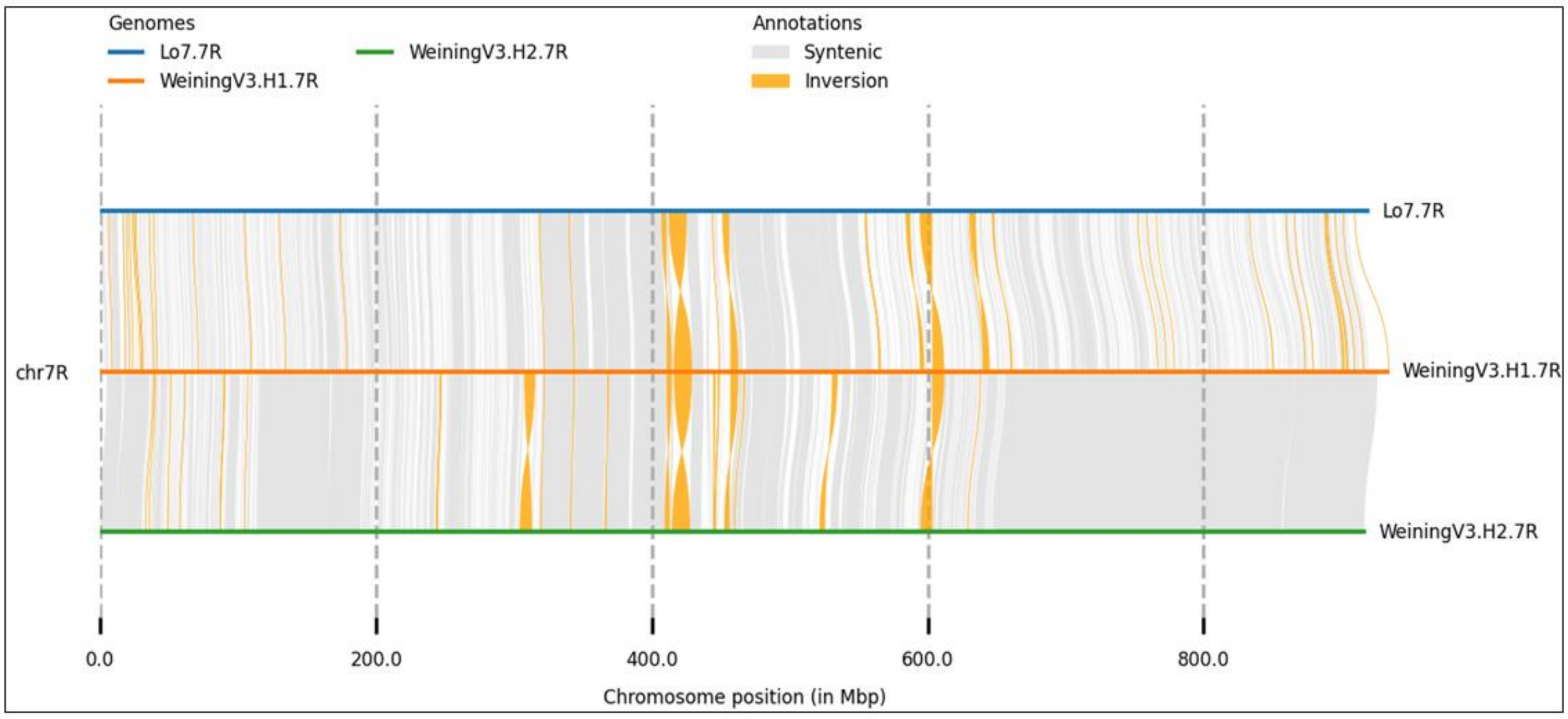

Response Figure 6: Global synteny comparison of Lo7_V3 chromosome 7R vs Weining.V3 7R haplotypes 1 and 2 (H1&H2).

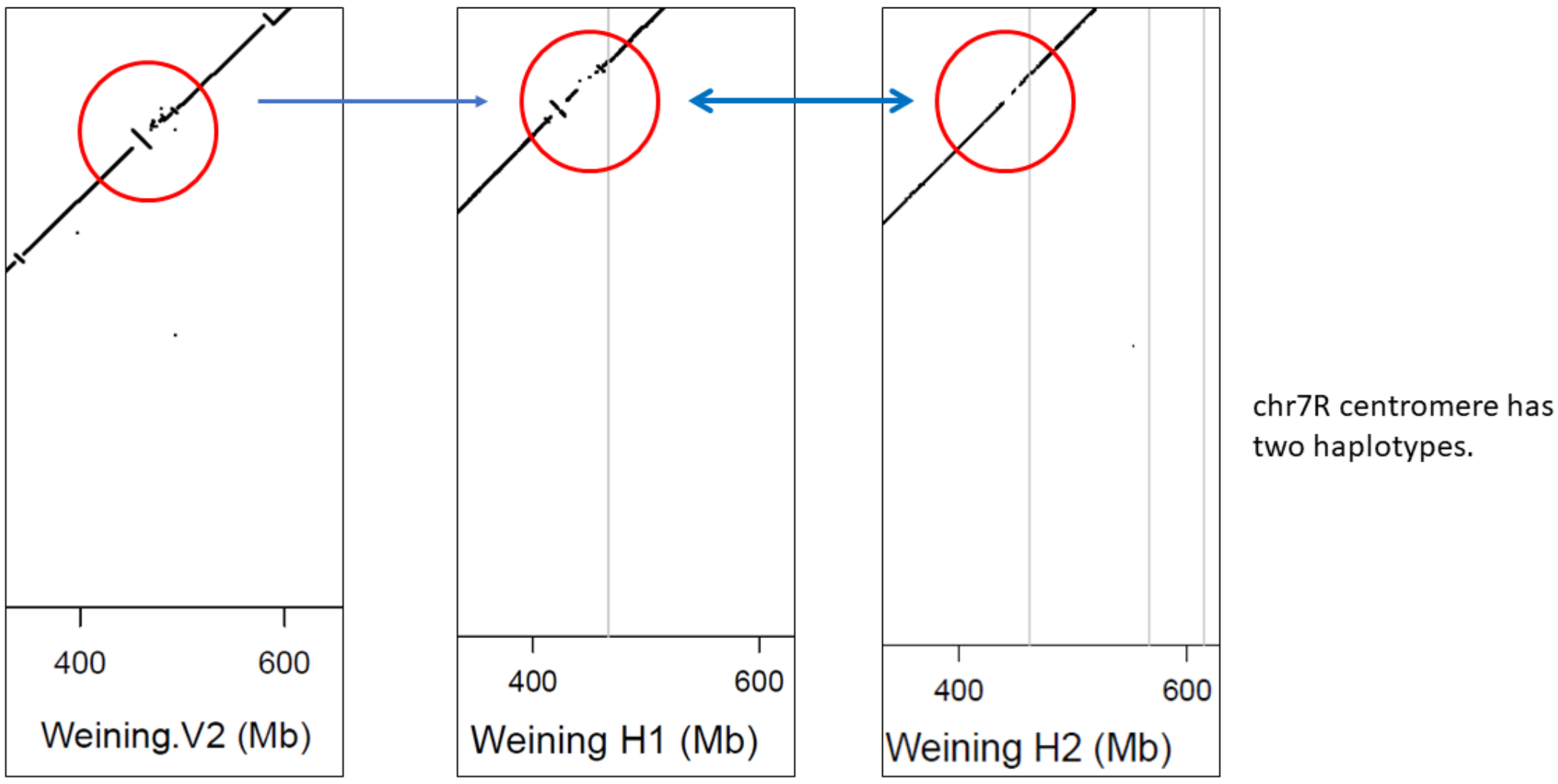

Response Figure 7: Detailed view on the dotplot comparison of Lo7_V3 chromosome 7R (*y*-axis) vs Weining.V2 and Weining.V3 H1 and H2. Weining.V3 H1 carries an inversion compared to Lo7 (Inversion C), while Weining.V3 H2 is mostly colinear to Lo7.

Additionaly, we validated the breakpoints of the inversion C (**Fig.5d**), by examining the optical maps as well as mappings with PacBio HiFi and ONT reads (**Supplementary Fig.29)**. This showed that the entire chromosomal region is covered continuously in all. Additionally, we mapped Illumina short reads and PacBio HiFi reads from Weining on to the Weining.V3 assembly. This also showed numerous reads and read pairs spanning the precise proposed breakpoints in Weining, with no discordant read pairs or clipped reads being present. We show this now in the new **Supplementary Fig.29**. We also examined cross-mappings of Lo7 reads on to Weining. This indeed allowed identification of clipped reads and discordant read pairs precisely at the proposed inversion breakpoints. However, due to the highly repetitive nature of the regions, these were less conclusive and we therefore chose not to show them in the supplementary figure. We emphasize that are extremely confident with the quality of the assembly in this regions, as it also contains no sequence gaps. Interestingly, haplotype H2 of Weining is much more similar to Lo7 as it also contains the inversion C and the entire centromeric region aligns better than between Lo7 and H1. The results of the comparisons are also shown now in **Supplementary Fig. 28**.

3.Assembly completeness and data–claim mismatch. The authors emphasize that all seven centromeres are "fully assembled," yet only 6 of 14 telomeres were detected and 106 gaps remain unresolved. The final assembly size (6.76 Gb) is substantially smaller than cytometric estimates of ~7–8 Gb. The explanations provided—such as differences in standard genome size calibration or the collapse of STRs—are qualitative and unsupported by quantitative breakdowns of missing sequence classes. Similarly, the justification for the adequacy of ~20× Hi-C depth in a 7 Gb repeat-rich genome is based on empirical experience rather than any quantitative modeling or curve-fitting analysis demonstrating coverage sufficiency across repetitive regions. As a result, the claim of a "near-complete" or "complete centromere" assembly overstates what is currently achieved.

Response: We kindly disagree with the reviewer. We would like to emphasize that the Lo7_V3 genome assembly was extensively validated in addition to Hi-C–based scaffolding by incorporating **a newly generated high-coverage (190×)** optical genome map, hence, reviewer comments about us overstating completeness are not justified. Instead, we are transparent about the remaining incompleteness and assembly gaps, which, however, are mostly attributable to highly repetitive, subtelomeric STR loci (e.g., pSc119.2, pSc200, and pSc250 and others), which have likely also affected the incompleteness of the telomeric regions.

Regarding genome size estimations, we have clearly demonstrated (i) by reassembly of the recently published Weining data[1], leading to a de novo phased version 3 of the Weining genome (Weining.V3), as well as (ii) by extensive comparisons of Lo7_V3 against the published Weining.V1 and V2 assemblies, that earlier Weining assemblies overestimate genome size due to redundant haplotype representation.

We repeated genome size estimation by flow cytometry experiments and calculated the 1C values based on two different published references (**Supplementary Table 2**).

**Response Table 3**: Flow cytometric measurement of DNA content		
	**Lo7***	**Lo7****
**Genome size (Mbp/1C)**	8037*	7064**
*Dolezel et al. 1998 (doi: 10.1006/anbo.1998.0730) – *Pisum sativum*: 9.09 pg/2C determined assuming a 2C DNA value of 7.0 pg for human (Tiersch et al., 1989)<br>**Soni and Henry 2025 (doi: 10.3389/fpls.2025.1548766) – *Pisum sativum*: 7.99 pg/2C, recalculated estimate using the updated human male genome (2C = 6.15 pg) (Nurk et al., 2022)		

Based on a recently recalculated value of 7.99 pg/2C for the reference standard *Pisum sativum* (Soni and Henry, 2025)[13], we show that the genome size predicted by flow cytometry (~7.064 Gbp) is smaller than previously assumed. The new value is more consistent with the length of the anchored sequences (~6.75 Gbp) presented in this study for Lo7 and the two haplotypes of Weining.V3. The haploid genome size of 8 Gbp for rye is likely an overestimate.

4. Questionable interpretation of RLG_Abia activity differences between rye and wheat. The authors reannotated TEs using a unified library and added RNA-seq expression comparisons between rye and wheat. Nevertheless, the conclusion that RLG_Abia and RLG_Abigail are "highly active" in rye but "suppressed" in wheat is still weakly supported. The analysis remains vulnerable to annotation bias, inconsistent RNA-seq datasets, and lack of population-level validation. No transposition polymorphism (TIP) evidence, recent insertion signatures, or molecular intermediates are provided to demonstrate genuine activity. Hence, the observed differences could simply reflect assembly and annotation quality rather than true biological divergence.

Response: To address the reviewer's comments we performed detailed comparative analyses of the centromeric and peric-centromeric regions between Lo7 and Weining haplotypes H1 and H2. We aligned the centromeric ~10 Mb plus ~35 Mbp of pericentric regions between Lo7 and H2 (the more closely related haplotype). From the alignment of the regions, we estimate that Lo7 and H2 diverged probably only about 6,300 years ago. In contrast Lo7 and H1 are more divergent (see also response to

comment 2 above). Here, the centromeres could not be aligned, but we aligned ~20 Mb of pericentric regions, from which we then estimated a divergence time of ~70,000 years. This data is shown in the **Supplementary Fig. 30**.

We then searched the aligned regions in order to identify TE insertion polymophisms (TIPs). All candidate TIPs were stringently evaluated (i.e the inserted element had to be full-length with intact ends and it had to be flanked by the typical 5 bp target site duplication. We identified 143 TIPs between Lo7 and H2. Of these, 134 had insertion age zero (i.e. identical LTR). Thus, they must have inserted in the past ~6,300 years since Lo7 and H2 diverged. 121 of the TIPs were inside the predicted functional centromere (**Supplementary Fig. 30**), 73 of them are *Abia* and 59 *Abigail* TE. In our opinion, this is strong evidence for recent high activity. We now refer to these results in the main text (Line **307**). However, to address the reviewer's concern, we toned down the language stating that they were highly active very recently, while we have no evidence for recent activity in wheat (results and discussions, Lines **381**).

This analysis also assesses the assembly quality, since the identified TIPs all show the precise characteristics that are expected (i.e., full-length seemingly intact copies that are precisely flanked by the typical 5 bp target site duplication. Additionally, the identified TIPs are highly enriched in the predicted active centromere, while they are rare outside centromeres. Furthermore, the insertions time estimates are completely consistent with overall divergence times of H2 and H1 with almost all TIPs in H1 having insertion age zero. Moreover, the pericentric regions between Lo7 and H2 (divergence ~6,300 years) show 4 TIPs while between Lo7 and H1 (divergence ~70,000 years), we find 34 TIPs. The procedures are described in a new methods paragraph and source data and detailed descriptions are provided at the GitHub repository https://github.com/Wicker-Lab/Rye_centromere_analysis).

Thus, we are extremely confident that the Lo7 assembly is of very high integrity (and Weining.V3 H1 and H2 to similar degrees) and that our observations reflect true biological differences.

In summary, while the revised manuscript presents an improved genome assembly, it does not resolve the central conceptual and evidential issues identified previously. The work remains incremental, with key claims inadequately supported by independent validation. I therefore recommend rejection of this manuscript for Nature Communications. The dataset could be more appropriately published as a technical resource after further validation and expansion of biological insight.

Response: We truly thank the reviewer for his/her critical comments and the extensive efforts made in reviewing our manuscript repeatedly, and believe that our attempts to address them has substantially improved our study.

Reviewer #2 (Remarks to the Author):

The authors have properly addressed all my concerns, and I have no further questions.

Response: Thank you very much!

Reviewer #3 (Remarks to the Author):

I thank the authors for their revisions and for their response to my comments. I have reviewed the revised manuscript and find it substantially improved, with most of my previous comments addressed. I have only a small number of remaining points:

1. Line 180: The authors should clarify what is meant by the term "functional" centromere here. Does this refer specifically to the CENH3-enriched region (spanning approximately 10–12 Mb, Supplementary Table 8), or is this being conflated with the full centromeric retrotransposon array? A 10–12 Mb region seems quite large for a functional centromere, even in comparison to large genomes like wheat.

Looking at the CENH3 analysis it is not stated whether the ChIP-seq mapping includes multi-mapping reads. If multi-mappers are included, this could substantially inflate the apparent size of the centromeric domain due to the repetitiveness of the TEs. I would recommend estimating upper and lower bounds of centromere size using two mapping strategies:
1) multiple + unique mapping to provide a maximum (upper bound) estimate this is likely the reported 10-12Mb, and
2) unique-only mapping only to provide a minimum (lower bound) estimate.
This approach has been used successfully in other species (e.g. https://doi.org/10.1111/tpj.70153, https://doi.org/10.1371/journal.pgen.1000743) to provide a range on which the likely functional centromere occupies

Response: We are grateful for the insightful suggestions. We used now different mapping quality thresholds (MAPQ0 and MAPQ30) and estimated the maximum and minimum (multiple + unique mapping) centromere size of rye Lo7 (**Response Table 4 and 5, respectively**). Based on these results we conclude that, although larger than in wheat, our reported centromere sizes are not strongly overestimated. We included this additional data in the **Supplementary Table 11**.

Response Table 4: Centromere size estimation using CENH3 ChIP-seq data mapping at MAPQ0 quality threshold

Centromeres	Position_Sart	Position_End	Length
1R	289,857,897	314,916,269	25,058,372
2R	433,868,872	458,034,649	24,165,777
3R	523,258,072	540,395,139	17,137,067
4R	375,162,173	404,590,119	29,427,946
5R	278,508,655	301,385,042	22,876,387
6R	303,224,529	326,693,863	23,469,334
7R	428,672,858	459,009,684	30,336,826

Response Table 5: Centromere size estimation using CENH3 ChIP-seq data mapping at MAPQ30 quality threshold

Centromeres	Position_Sart	Position_End	Length
1R	294,894,686	305,528,973	10,634,287
2R	440,193,008	451,759,380	11,566,372
3R	525,992,185	537,137,158	11,144,973
4R	383,457,614	394,737,003	11,279,389

5R	286,328,000	295,889,973	9,561,973
6R	308,033,807	319,335,901	11,302,094
7R	436,325,765	447,617,757	11,291,992

2. Nanopore does provide a tool for calling methylated cytosines, and this can be done through Dorado basecaller using R10 data their integrated modified base models (see below). While I understand that the assembly subset (>25 kb reads) may only provide ~9× coverage, relaxing this threshold to >5–10 kb would likely yield higher coverage sufficient to generate a useful genome-wide methylation profile, which would be a valuable addition to the manuscript.

If this still doesn't provide sufficient coverage for this analysis, the basecalling should be performed using the dorado dna_r10.4.1_e8.2_400bps_sup@v5.2.0 with the 5mC_5hmC modified base model, and that the resulting BAM file can provided to the community. This is important, because methylation cannot be re-called at a later date as the authors suggested without the pod5 files, which are not currently available through the ENA under project ID PRJEB91463 (based on the methods the current provided .bam file wont contain the methylation tags for the community).

Response: Again, we would like to thank the reviewer for these constructive suggestions. We made the ONT raw pod5 files publicly available through submission to ENA, PRJEB108142 (https://www.ebi.ac.uk/ena/browser/view/PRJEB108142) to allow for such an analysis, which is, however, heavily compute-resource intense. As an alternative, we generated de novo Whole Genome Bisulfite Sequencing data (WGBS) for Lo7 to perform such an analysis and demonstrated the quality of the generated data by illustrating three different methylation contexts (CG, CHG, and CHH) in a genome-wide manner (**Response Figure 9a**) and in a gene- or TE-context, respectively (**Response Figure 9b**), and included these in (**Supplemental Fig. 20**). Based on the above analyses, we further highlighted the centromeric regions in the Lo7 chromosomes and identified seven CHG dips in the centromeres relative to the flanking regions (**Supplemental Fig. 20**), a pattern very similar to that observed in *Arabidopsis*[14].

a)

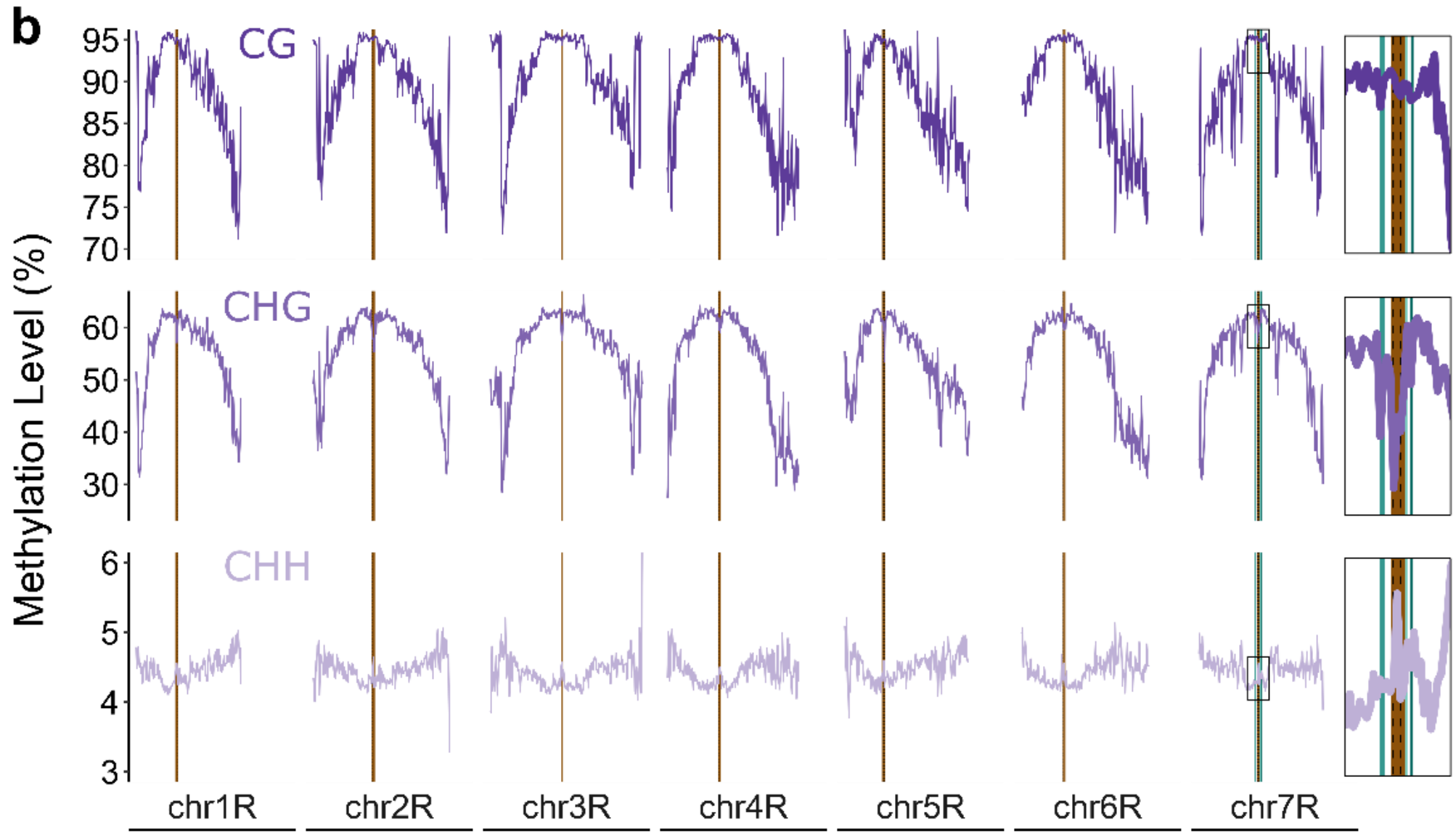

b)

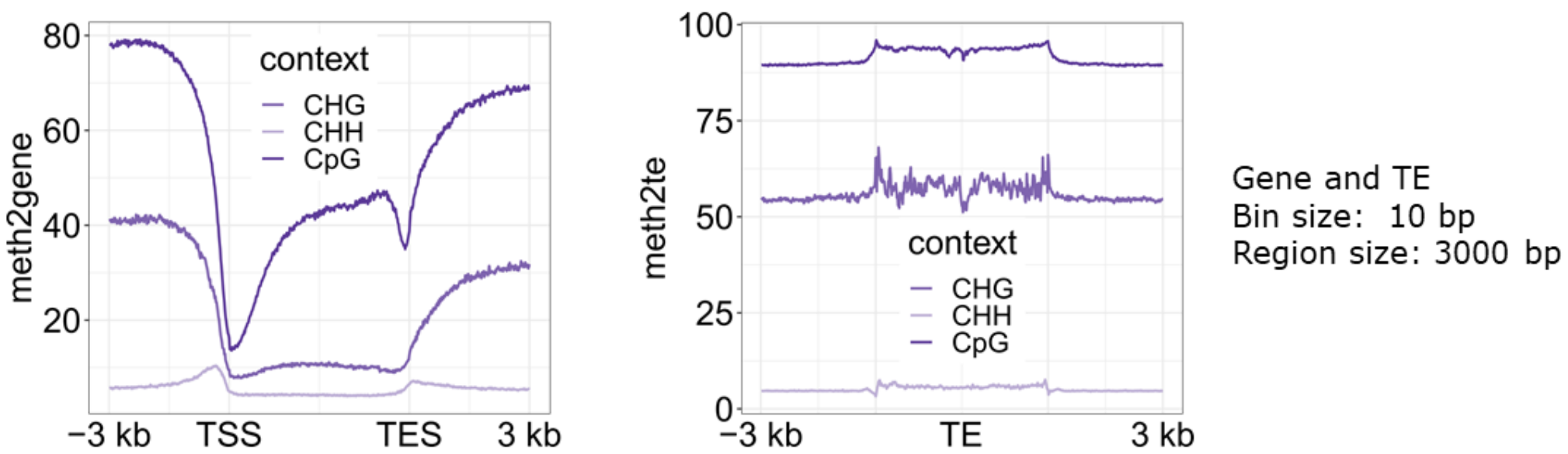

Response Figure 9: WGBS based profiling of DNA methylation of the Lo7_V3 genome assembly

References:

1 Yi, C. *et al.* High-resolution genome assembly reveals retrotransposon-mediated centromere dynamics in rye. *Genome Biol* **26**, 308, doi:10.1186/s13059-025-03792-3 (2025).

2 Li, G. *et al.* A high-quality genome assembly highlights rye genomic characteristics and agronomically important genes. *Nat Genet* **53**, 574-584, doi:10.1038/s41588-021-00808-z (2021).

3 Zeng, X. *et al.* Chromosome-level scaffolding of haplotype-resolved assemblies using Hi-C data without reference genomes. *Nature Plants* **10**, 1184-1200, doi:10.1038/s41477-024-01755-3 (2024).

4 Ranallo-Benavidez, T. R., Jaron, K. S. & Schatz, M. C. GenomeScope 2.0 and Smudgeplot for reference-free profiling of polyploid genomes. *Nat Commun* **11**, 1432, doi:10.1038/s41467-020-14998-3 (2020).

5 Cheng, L. *et al.* Leveraging a phased pangenome for haplotype design of hybrid potato. *Nature*, doi:10.1038/s41586-024-08476-9 (2025).

6 Cheng, H., Asri, M., Lucas, J., Koren, S. & Li, H. Scalable telomere-to-telomere assembly for diploid and polyploid genomes with double graph. *Nat Methods*, doi:10.1038/s41592-024-02269-8 (2024).

7 Marone, M. P., Singh, H. C., Pozniak, C. J. & Mascher, M. A technical guide to TRITEX, a computational pipeline for chromosome-scale sequence assembly of plant genomes. *Plant Methods* **18**, 128, doi:10.1186/s13007-022-00964-1 (2022).

8 Zhou, C., McCarthy, S. A. & Durbin, R. YaHS: yet another Hi-C scaffolding tool. *Bioinformatics* **39**, doi:10.1093/bioinformatics/btac808 (2023).

9 Durand, N. C. *et al.* Juicebox Provides a Visualization System for Hi-C Contact Maps with Unlimited Zoom. *Cell Syst* **3**, 99-101, doi:10.1016/j.cels.2015.07.012 (2016).

10 Rhie, A., Walenz, B. P., Koren, S. & Phillippy, A. M. Merqury: reference-free quality, completeness, and phasing assessment for genome assemblies. *Genome Biol* **21**, 245, doi:10.1186/s13059-020-02134-9 (2020).

11 Manni, M., Berkeley, M. R., Seppey, M. & Zdobnov, E. M. BUSCO: Assessing Genomic Data Quality and Beyond. *Curr Protoc* **1**, e323, doi:10.1002/cpz1.323 (2021).

12 Liu, C. *et al.* Unveiling the distinctive traits of functional rye centromeres: minisatellites, retrotransposons, and R-loop formation. *Sci China Life Sci*, doi:10.1007/s11427-023-2524-0 (2024).

13 Soni, A. & Henry, R. J. Re-calibration of flow cytometry standards for plant genome size estimation. *Frontiers in Plant Science* **Volume 16 - 2025**, doi:10.3389/fpls.2025.1548766 (2025).

14 Naish, M. *et al.* The genetic and epigenetic landscape of the Arabidopsis centromeres. *Science* **374**, eabi7489, doi:10.1126/science.abi7489 (2021).

All revised content of the main manuscript, supplementary figures, and supplementary tables has been highlighted in yellow throughout to facilitate tracking.

Rebuttal REVIEWER COMMENTS

Reviewer #1 (Remarks to the Author):

The authors have clearly invested considerable effort in improving the manuscript. The additional analyses have improved the manuscript, especially with respect to assembly validation, re-analysis of centromeric regions, and the comparison with the Weining datasets. I appreciate the amount of work that went into the revision.

Reply:

We thank the reviewer for the comment.

That said, after reading the revised manuscript and rebuttal carefully, I remain unconvinced that the main concerns have been fully resolved. In my view, the study is still centered primarily on an improved rye genome assembly and a more detailed description of centromeric retrotransposons, but it does not yet provide enough conceptual or mechanistic advance to justify publication in Nature Communications.

Reply:

We thank the reviewer for the comment.

My main concerns are the following.
First, the overall biological advance remains limited. The revised manuscript now provides a stronger assembly resource and a richer description of centromeric TE composition and distribution, but the interpretation is still largely descriptive. In particular, the study does not yet establish clearly how the observed TE patterns translate into a deeper mechanistic understanding of centromere function, centromere repositioning, or chromosome evolution. For this reason, the manuscript still reads more like a resource and comparative genomics study than a paper with a clear conceptual advance.

Reply:

The reviewer is right, our manuscript provides a significantly improved genomic resource for rye, which is removing a substantial proportion of the remaining limitations of previously published rye genome assemblies, thus paving the way for highly advanced functional genomic and comparative genomic analyses in rye, the *Triticeae* and beyond. We are also transparent about the remaining challenges in rye genome assembly, that to our opinion still will require additional improvements in technology before complex STR loci will be amenable to full assembly.

The reviewer is criticizing the descriptive nature of the manuscript. This is, however, a recurring notion of experts, largely ignoring that genomics per se is often a rather descriptive research approach that, nonetheless, has massively stimulated biological and mechanistic discovery and hypothesis building/testing. The reviewer sees a shortcoming in the way we report TE patterns in the

centromere without contributing to the mechanistic understanding of centromere function. With respect, this was not our target. Instead we have documented at unprecedented detail and resolution the traces of frequent and recent inversions in rye centromeres, which to our opinion fundamentally contribute to the observed rapid loss of collinearity in centromeric and pericentric regions of the *Triticeae* genomes and thus may contribute to speciation.

Second, the evidence for the proposed 7R model remains uneven. The additional comparison with the phased Weining reassembly strengthens the case for the most recent event, inversion C, and provides more convincing support through approximate breakpoint definition and read-based validation. However, the older proposed events are still inferred mainly from TE age-position patterns rather than from comparable structural evidence. The rebuttal itself acknowledges that exact breakpoints cannot be provided for these older events, yet the Results section still presents a relatively strong three-inversion scenario with temporal assignments. I think the current data support the conclusion that the peri-centromeric region of 7R has undergone structural rearrangement, with one recent event being relatively well supported, but the full three-step model remains at least partly speculative.

Reply:

We thank the reviewer for the comment. We have modified the language in the respective sections to make clear to the reader that we provide very strong evidence for one recent event and that we use this as supportive evidence to infer analogous, but older, events on the same or other chromosomes, keeping in mind that this may not rule out other possible explanations.

In response to the reviewer we toned down the language in the mentioned results part and emphasized that we describe a model based on TE age-distribution patterns with one of the proposed inversions, inversion C, additionally being well supported by comparison to a haplotype not carrying said inversion (following Line 266). Further, we added a sentence clearly stating that the two older proposed inversion events are inferred only from TE age-distribution patterns in the absence of haplotypes which do not contain these proposed inversions (following Line 288).

Third, some of the wording in the revised manuscript is still stronger than the data support. This is particularly noticeable in the discussion of RLG_Abia and RLG_Abigail activity. In the rebuttal, the authors state that they toned down the language and that there is no evidence for recent activity in wheat. However, the manuscript still states that these families were "silent for a long time" in wheat and are "currently highly active" in rye. This goes beyond the more limited conclusion that recent activity is supported in rye but not demonstrated in wheat. I think this section should be phrased more cautiously.

Reply:

In response to the reviewer's comment, we added the median insertion ages and ages of the youngest TE copies for each TE familiy and species/subgenome to the graph in Supplementary Fig. 31. We also now use more precise and cautious language, referring to the median insertion ages of the TE copies, and stating that these indicate that the *RLG_Abia* and *RLG_Abigail* elements were silent in wheat. Similarly, we refer to the low median insertion ages as indication that these two

families have recently been highly active, (and may still be) in rye (Line 302). We also revised the respective statement in the discussion accordingly (following Line 372).

Fourth, I am concerned by inconsistencies between the rebuttal and the revised manuscript in the reporting of the TE insertion polymorphism results. The numerical summaries do not appear fully consistent, and part of the rebuttal summary also seems internally inconsistent. These issues may reflect revision-stage updating, but they should be reconciled clearly, as they weaken confidence in an analysis that is now central to the revised argument.

Reply:

We thank the reviewer for checking this carefully and alerting us about any possible inconsistencies that may have occurred during the revision process. We have carefully reviewed all sections of the main manuscript and the supplements to remove any obvious remaining inconsistencies.

The numbers provided were indeed an oversight, as we used some placeholder numbers which we did not update by accident (in the rebuttal: 143 instead of 147, and 121 instead of 114; and in the main text: 66 instead of 63 and 51 instead of 47). In addition, we wrongly stated in the rebuttal letter that 73 and 59 polymorphic insertions (TIPs) of *RLG_Abia* and *RLG_Abigail*, respectively, were found in centromeres. This wrongly referred to all TIPs of the two families identified in the analysed centromeric and peri-centromeric regions. The correct numbers are 63 and 47, respectively. These were also corrected in the main text (Line 306).

We also carefully checked the original data again. The data used for Supplementary Fig. 30 is correct and corresponds to what was deposited on our GitHub repository https://github.com/Wicker-Lab/Rye_centromere_analysis). We thank the reviewer for the careful examination of the numbers.

Fifth, the discussion of the published Weining.V2 assembly should be more balanced. I agree that unresolved haplotypic redundancy in a more heterozygous assembly may complicate direct fine-scale comparison with the highly homozygous Lo7 assembly, and I understand why the authors generated a phased reassembly for this purpose. However, the current manuscript tends to treat this as evidence of broadly reduced assembly quality or structural flaws in the published Weining.V2 assembly. In my view, the analyses presented here support the conclusion that Weining.V2 is not ideal for this specific benchmarking purpose, but they do not support a broader assessment of its overall quality. This distinction should be made more clearly.

Reply:

We agree with the reviewer that it is important to present the reanalysis of the Weining data in a balanced, neutral and unbiased way. Our fine-scaled comparisons to Weining V2 led to the observation of frequent redundancy throughout the genome assembly present on all chromosomes, leading in essence also to an inflated genome size estimation for Weining. This observation raised our concerns that fine-scaled comparisons with Weining V2, because of the observed structural limitations, might lead overall to unjustified conclusions. We therefore tested the possibility to improve the assembly using the very same data for phased assembly and evidently preferred to continue our comparisons with the newly derived phased assembly for further comparisons. We have revised the language of the respective section reporting these results to avoid the impression of any unintentional biased or tendentious interpretation.

Sixth, the completeness issue is still not fully addressed quantitatively. The revised flow-cytometric estimate is useful and does make the reported assembly size more plausible. However, the remaining discrepancy is still explained mainly in qualitative terms, for example by invoking subtelomeric tandem repeats and repeat-rich unresolved regions. In addition, Supplementary Fig. 5 appears to show several localized windows with elevated depth in both HiFi and ONT data despite relatively stable GC content. This pattern could be consistent with local collapsed or over-compressed repetitive sequence representation, yet these outlier regions are not quantified or discussed in detail. It would be important to estimate their genomic span, determine whether they are enriched for particular repeat classes, and assess how much of the remaining assembly-length discrepancy they may explain.

Reply:

We thank the reviewer for this important suggestion. To quantitatively evaluate the potential contribution of locally collapsed repetitive regions to the residual assembly-size discrepancy, we performed a genome-wide coverage analysis using both HiFi and ONT read alignments in non-overlapping windows. Regions showing significantly elevated read depth relative to the genome-wide median were identified as candidate locally collapsed repetitive regions. These regions collectively span approximately 327 Mb (based on HiFi read depth analysis; including chrUn region), accounting for ~4.8% of the Lo7_V3 assembly. This proportion closely matches the fraction of "repeat fragments" annotated by EDTA-based repeat annotation (5.2%), indicating that a substantial portion of the assembly-size discrepancy is attributable to unresolved or collapsed repetitive sequences (following line 353).

We performed the quantification of 45S rDNA by analyzing tandem repeats in single-molecule optical mapping data (provided in the revised Supplementary Table 3). Our results indicate that the cumulative length of tandemly organized 45S rDNA arrays is approximately 20 Mb, which is in line with reported rDNA array sizes in related grass species such as wheat and barley, supporting the validity of our estimation. A BLAST search for rDNA units in ChrUn revealed that substantial part (~12 Mb) of the rDNA had been assembled in unanchored contigs. Given that additional 2.34 Mb of rDNA are included in the 1R pseudomolecule, the missing 45S rDNA accounts for only 5-6 Mb.

To clarify the interpretation of the genomic regions showing elevated HiFi and ONT read depth but relatively stable GC content, we note that similar patterns have been widely reported in recent high-quality long-read genome assemblies, including the telomere-to-telomere human genome assembly (CHM13) (DOI:10.1126/science.abj6987). In that study, read depth deviations were shown to be specifically enriched in highly repetitive regions such as tandem repeats and satellite DNA arrays, while GC content remained largely unaffected, indicating that GC stability alone is not sufficient to exclude assembly collapse in repetitive regions. Importantly, the assembler 'hifiasm' may still exhibit local overcompression or collapse in highly repetitive sequences despite subsequent polishing and purging steps. To further assess the magnitude of potential missing content, we integrated an independent genome size estimate obtained from flow cytometry (7.06 Gb) (revised Supplementary Table 2). In comparison, the assembled genome size of Lo7_V3 is 6.76 Gb, resulting in an estimated discrepancy of approximately 300 Mb, suggesting that a substantial proportion of the assembly-size discrepancy is attributable to unresolved or collapsed repeat-rich genomic regions (as we added these in the discussion, line 356).

In addition, we systematically analyzed chrUn in conjunction with HiFi (average coverage: 71×, 100 kb windows), ONT (11×, 100 kb windows), and GC content (42%, 1 Mb windows) (provided in the revised Supplementary Table 5 and the additional panel b for Supplementary Fig. 5). The black line represents the simulated sequencing coverage distribution. Collapsed contig groups identified in this region are associated with elevated read depth but stable GC content, indicating that they are primarily driven by repeat complexity rather than compositional biases such as GC content. We further performed TRF-based satellite repeat annotation on the chrUn region, which revealed a strong enrichment of tandem repeats and prominent satellite sequences, indicating that this region consists of highly complex, repeat-rich sequences that are refractory to accurate genome assembly (provided in the new Supplementary Table 17).

Finally, the Discussion still goes somewhat beyond what is directly supported by the data. For example, some statements are framed in terms of a broader retrotransposon-mediated mechanism of centromere evolution or long-term competition for the centromeric niche. These are interesting ideas, but I do not think the present analyses support them strongly enough to present them as conclusions.

Reply:

In response to the reviewer we toned down the language to more clearly state this as a hypothesis and to reflect the coexistence of different retrotransposons in the centromeres of various grasses, across a long evolutionary timeline (following line 373).

Overall, the revision improves the manuscript technically, but I do not think it fully resolves the main concerns regarding conceptual advance and support for the strongest claims. For these reasons, I do not think the manuscript reaches the threshold for publication in Nature Communications.

Reply:

We are grateful to the reviewer for providing highly valuable critical comments throughout the entire reviewing process, which have allowed us to improve the presentation of our research.

Rebuttal to REVIEWERS' COMMENTS

Reviewer #1 (Remarks to the Author):

I have read the revised manuscript and the rebuttal carefully. The authors have addressed several technical points. The assembly validation is clearer, the comparison with the Weining datasets is more useful, and the revised analysis of the centromeric regions is improved.

However, my main concern remains. The revision has not substantially changed the nature of the study. The manuscript still reads primarily as an improved rye genome assembly, with a descriptive survey of centromeric retrotransposon composition, distribution, and insertion-age patterns. These are useful data, but I do not think they amount to a sufficiently clear conceptual advance for Nature Communications.

The strongest biological claim concerns centromere-associated structural evolution, especially the proposed three-step inversion model on chromosome 7R. The additional Weining comparison strengthens the case for the most recent event, inversion C. In contrast, the older proposed events are still inferred mainly from TE age-position patterns rather than from comparable structural evidence. The current data support the conclusion that the 7R pericentromeric region has undergone structural rearrangement, but I do not think they support the full three-inversion model with the confidence and temporal resolution suggested in the manuscript.

Response: We adjusted the section by using more cautious language and by highlighting more clearly that our evolutionary model is a hypothesis based on the physical location and insertion times of TE clusters. We also more clearly emphasize the fact that comparisons to other genomes (haplotypes) which do not carry the proposed inversions would be needed to provide further evidence for this hypothesis of structural rearrangements (Line 298).

I also remain concerned that some statements are still stronger than the data justify. For example, the discussion of RLG_Abia and RLG_Abigail would be better framed as evidence for recent accumulation and possible ongoing activity in rye, rather than as direct evidence that these elements are currently highly active. Similarly, the lack of comparable recent insertions in wheat should not be presented too strongly as long-term silence without additional supporting evidence.

Response: We have further toned down the language in the results (Line 309), and now simply state in the discussion that the high numbers and recent insertion ages of *RLG_Abia* and *RLG_Abigail* elements indicate recent and potentially rye-specific activity of these elements (Line 389).

The completeness of the assembly also limits the resource argument. The assembly is clearly improved, but it is not telomere-to-telomere, several subtelomeric regions remain unresolved, and the authors still report an assembly-size discrepancy consistent with collapsed or unresolved repeat-rich regions. For a resource-centered manuscript at this journal level, either the assembly would need to be exceptionally complete, or the biological conclusions drawn from it would need to be substantially stronger.

Finally, parts of the Discussion still go beyond what is directly demonstrated. The proposed retrotransposon-mediated mechanism of centromere evolution and the idea of long-term competition for the centromeric niche are interesting hypotheses, but the current study does not establish them as conclusions.

Response: We agree that the current study does only imply such niche competition. Although another recent study (which we cite) does use the term competition, we use the more neutral term "coexistence" in the discussion (L388).

Overall, the revision improves the manuscript, but it does not resolve the central issue. I still do not think the manuscript reaches the threshold for publication in Nature Communications.
