# Supplementary material for: Unveiling centromeric retrotransposon dynamics through a high-quality rye genome assembly
Source: Nat Commun. 2026 Aug 15;17:8413. doi: 10.1038/s41467-026-76753-4 (PMC13477480; doi:10.1038/s41467-026-76753-4)
Supplement: Supplementary file 4 [file 41467_2026_76753_MOESM3_ESM.pdf]

Description of Additional Supplementary Files

File Name: Supplementary Data 1

Description: HiFi and ONT sequencing coverage and GC content of the Lo7_V3 assembly.

File Name: Supplementary Data 2

Description: Whole-genome repeat annotation summary of Lo7_V2 and Lo7_V3 using EDTA.

File Name: Supplementary Data 3

Description: TE annotation of Weining.V3 H1 and H2.

File Name: Supplementary Data 4

Description: RNA-seq quantification of eleven genes located within centromeric regions.

File Name: Supplementary Data 5

Description: Genome size estimation of rye Lo7.

File Name: Supplementary Data 6

Description: Remaining inconsistencies between the optical genome map and pseudomolecules of the Lo7_V3 final assembly.

File Name: Supplementary Data 7

Description: Gene lift-over between Lo7_V2 and Lo7_V3 gene models.

File Name: Supplementary Data 8

Description: The predominant pSat380, pSat571, and pSat118 monomers identified in the 1RS STR region correspond to the pSc200, pSc250, and pSc119.2 repeat sequences, respectively.

File Name: Supplementary Data 9

Description: Representative satellite repeat annotation of the chromosome unanchored region (chrUn) based on TRF analysis.
